# Integration of focal adhesion morphogenesis and polarity by DOCK5 promotes YAP/TAZ-driven drug resistance in TNBC†

**DOI:** 10.1039/d4mo00154k

**Published:** 2025-05-12

**Authors:** Patricia Pascual-Vargas, Mar Arias-Garcia, Theodoros I. Roumeliotis, Jyoti S. Choudhary, Chris Bakal

**Affiliations:** a Chester Beatty Laboratories, Division of Cancer Biology, Institute of Cancer Research 237 Fulham Road London SW3 6JB UK patricia.pascualvargas@icr.ac.uk chris.bakal@icr.ac.uk

## Abstract

YAP and TAZ are transcriptional co-activators that are inhibited by sequestration in the cytoplasm. Cellular signalling pathways integrate soluble, mechanical (cytoskeleton, adhesion), and geometric (cell size, morphology) cues to regulate the translocation of YAP/TAZ to the nucleus. In triple-negative breast cancer (TNBC) cells, both signalling and morphogenesis are frequently rewired, leading to increased YAP/TAZ translocation, which drives proliferation, invasion, and drug resistance. However, whether this increased YAP/TAZ translocation is due to alterations in upstream signalling events or changes in cell morphology remains unclear. To gain insight into YAP/TAZ regulation in TNBC cells, we performed multiplexed quantitative genetic screens for YAP/TAZ localisation and cell shape, enabling us to determine whether changes in YAP/TAZ localisation following gene knockdown could be explained by alterations in cell morphology. These screens revealed that the focal adhesion (FA)-associated RhoGEF DOCK5 is essential for YAP/TAZ nuclear localisation in TNBC cells. DOCK5-defective cells exhibit defects in FA morphogenesis and fail to generate a stable, polarised leading edge, which we propose contributes to impaired YAP/TAZ translocation. Mechanistically, we implicate DOCK5's ability to act as a RacGEF and as a scaffold for NCK/AKT as key to its role in FA morphogenesis. Importantly, DOCK5 is essential for promoting the resistance of LM2 cells to the clinically used MEK inhibitor Binimetinib. Taken together, our findings suggest that DOCK5's role in TNBC cell shape determination drives YAP/TAZ upregulation and drug resistance.

## Abstract 2

DOCK5 acts at focal adhesions in breast cells to establish positive feedback loops *via* CDC42 and RAC The stabilisation of polarity during migration promotes persistent YAP/TAZ activation which we propose drives cancer phenotypes.

## Graphical abstract

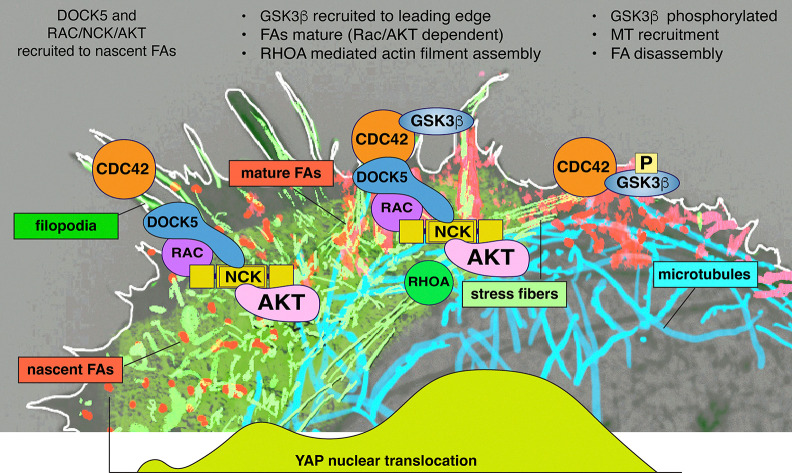

## Introduction

A major question in cell biology is how cells sense changes in cell and tissue morphology to regulate processes such as proliferation, migration, and differentiation. Signalling pathways act as sensors of size and shape by integrating aspects of cell/tissue geometry (‘rulers’), morphological dynamics (‘timers’), and protein/lipid concentration (‘osmometers’), thereby regulating transcriptional, translational, and post-translational processes.^1–5^ However, there remains a poor understanding of how components of these pathways function in cellular space over different time scales to coordinate cell behaviour with changes in shape and size.

First identified in mutagenesis screens that led to tissue overgrowth^6,7^ the Hippo-YAP signalling pathway has emerged as a conserved regulator of transcription in response to changes in cell morphology and size.^3,8–10^ For example, in normal epithelial tissues, injury, damage, and noxious stimuli—such as disruption of cell–cell adhesion and increases in cell-ECM adhesion^10^ (*i.e.* at the leading edge of tissue wounds)—trigger the translocation of the transcriptional co-activators YAP1 and TAZ from the cytoplasm to the nucleus, a process referred to as YAP/TAZ ‘activation’. Activated YAP/TAZ act in concert with transcription factors to regulate cell morphogenesis, invasion, and extracellular matrix (ECM) remodelling, thereby mediating tissue repair and regeneration.^3,5,8–15^ We have previously demonstrated that the dynamics of focal adhesion (FA) assembly and disassembly couple the duration of cell-ECM adhesion to YAP/TAZ translocation, effectively timing a potential repair response.^10^

Increased YAP/TAZ translocation is frequently observed in many cancer subtypes, especially in triple negative breast cancer (TNBC) cells,^16–18^ promoting a number of cancer relevant behaviours such as survival, proliferation, drug resistance, the epithelial-to-mesenchymal transition (EMT), migration, and invasion.^3,4,14,19,20^ ECM remodelling driven by YAP/TAZ activity not only enhances cancer cell migration but also modifies the tumour microenvironment to support metastatic niche formation.^4,14,19,20^ Moreover, the evolved drug resistance of many cancers is due to dysregulation of YAP/TAZ activity.^21–28^ The mechanisms that lead to increased YAP/TAZ translocation in cancers, and especially triple negative breast cancers (TNBC) are very poorly understood. Notably, activating mutations in YAP and TAZ are relatively rare,^4,17,29,30^ suggesting that cancer cells have evolved mechanisms to rewire the regulation of YAP/TAZ translocation dynamics in response to stimuli, rather than constitutively driving nuclear import. Given that YAP/TAZ translocation is highly sensitive to cell shape, size, adhesion, and cytoskeletal organisation, differences in these factors between normal and cancer cells may be key drivers of increased YAP/TAZ activity.

Rho family GTPases, such as CDC42, Rac1/2/3, and RhoA/C are master regulators of cell and tissue morphogenesis.^31,32^ As such their actions have been heavily implicated in the regulation of YAP/TAZ nuclear translocation in both normal and cancer cells.^33–37^ Rho GTPases appear to act both ‘indirectly’ by changing the organization of size, shape, adhesion, or cytoskeletal structures to regulate YAP/TAZ translocation, or ‘directly’ by recruiting and activating the downstream kinases such as p21-activated kinases (PAKs) whose actions converge on YAP/TAZ regulators such as LATS kinases.^3,4,13,14^ We have proposed a model whereby the activity of Rho GTP Exchange Factors (RhoGEFs), such as ARHGEF7/βPIX, act as Focal Adhesions in both ‘indirect’ and ‘direct’ means to regulate YAP/TAZ translocation in normal epithelia during migration.^10^ Specifically, during the initial formation of nascent adhesions at the leading edge of spreading or migrating cells, ARHGEF7 functions as a CDC42 GEF and PAK1 activator, triggering transient YAP/TAZ translocation. Simultaneously, ARHGEF7 promotes FA maturation *via* RacGEF leading to further recruitment of ARHGEF7 and other RhoGEFs to growing FAs, sustaining and amplifying YAP/TAZ nuclear import. FA maturation also facilitates actomyosin contractility *via* RhoA, further enhancing YAP/TAZ translocation. Ultimately, actomyosin contractility and microtubule recruitment promote FA disassembly,^10,38^ terminating YAP/TAZ translocation signals. Thus, RhoGEF/Rho signalling provides a mechanism by which YAP/TAZ translocation is directly coupled to FA morphogenesis and leading-edge dynamics.

While we demonstrated FA-ARHGEF7 activity as a regulator of YAP/TAZ translocation in normal breast epithelial cells, we found little evidence of this mechanism in TNBC breast cancer cells^10^ suggesting that TNBC cells have different mechanisms to regulate YAP/TAZ translocation.

There are likely multiple ways in which RhoGEFs and/or Rho GTPase Activating Proteins (RhoGAPs) drive YAP/TAZ translocation in cancer. As essential and conserved regulators of morphogenesis, cytoskeletal organisation, and adhesion, their dysregulation in cancer cells could have profound effects on YAP/TAZ translocation rates. Notably, in cases where YAP/TAZ upregulation drives drug resistance, increased YAP/TAZ activation is almost always coincident with changes in cell shape and actin reorganisation.^22,24,28,39–42^ Moreover, we recently demonstrated that in breast cancer cells, YAP/TAZ translocation rates are regulated by signalling pathways such as RAS/MAPK, ensuring that nuclear YAP/TAZ levels remain robust to changes in cell size.^1^ Since dysregulation of RAS/MAPK signalling is a common event in cancer, it could affect the relationship between YAP/TAZ and shape and size. While the mechanisms underlying this sensing remain to be elucidated, Rho-family GTPases, as conserved regulators of signalling in response to geometric and mechanical cues, are likely to be involved in coupling size to YAP/TAZ translocation.

To elucidate the basis for increased YAP/TAZ translocation in drug-resistant metastatic breast cancer LM2 cells, we performed multiplexed high-throughput genetic screens, systematically knocking down the majority of human RhoGEFs and RhoGAPs while simultaneously measuring YAP/TAZ nuclear localisation. To determine whether differences in YAP/TAZ translocation could be attributed to changes in cell shape, we also assessed single-cell morphology. These screens identified the focal adhesion RacGEF DOCK5 (dedicator of cytokinesis 5) as essential for YAP/TAZ translocation in drug-resistant metastatic breast cancer cells. The morphology of DOCK5-deficient cells was phenotypically similar to CDC42- and YAP1-depleted cells, suggesting a shared pathway. Indeed, DOCK5 exhibited synthetic interactions with both CDC42 and YAP1. Chemical perturbation indicated that DOCK5's RacGEF activity contributes to YAP/TAZ translocation, but we also found evidence for a non-catalytic role of DOCK5 in YAP/TAZ regulation.

We further demonstrated that DOCK5 is essential for TNBC cell migration and 3D invasion. Migration defects in DOCK5-deficient cells were partially attributable to FA morphogenesis defects and an inability to maintain stable protrusions. Proteome-wide mass spectrometry analysis revealed that DOCK5 depletion leads to significant decreases in GSK3β protein levels, which stabilises the polarised leading edge in migrating cells. We propose that following the initiation of a leading edge by CDC42, DOCK5 is recruited to nascent FAs, where it acts as a RacGEF and a non-catalytic activator of AKT. This stabilises FAs, reinforcing positive feedback through further DOCK5 and GEF recruitment while promoting GSK3β phosphorylation to maintain the leading edge. These feedback loops ensure the formation of a single leading edge, akin to symmetry breaking in yeast. The formation of a FA-rich leading edge promotes YAP/TAZ translocation in a manner dependent on CDC42, DOCK5, AKT, and RhoA activity, effectively ‘timing’ YAP/TAZ translocation with leading-edge dynamics. Crucially, as DOCK5 is essential for LM2 cell resistance to the MEK inhibitor Binimetinib, this suggests that a polarised migratory morphology itself is a driver of drug resistance.

## Results

### DOCK5 depletion lowers YAP/TAZ nuclear translocation

LM2 cells are a metastatic derivative of MDA-MB-231 TNBC cells.^43^ Using an antibody (63.7 sc-101199, Santa Cruz Biotechnology) that recognises both YAP1 and TAZ in immunofluorescence (IF) and western blot, we detected YAP/TAZ proteins predominantly in the nucleus of proliferating LM2 cells (Fig. S1, ESI†).^44^ In fact, compared to the TNBC cell lines hs578t and SUM159, and the non-metastatic mammary carcinoma cell line T47D, LM2 cells had much higher nuclear YAP/TAZ at sub-confluent densities (Fig. S2A, ESI†). Increasing the cell density of LM2 cell populations to high levels (2.0 × 10^5^ cells per ml, 6000 cells per well) reduced YAP/TAZ nuclear translocation in LM2 cells, demonstrating that YAP/TAZ in LM2 cells was partially sensitive to cell numbers as observed in normal tissues^10,45^ (Fig. S2A, ESI†). High basal levels of nuclear YAP/TAZ in sub-confluent LM2 cells also contrasted markedly to those observed in MCF10A normal breast epithelial cells (Fig. S2A, ESI†).^10^ Throughout this study we used LM2 cells at low density (3.3 × 10^4^ cells per ml, 1000 cells per well). The higher baseline levels of nuclear YAP/TAZ in sub-confluent metastatic LM2 cells correlate with observations that YAP/TAZ are drivers of metastasis^11,46–48^

To identify regulators of Rho-family GTPases that contribute to the regulation of YAP/TAZ dynamics in TNBC cells, we systematically depleted 82 RhoGEFs, 67 GAPs and 19 Rho GTPases in LM2 cells, and quantified the ratio of nuclear to cytoplasmic YAP/TAZ as a proxy for YAP/TAZ activation.^3,10^ LATS1, YAP1 and ECT2 siRNAs were used as positive controls:^44^ si*LATS1/2* for our ability to assess YAP/TAZ localisation, as YAP/TAZ nuclear localisation is expected to increase in the absence of LATS1/2;^49^ si*YAP1* for our ability to detect YAP/TAZ protein; and si*ECT2* to assess transfection efficiency as ECT2 depletion results in multinucleate cells.^50,51^

**Fig. 1:**
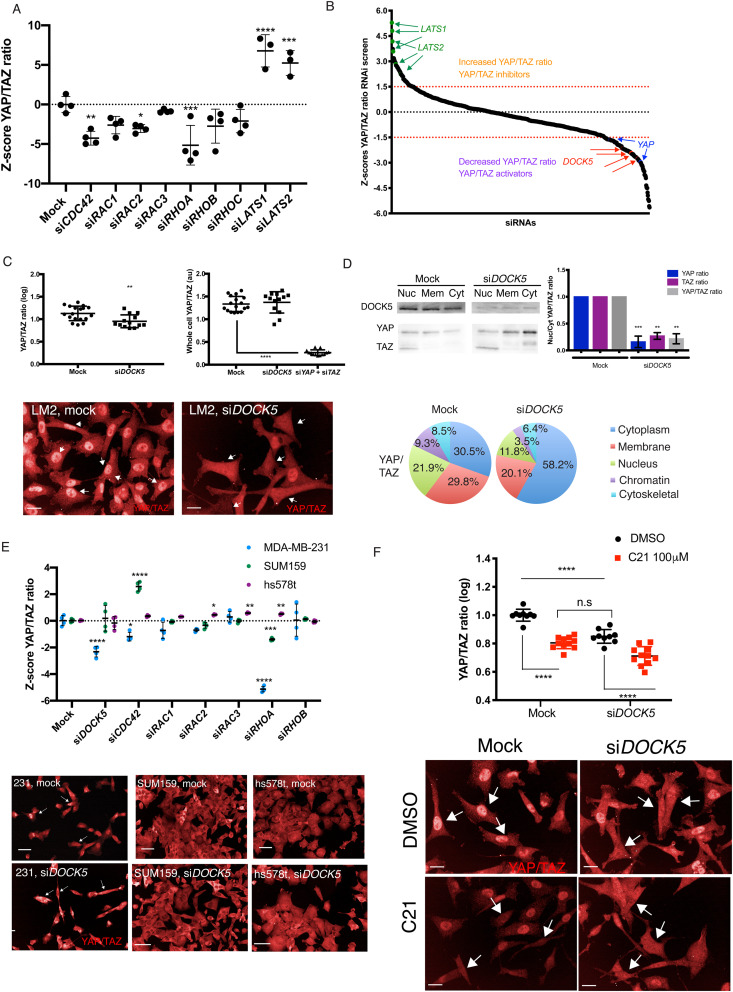
DOCK5 regulates YAP/TAZ localisation. (A) Graph depicting *Z*-scores of log YAP/TAZ ratios following knockdown of Rho GTPases. si*LATS1/2* included as controls for YAP/TAZ translocation. mean ± s.d (*n* = 3–4 wells per condition, 1000 cells per well, one-way ANOVA to mock). *Z*-Scores were then calculated per screen using the average and standard deviation of all mock transfected cells. Each well was assigned a QMS consisting of the *Z*-score for each shape (B) *Z*-scores per well for YAP/TAZ ratio for OTP siRNA screen in LM2. Above 1.5 (red dotted line) are siRNAs which are hits for high YAP/TAZ ratio and thus can be considered inhibitors of YAP/TAZ. Below −1.5 (red dotted line) are siRNAs which are hits for low YAP/TAZ ratio and thus can be considered YAP/TAZ activators. *LATS1/2* and *YAP* siRNAs included as controls for high and low YAP/TAZ ratios are shown. Four individual replicates for si*DOCK5* are shown. (C) Representative images showing YAP/TAZ staining for mock transfected and si*DOCK5* transfected LM2 cells (40×). Arrows point to cells with high YAP/TAZ ratio (mock) and low YAP/TAZ ratio (si*DOCK5*). Scale bar is 20 μm. Graphs showing quantification of YAP/TAZ ratio, and whole cell YAP/TAZ levels in mock *versus* si*DOCK5.* si*YAP* + si*TAZ* show YAP/TAZ antibody is working appropriately. mean ± s.d (*n* = 3) independent biological repeats separate from screen data, dots represent individual wells, Student's *t*-test (left) and ANOVA (right). (D) Validation of si*DOCK5* resulting in a decrease in YAP/TAZ ratio *via* subcellular fractionation. Representative western blot of subcellular fractions for mock and si*DOCK5*. Nuc: nuclear fraction. Mem: membrane fraction. Cyt: cytoplasm fraction. Blotted for DOCK5, YAP and TAZ. Same amounts of protein loaded (6.5 ng). Quantification of ratio of YAP/TAZ in the nucleus to the cytoplasm fraction from western blots of subcellular fractionations. mean ± s.d (*n* = 2). Student's *t*-test. Representative distribution of YAP/TAZ in all fractions with and without *DOCK5*. (E) Graph depicting *Z*-scores of log YAP/TAZ ratios following knockdown of Rho GTPases and DOCK5 in OTP screens in MDA-MB-231, SUM159 and hs578t. Mean ± s.d (*n* = 3–4 wells per condition, 1000 cells per well, one-way ANOVA to mock per cell line). Representative images for DOCK5 knockdown and mock transfection in each cell line. Scale bar is 50 μm. (F) Immunofluorescence images and quantification of YAP/TAZ localisation in mock or si*DOCK5* transfected cells treated with DMSO or DOCK5 inhibitor C21. Arrows indicate representative cells for each condition. Mean ± s.d (*n* = 9 wells/condition, 1000 cells per well, one-way ANOVA). Significance for all panels: * *P* < 0.05, ** *P* < 0.001 *** *P* < 0.001, **** *P* < 0.0001. Imaged at 40×. Scale bar is 20 μm.

Knocking down individual Rho-family GTPases had distinct effects on YAP/TAZ nuclear translocation. Specifically, knockdown of CDC42, and RHOA resulted in a significant drop in nuclear YAP/TAZ (Fig. 1A). Knockdown of either RAC1 or RAC3 led to statistically insignificant decreases in nuclear YAP/TAZ, while knockdown of RAC2 led to a moderate decrease (Fig. 1A). Thus, the basal levels of YAP/TAZ nuclear translocation observed in LM2 TNBC cells are partially due to the activation of CDC42 and RHOA. However, it is possible that that RAC1 and RAC2, but likely not RAC3,^52^ promote YAP/TAZ nuclear translocation in LM2 cells in a partially redundant manner.^53^

To identify RhoGEFs that might be responsible for basal activation of YAP/TAZ in LM2 cells, we focused on RhoGEF siRNAs that reproducibly decreased nuclear YAP/TAZ levels. DOCK5 siRNA was the most consistent hit for siRNAs that caused low YAP/TAZ ratio (Fig. 1B), suggesting it is required for basal levels of YAP/TAZ activation in LM2 cells. All (4/4) replicates of DOCK5 siRNA were hits in our initial screen (Table S1, ESI†). We performed the same screen using another siRNA library (Dharmacon siGENOME; siG), where again 4 out of 4 replicates of a DOCK5 siRNA pool with distinct sequences from the OTP pool decreased YAP/TAZ nuclear levels (Fig. S2B, *Z*-scores per well: −1.73, −2.33, −2.77, −0.79, ESI†). Notably, using both the OTP and siGENOME siRNA, depleting DOCK5 consistently reduced YAP/TAZ ratio without changing the total levels of YAP/TAZ (Fig. 1C, D and Fig. S2C, ESI†).

To validate that decreases in YAP/TAZ ratio were due specifically to DOCK5 depletion, we quantified mRNA expression levels following OTP and siGENOME knockdown of DOCK5 by quantitative RT-PCR and found that *DOCK5* mRNA expression levels were significantly reduced using either pool (Fig. S2D, ESI†). We also tested the ability of each individual sequence (4 siRNAs per pool) from the two different siRNA pools (OTP and siG) to knockdown *DOCK5* mRNA and found that all 8 siRNAs did so effectively (Fig. S1E, ESI†). Further, 7/8 single siRNAs significantly reduced YAP/TAZ ratio and none but siG3 reduced total YAP/TAZ levels (Fig. S2C, ESI†). Because it is virtually impossible that 7/8 DOCK5 siRNAs would reduce YAP/TAZ translocation through any other manner than by depleting DOCK5 mRNA and protein these data prove that DOCK5 promotes YAP/TAZ nuclear translocation in LM2 cells.

To corroborate our immunofluorescence imaging-based observations that DOCK5 depletion affects YAP/TAZ nuclear translocation, we performed subcellular fractionation of mock and DOCK5 depleted LM2 cells, and used the same antibody (sc-101199) used for immunofluorescence to probe for YAP and TAZ protein. In DOCK5 depleted cells the cytoplasmic fraction had significantly higher levels of both YAP and TAZ, compared to the same fraction from mock transfected cells. Indeed, we quantified the ratios of nuclear to cytoplasmic YAP and TAZ individually, and together (YAP/TAZ), all of which were significantly lower (Fig. 1D). These results show that DOCK5 depletion results in decreases of both nuclear YAP and TAZ. Importantly, depletion of DOCK5 appears to limit translocation without affecting YAP/TAZ stability.

To understand whether the effect of DOCK5 depletion is specific to LM2 cells, or common across TNBC cell lines, we performed the same RNAi screens as for LM2, in the parental MDA-MB-231 cell line as well as two weakly metastatic TNBC cell lines: SUM159 and hs578t. We found that DOCK5 depletion also decreased YAP/TAZ nuclear translocation in both screens (siGENOME and OTP) using the parental cell MDA-MB-231 (Fig. S3, ESI†). Further, this decrease was also observed with RHOA siRNA in MDA-MB-231(Fig. 1E). DOCK5 was not a hit for YAP/TAZ translocation in SUM159 and hs578t cells (OTP, Fig. 1E). This suggests that DOCK5 plays a role in more strongly metastatic MDA-MB-231 and LM2 TNBC subtypes, but less so in weakly metastatic SUM159 and hs578t lines.^54^

To test the role of DOCK5 RAC GEF activity on YAP/TAZ translocation we used C21, an inhibitor which specifically blocks DOCK5's DHR2 catalytic domain by hindering the interaction between DOCK5 and RAC1.^55–57^ In particular, C21 has been demonstrated not be a general RAC inhibitor as it does not affect the exchange activity of TRIO, a RacGEF unrelated to DOCK5.^55^ Treatment with C21 decreased YAP/TAZ by the same amount as DOCK5 siRNA (Fig. 1F). Thus DOCK5 catalytic activity on RAC-type GTPases is an essential component of how DOCK5 promotes YAP/TAZ nuclear translocation.

### DOCK5 contributes to proliferation and resistance to MEK inhibitors

**Fig. 2:**
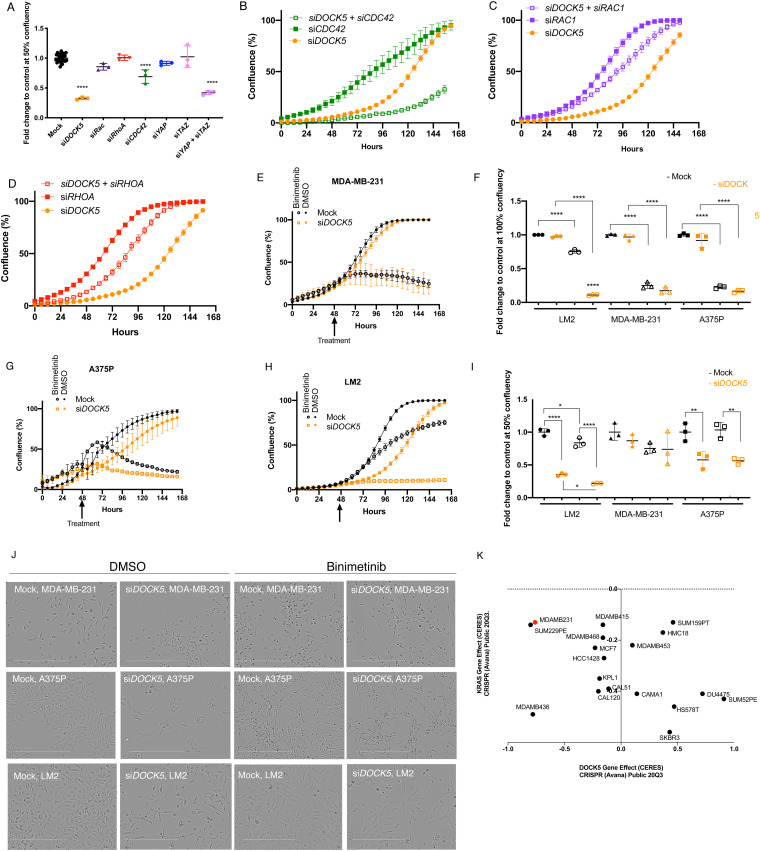
DOCK5 contributes to growth and resistance to MEK inhibitors. (A) Fold change to control mock transfected wells at 50% confluency for growth curves (Fig. 2B–D and Fig. S4, ESI†). Each data point is from one well. mean ± s.d, Student's *t*-test. (B)–(D) Growth curves for (B) si*DOCK5*, si*CDC42*, si*DOCK5* + si*CDC42*, (C) si*DOCK5*, si*RAC1*, si*DOCK5* + si*RAC1*, (D) si*DOCK5*, si*RHOA*, si*DOCK5* + si*RHOA* transfected cells. Averages per well are shown. (E), (G) and (H) Growth curves and (J) representative images at 50% confluency for mock and si*DOCK5* transfected cells treated with DMSO (closed circle, square) and 10 μM final MEKi Binimetinib (open circle, square) for (E) MDA-MB-231, (G) A375P, (H) LM2. (F) and (I) Fold change to control mock transfected wells at 100% and 50% confluency respectively for LM2, MDA-MB-231, and A375 mock and si*DOCK5* transfected cells treated with DMSO or Binimetinib. Each data point is from one well. mean ± s.d. Scale bar is 400 μm. All experiments performed in triplicate, representative experiment shown with *n* = 3 wells per condition. One-way ANOVA. For all panels: * *P* < 0.05, ** *P* < 0.01, *** *P* < 0.001, **** *P* < 0.0001. (K) Data plotted from Depmap website based on dependency of breast cancer cell lines to KRAS or DOCK5 knockdown. A CERES (computational method to estimate gene-dependency levels from CRISPR-Cas9 essentiality screens)^64^ a lower score indicates that cell line will be more dependent on that gene.

In many cell types, YAP/TAZ acts as a transcriptional co-activator essential for survival and/or proliferation.^28,49,58–63^ To determine if DOCK5, Rho GTPases, and basal YAP/TAZ activity contribute to the survival of LM2 TNBC cells we performed growth assays over 160 hours. We normalized confluency values per condition to that of mock transfected cells per well and compared fold change to mock at 50% confluency (Methods). DOCK5 depleted cells proliferated at a slower rate as there were significantly less cells (68% less) following 60 hours post-transfection of DOCK5 siRNA where the mock population had reached 50% confluency (Fig. 2A). Furthermore, DOCK5 depleted cells took 32 hours more than mock transfected cells to reach confluence (Fig. 2E and Fig. S4A, ESI†). Cells where CDC42 was depleted by siRNA also proliferated at a slower rate than mock transfected cells (Fig. 2A, B and Fig. S4B, ESI†). But cells depleted of RHOA, RAC1, YAP, or TAZ proliferated at the same rate as mock transfected cells (Fig. 2A and Fig. S4C–F, ESI†). However, the proliferation of cells simultaneously depleted of both YAP1 and TAZ was significantly impaired (Fig. 2A and Fig. S4G, ESI†). We propose that DOCK5 and CDC42 promote proliferation of LM2 cells at least in part by promoting the nuclear translocation of both YAP1 and TAZ, though we cannot exclude the possibility that DOCK5 and/or CDC42 regulate proliferation independently of YAP/TAZ activation.

To determine if DOCK5 regulates proliferation in a fashion dependent on Rho GTPases, we assessed whether DOCK5 depletion is synthetically lethal with depletion of RAC1, RHOA, and CDC42. Indeed, DOCK5 and CDC42 exhibit synthetic lethality (Fig. 2B), such that at 156 hours when mock transfected cells were confluent, CDC42 and DOCK5 depleted cells were at 32% confluency. In contrast, depletion of either RAC1 or RHOA with DOCK5 partially rescued the lethality of DOCK5 depletion (Fig. 2C and D respectively). We conclude that DOCK5 exhibits genetic interactions with CDC42, RAC1, and RHOA; a negative/aggravating interaction with CDC42 and positive/alleviating interactions with RAC1 and RHOA. Positive/alleviating interactions are symptomatic of “within pathway” interactions,^65–67^ which is consistent with the fact that DOCK5 activates RAC GTPases.^56,68–70^ That DOCK5 has a negative/aggravating interaction with CDC42 suggests they function independently of each other – *i.e.* in parallel pathways, and is consistent with the idea that DOCK5 is unlikely to be a CDC42 GEF in LM2 cells.^55,57,71^

LM2 cells harbour mutant KRAS (G13D),^72^ which activates the kinases MEK and ERK, driving proliferation and survival.^73,74^ However, while MEK inhibition (MEKi) effectively kills some cancer cells such as melanoma cells, breast cancer cells are often resistant to MEKi.^75,76^ Because MEKi resistance in melanoma, pancreatic and non-small lung cancer (NSLC) is often due to YAP/TAZ activation,^25,26,77,78^ we assessed whether the MEKi resistance of LM2 cells was due to DOCK5 and/or YAP/TAZ activity. To test this hypothesis, we first treated LM2, MDA-MB-231 (from which LM2 cells are derived), and A375p melanoma cells (which harbour activated mutant BRAF) with the MEKi Binimetinib. MEKi significantly inhibited survival in MDA-MB-231 (Fig. 2E and F) and A375p (Fig. 2F and G) cells, but only had a modest effect on the survival of LM2 cells (Fig. 2F and H). DOCK5 depletion did not have any effect on the survival or proliferation of MDA-MB-231 or A375p cells in the absence or presence of MEKi (Fig. 2F, I and J). However, depletion of DOCK5 and MEKi treatment in LM2 cells led to a synergistic effect on proliferation or survival where confluency stalled at 10% for the whole duration at the assay (Fig. 2H–J). Thus, MEKi aggravates DOCK5 depletion suggesting that ERK and DOCK5 are functioning in compensatory pathways, and DOCK5 is essential for resistance of LM2 cells to MEK inhibition.

Supporting the idea that a chemical-genetic interaction between YAP/TAZ and RAS-ERK signalling exists, mining of the DEPMAP database reveals that CRISPR-mediated depletion of DOCK5 and KRAS affects cell viability in a significantly similar manner across a large panel of cell lines (Fig. 2K); 0.4 Pearson correlation between effects of KRAS and DOCK5 depletion across cell lines as determined by DEPMAP profiling.^64^ These data support the idea that both DOCK5-YAP1 and RAS-ERK pathways signalling promote survival in breast cancer cells, and that while inhibition of one axis can blunt survival, simultaneous inhibition of both is lethal.

### DOCK5 depletion reduces 3D invasion

**Fig. 3:**
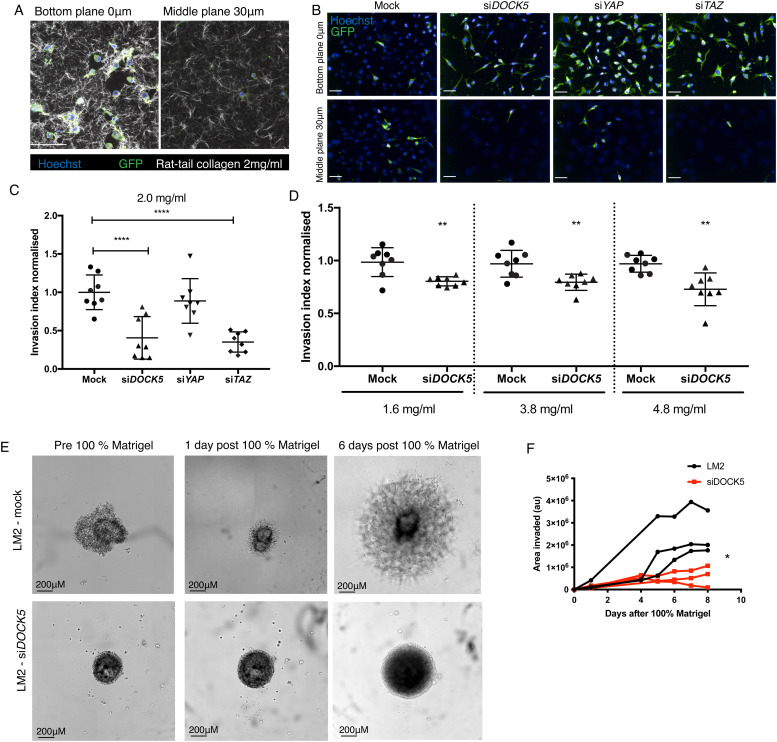
DOCK5 depletion reduces 3D invasion. (A) Representative 2-photon images of LM2 cells (GFP) stained for DNA (blue) mixed with rat-tail collagen I at 2 mg ml^−1^ (white) at the bottom of the plate (0 μm plane) and the middle (30 μm plane). (B) Representative confocal spinning disk images (12 fields per well) for knockdowns shown in (C) at 0 μm and 30 μm. (C) Graph shows quantification of invasion index for knockdowns in rat-tail collagen I at 2 mg ml^−1^. Invasion index is indicative of the number of cells in middle planes divided by total number of cells in all planes quantified, including bottom plane. Mean ± sd (*n* > 3 wells per condition, 1000 cells per well. ANOVA, **** *P* < 0.0001). (D) Invasion index of mock *versus* si*DOCK5* transfected cells at collagen concentrations: 1.6 mg ml^−1^, 3.8 mg ml^−1^, and 4.8 mg ml^−1^. Mean ± sd (*n* > 3 wells per condition, 1000 cells per well, ANOVA, ** *P* < 0.01). Scale bars, 100 μm. (E) Representative images of mock transfected cells and si*DOCK5* transfected cell spheroids 48 hours post transfection and pre 100% Matrigel (left), and one (middle) and six (right) days post 100% Matrigel addition. Scale bar is 200 μm. (F) Graph shows quantification of area of Matrigel in arbitrary units over 8 days post adding 100% Matrigel to the spheroids. Mock transfected cells in black, si*DOCK5* transfected cells in red. Each line represents one well. *n* = 3 wells. Statistical significance obtained by performing student *t*-test on row means (* *P* = 0.0150).

To determine if DOCK5 contributes to the ability of LM2 cells to invade 3D tissues, we quantified the extent to which DOCK5 depleted cells invade rat tail collagen I (Col-I) gels. Cells typically invaded from the bottom of the plate into depths of 30 μM in the collagen gel within 24 hours (Fig. 3A and B). We quantified cell invasion by calculating the invasion index, defined as the number of cells at various planes within the gel, divided by the total number of cells in all planes plus the bottom plane. In a 2.0 mg ml^−1^ rat-tail Col-I gel, DOCK5 depletion significantly reduced invasion (Fig. 3C). Further, depleting TAZ but not YAP significantly reduced invasion, by the same amount as depleting DOCK5. The similarity in the phenotypes following DOCK5 and TAZ depletion strongly suggests that DOCK5 regulates TAZ as well as YAP translocation to promote invasion.

Because collagen pore size has been shown to be inversely proportional to collagen concentration,^79^ we used collagen gels of different concentrations as a way of varying pore size. Depleting DOCK5 consistently reduced invasion across a Col-I density range of 1.6 to 4.8 mg ml^−1^ compared to mock transfected cells (Fig. 3D). Overall, these data suggest that DOCK5 is required for LM2 single-cell invasion in rat tail collagen matrices independently of pore size.

To explore whether DOCK5 is required for invasion of cells in multicellular bodies – *i.e.* in tumour models, we generated spheroids and quantified cell invasion into 100% Matrigel (Methods). Mock transfected LM2 cells invaded into the gel progressively, while cells lacking DOCK5 did not invade at all (Fig. 3E and F). Overall, this suggests that LM2 cells require DOCK5 to invade into 3D structures.

### DOCK5 depletion results in polarity and adhesion defects

**Fig. 4:**
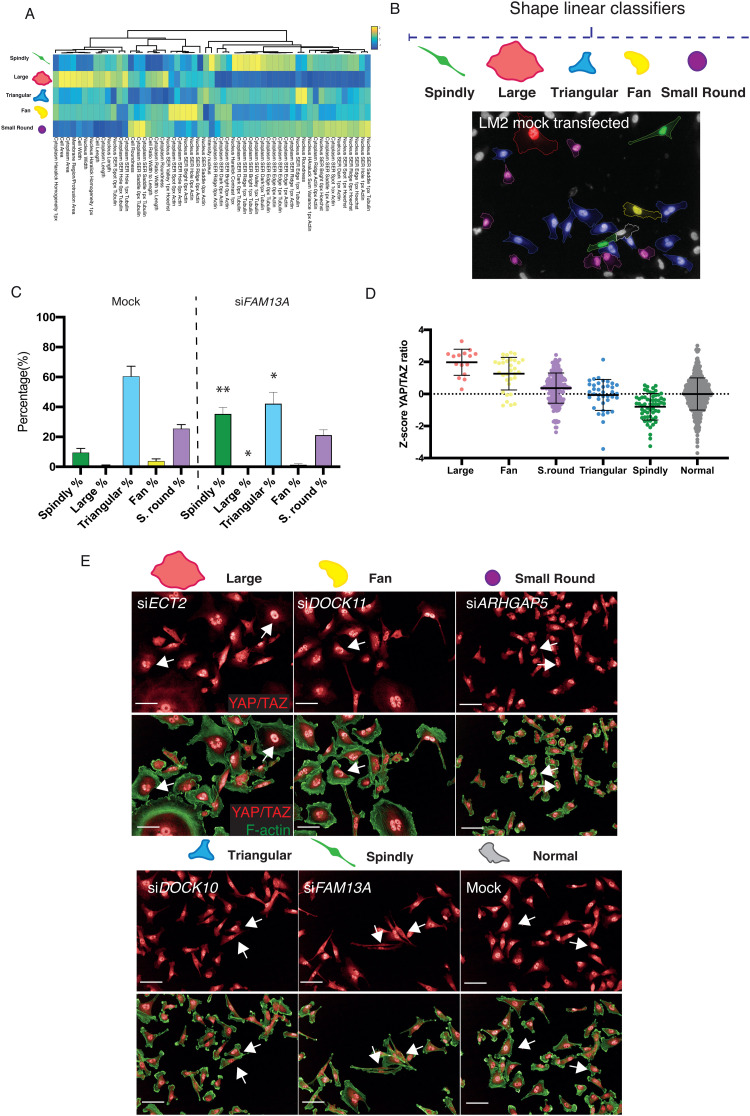
Shape classification and relationship with YAP/TAZ. (A) Heat map and dendrogram of the features describing each shape and which Columbus software used to classify cells into the five visually distinctive reference shapes, as well as their contribution, using single cell data. Yellow and blue represent a high and low score respectively in arbitrary units. (B) Images from one field of view from mock transfected well with cells coloured by their classified shape by software showing highly heterogenous population, and (C) the percentage of cells classified into each shape in one sample mock transfected well and for an siRNA which was a top hits for spindly shape, depicting a rearrangement in the shapes seen for mock-transfected cells. Mean per well ± sd are shown (*n* > 3, 1000 cells per well, student's *t*-test on row for each shape between si*FAM13A* and mock-transfected cells, * *P* < 0.05, ** *P* < 0.01). (D) Graph depicting *Z*-score of log10 ratio of nuclear to ring region YAP/TAZ for single LM2 cells following transfection with siRNA which results in the biggest enrichment for each particular shape: si*ECT2* for large, si*DOCK11* for fan, si*ARHGAP5* for small round, si*DOCK10* for triangular, si*FAM13A* for spindly. Mean ± sd are shown. Data taken from at least 5 fields of view from 2 wells. (E) Images of population from which single cell data shown in (D) were taken. Arrows indicate examples of nuclear YAP/TAZ for each shape. Images stained for YAP/TAZ (red) and F-actin (green). Scale bar is 50 μm.

Changes in YAP/TAZ localization following systematic RNAi of RhoGEFs could be due to changes in cell/membrane morphology, size, cytoskeletal organization, or adhesion.^10^ Alternatively, it is formally possible differences in YAP/TAZ translocation could be due to alteration in signalling that is independent of these factors. To differentiate between these two possibilities we quantified the cell shape of single LM2 cells following depletion of 149 RhoGEF/GAPs and 19 Rho GTPases to generate quantitative morphological signatures or “QMSs”.^80^ In this case, QMSs describe the percentage of cells with a particular shape in an RNAi-treated population, and thus account for the phenotypic heterogeneity observed in different cell lines.^81^ To generate QMSs, we measured 127 morphological and texture features for single cells in the screens, and trained linear classifiers to identify five visually distinctive reference shapes. The percentage of cells binned into each sub-population is a QMS for that specific RNAi (Methods). The five visually distinct shapes we used to characterize populations are: (1) ‘spindly’ – elongated cells with typically two protrusions; (2) ‘large round’ – spread cells of a large area which are often circular; (3) ‘triangular’ – cells with three distinct protrusions; (4) ‘fan’ – asymmetrically-shaped cells with nucleus to one side; and (5) ‘small round’ – cells which have a small area and high roundness (Fig. 4A and B). LM2 cells are highly heterogeneous, and in a mock-transfected population 9.4% cells are classified as spindly, 0.9% as large round, 60.4% as triangular, 3.7% as fan, and 25.5% as small round (Fig. 4C). Depletion of different RhoGEF and RhoGAP genes by siRNA resulted in significant changes in the number of cells in each sub-population. For example, depletion of FAM13A resulted in a significant increase in spindly and triangular cells, and a significant decrease in large cells with: 35.3% of cells being classified as spindly, 0.3% as large, 42.1% as triangular, 1.3% as fan and 21.1% as small round (Fig. 4C).

We also developed a classifier for ‘normal’ cells (Methods, Fig. S5, ESI†). This allowed us to identify siRNAs which had significant effects on cell shape based on whether they significantly depleted the percentage of normal cells compared to mock (*Z*-score equal to or below −1 for normal classifier). In essence we could thus describe the penetrance of each genetic perturbation.^81^ 89 siRNAs resulted in significant depletion of normal cells; of which 79 were RhoGEF/GAPs, 7 were Rho GTPases, and 3 (si*LATS1*, si*LATS2* and si*YAP1*) were controls for YAP/TAZ translocation.

To determine if cell shape changes regulate YAP/TAZ in LM2 cells, for example following depletion of RhoGEFs or RhoGAPs, we next asked whether exemplars of different cell shapes that were used to derive classifiers had predictable variability in YAP/TAZ nuclear translocation. Effectively asking the question, does shape predict YAP/TAZ dynamics? Indeed, different classifier shapes had significantly different levels of nuclear YAP/TAZ ranging from high nuclear YAP/TAZ levels in polarised “fan” cells with broad lamellipodia to lower levels in “spindly” unpolarised cells (Fig. 4D and E). Thus as in other cell types,^3,4,10,14,44^ in LM2 cells morphology can largely predict YAP/TAZ translocation dynamics.

**Fig. 5:**
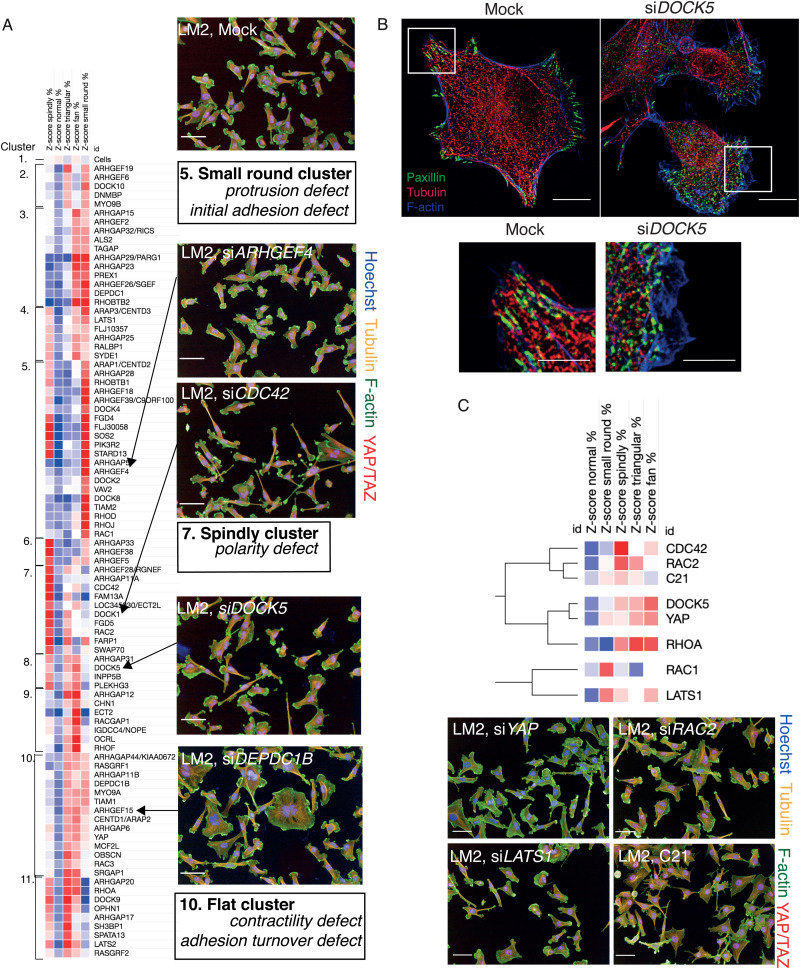
DOCK5 regulates shape. (A) Clustering of high-dimensional quantitative signatures based on the effect of RhoGEF/GAPs and Rho GTPase siRNAs on the TNBC cell line LM2. Columns are *Z*-score values of the percentage of cells classified into that shape when said siRNA is depleted. Red and blue indicate high and low values respectively. Clustering performed using Pearson correlation. 10 clusters and a singleton were identified and defined as genes grouped at the highest node of clustering with a value of uncentered Pearson correlation coefficient above 0.73. Numbering indicates clusters and images are representative for mock and siRNAs which enrich for a particular shape in example clusters. Staining shows DNA (blue), tubulin (yellow), F-actin (green) and YAP/TAZ (red). Scale bar is 50 μm. (B) Representative images for a mock “normal” and an si*DOCK5* transfected cell imaged on SoRa. Staining shows Paxillin (green), α-tubulin (red), F-actin (blue). Square represents zoom in below. Scale bar is 10 μm, zoom in 5 μm. (C) Clustering of QMS for siRNAs shown as well as C21 treatment at a Pearson correlation of 0.74. Images for si*RAC2*, C21, si*LATS1*, and si*YAP* are shown, stained for DNA (blue), tubulin (yellow), F-actin (green) and YAP/TAZ (red). Scale bar is 50 μm.

To identify how different RhoGEFs, RhoGAPs, and Rho GTPases, and in particular DOCK5, regulate LM2 cell shape specifically, we performed hierarchical clustering on 5-shape QMSs for shape hits (<−1 *Z*-score for normal classifier) in the LM2 OTP screen, and defined “phenoclusters” as genes grouped at the highest node of clustering with a Pearson Correlation Coefficient (PCC) of >0.73 (Fig. 5A and Table S2, ESI†). This resulted in 10 multigene clusters and 1 singleton formed by mock transfected LM2 cells.

The QMS of DOCK5 siRNA belonged to cluster 8 which is characterized by increases in fan, spindly, and triangular shapes (Fig. 5A). Cells following DOCK5 depletion also closely resembles spindle/unpolarised shapes found in cluster 7 following depletion of the canonical and conserved polarity regulator CDC42^33,82–85^ (Fig. 5A) and cluster 9 – which is characterised by very large flat cells, such as following depletion of YAP/TAZ. Importantly, the relatively high similarity in morphology following depletion of DOCK5, CDC42 and YAP/TAZ supports the idea that DOCK5 and CDC42 regulate YAP/TAZ translocation.

We next asked whether the QMS of C21 treated cells is similar to that of DOCK5, YAP1, or LATS1 depleted cells. Because C21 inhibits GEF activity of DOCK5 towards RAC-type GTPases, we compared the DOCK5 QMS with QMSs following knockdown of CDC42, RAC1, RAC2, and RHOA. Hierarchical clustering of QMSs revealed that the QMS of C21 is similar to that of RAC2 and CDC42 depleted cells (Pearson correlation coefficient (PCC) > 0.77), but only partially similar to the QMS of DOCK5 and YAP1 depleted cells (PCC > 0.40) (Fig. 5C). Thus, DOCK5 depletion is not phenotypically identical in terms of shape to disruption of DOCK5 GEF activity and suggests that DOCK5 mediated regulation of shape occurs both *via* GEF mediated regulation of RAC-type GTPase (*i.e.* RAC1 and/or RAC2), as well as by acting as a scaffold.

### DOCK5 depletion alters the levels of polarity proteins

**Fig. 6:**
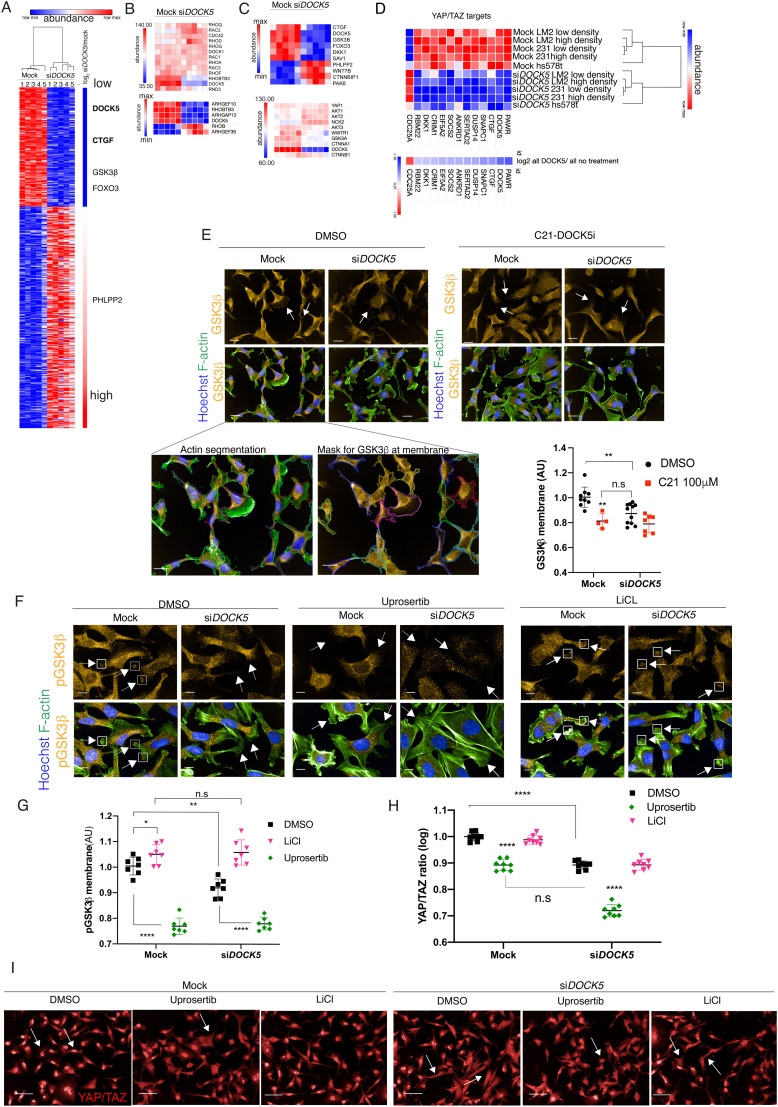
DOCK5 depletion alters the levels of polarity proteins. (A) Heat map of proteins significantly up or downregulated following *DOCK5* knockdown *via* siRNA in LM2,^1,2^ MDA-MB-231^3,4^ and hs578t.^5^ Significance based on log 2 ratios for protein levels of si*DOCK5* v mock being above 0.5 and below −0.5 and permutation FDR < 0.05 (*t*-test). Red and blue indicate high and low protein abundance levels (row max to min respectively). Proteins up and downregulated based on log 2 value. Proteins involved in regulating polarity and AKT signaling pathways shown are shown as well as CTGF. YAP/TAZ targets shown in bold (B) top protein abundance levels of Rho GTPases and DOCK1 that were unchanged with si*DOCK5* abundance levels for comparison. Bottom protein levels of Rho GTPases and RhoGEF/GAPs which were significantly upregulated or downregulated upon DOCK5 knockdown. Row max (red) to min (blue). (C) Top: Heat map of protein levels of proteins regulating polarity that were significantly up and down regulated. Values normalised to cell line. Row max (red) to min (blue). Bottom: Abundance levels of proteins of interest that were not significantly up or downregulated, with si*DOCK5* abundance levels for comparison. (D) Heat map of proteins significantly up or downregulated following *DOCK5* knockdown *via* siRNA in TNBC cell lines which have been shown to be YAP/TAZ target genes in MDA-MB-231. Red and blue indicate high and low protein abundance levels (row max to min respectively). Log 2 value below. (E) Representative images and immunofluorescence quantification of GSK3β at the membrane in mock and si*DOCK5* transfected cells in DMSO or DOCK5 inhibitor C21 Mean per well ± sd (*n* > 3 wells/condition, 1000 cells per well; ANOVA). White arrows indicate GSK3β at the membrane (mock) and lack thereof (si*DOCK5* and C21). GSK3β imaged at 40×, scale bar, 20 μm. Inserts show actin segmentation and mask used to quantify GSK3β at the membrane. (F) Representative images and (G) immunofluorescence quantification of pGSK3β at the membrane in mock and si*DOCK5* transfected cells treated with DMSO (black), 5 mM LiCl (pink) or 2 μM final Uprosertib (green). Mean per well ± sd are shown (*n* > 3, 1000 cells per well, 2 ways ANOVA, Tukey's multiple comparison's test * *P* < 0.05, ** *P* < 0.01, *** *P* < 0.001, **** *P* < 0.0001). White arrows indicate pGSK3β at the membrane (mock and LiCl) and lack thereof (si*DOCK5* and Uprosertib). pGSK3β imaged at 60× with water objective (NA = 1.2). Scale bar, 10 μm. (H) Immunofluorescence quantifications of YAP/TAZ ratio of mock and si*DOCK5* transfected cells treated with DMSO (black), 5 mM LiCl (pink) or 2 μM final Uprosertib (green). Mean per well ± sd are shown (*n* > 3, 1000 cells per well, 2 ways ANOVA, Tukey's multiple comparison's test * *P* < 0.05, ** *P* < 0.01, *** *P* < 0.001, **** *P* < 0.0001). (I) Immunofluorescence images of YAP/TAZ for mock and si*DOCK5* transfected cells treated with DMSO (black), 5 mM LiCl (pink) or 2 μM final Uprosertib quantified in (H). Imaged at 20×. Scale bar, 50 μm.

To investigate how depletion of DOCK5 may affect cell shape, and YAP/TAZ localisation, we performed mass spectrometry to determine total protein levels in mock and siDOCK5 transfected LM2, MDA-MB-231, and hs578t cells. Protein samples were processed 48 hours after transfection and labelled with TMT-10plex (Tandem Mass Tag) multiplex reagents before being submitted to LC–MS/MS (liquid chromatography mass spectrometry; Methods). Protein abundance values obtained after analysis of mass spectra, scaled within cell lines, and the log 2 ratio values of si*DOCK5 versus* mock-transfected lines were calculated. DOCK5 protein was depleted by approximately 78% by siRNA (Fig. 6A and Table S3, ESI†). Further there were no changes in protein levels of DOCK5's closest homolog, DOCK1^86^ (Fig. 6B), suggesting that the DOCK5 siRNA is not targeting DOCK1. In addition, there were no changes in the protein levels of RhoGEFs which clustered with DOCK5 by their QMS. Thus, DOCK5 siRNA has specific on-target effects. While LM2, MDA-MB-231, and hs578t could be distinguished by their proteome, DOCK5 depletion had similar effects on polarity proteins across cell lines (Fig. 6A, C and Table S3, ESI†).

Protein levels of connective tissue growth factor (CTGF), which are upregulated by YAP/TAZ-TEAD activity^15,87^ were significantly downregulated in DOCK5 depleted cells (log 2 ratio: −0.77695) (Fig. 6A and Table S3, ESI†); supporting the idea that depleting DOCK5 renders YAP/TAZ cytoplasmic and decreases YAP/TAZ transcriptional activity. Notably, total YAP1 and TAZ (encoded by the *WWTR1* gene) protein levels remained unchanged (Fig. 6C), matching our immunofluorescence data and confirming that DOCK5 regulates YAP/TAZ localisation rather than YAP/TAZ levels. In addition to CTGF, the protein levels of 10 established YAP/TAZ target genes^87^ were also significantly downregulated following DOCK5 knockdown in LM2 and MDA-MB-231 (Fig. 6D and Table S3, ESI†).

We performed hierarchical clustering on the abundance values of proteins which were significantly upregulated (40% increase) or downregulated (40% decrease) upon DOCK5 depletion (log 2 ratios above 0.5 and below −0.5 and permutation FDR < 0.05 (false discovery rate) (*t*-test)). DOCK5 depletion resulted in changes in the levels of AKT kinase effectors, AKT regulators, and proteins implicated in the establishment of front-rear polarity. Specifically, the protein levels of the AKT effectors GSK3β (log 2 ratio: −0.58753) and FOXO3A (log 2 ratio: −0.59131) were downregulated (Fig. 6A and C). GSK3β plays a role in the establishment of polarity where it has been described to be phosphorylated and inhibited at the leading edge of migrating cells in a mechanism dependent on integrin mediated activation of CDC42-PAR6-PKCζ.^83,88,89^ Phosphorylated GSK3β promotes polarity by capturing/stabilizing microtubules (MTs) at the leading edge of migrating cells. Interestingly although it did not make the log 2 ratio cut-off of −0.5 with a −0.46 log 2 value, levels of the polarity regulator PAR6b were also substantially decreased (27%) in DOCK5 depleted cells (*p* = 0.0002, *q* = 0.0008). Finally, PHLPP2 which inhibits AKT^90^ and aPKCζ,^91^ was upregulated (log 2 ratio: 0.855787) (Fig. 6A and Table S3, ESI†). PHLPP2 upregulation has been shown to inhibit the establishment of polarity.^91^ Thus decreased levels of polarity proteins broadly correlated with the cellular unpolarized morphology of DOCK5 depleted cells.

We used quantitative single-cell imaging approaches to quantify the subcellular localisation of GSK3β at the tips of cell protrusions (Fig. 6D). DOCK5 depleted cells had significantly reduced levels of GSK3β at the edge of protrusions compared to control cells (Fig. 6E). Total GSK3β levels were also reduced by C21 treatment, demonstrating that DOCK5's exchange activity on RAC-type GTPases is essential to maintain GSK3β levels in polarised protrusions. We propose that the inability of DOCK5 depleted cells to recruit GSK3β to the membrane leads to defects in the maintenance of polarity.

DOCK5 has been shown in osteoclasts to promote serine 9 (Ser9) phosphorylation downstream of AKT activation in both RAC dependent and independent ways that lead to MT stabilisation.^92,93^ To investigate whether DOCK5 similarly regulates the levels of phosphorylated GSK3β at the leading edge of LM2 cells to promote MT stability and the maintenance of cell polarity, we quantified the levels of phosphorylated GSK3β in LM2 cells at membrane regions following DOCK5 depletion and/or treatment with the AKT inhibitor Uprosertib (final concentration 2 μM). We confirmed that Uprosertib indeed inhibits AKT by measuring FOXO3A nuclear translocation (Fig. S6A and B, ESI†).^94,95^ We also used LiCl (final concentration 5 mM) as a positive control of our ability to measure GSK3β phosphorylation (pGSK3β). As expected, LiCl caused an increase in pGSK3β levels in protrusions in mock-transfected cells due to disruption of the negative feedback on AKT-mediated Ser9 phosphorylation by GSK3β kinase activity.^96^ Moreover, LiCl led to a significant increase in whole cell β-catenin levels *via* immunofluorescence (Fig. S6C and D, ESI†).^97–100^

DOCK5 depleted cells significantly decreased pGSK3β at the leading edge,^101^ while AKT inhibition resulted in an even greater decrease in pGSK3β levels at the protrusive edges in both mock and si*DOCK5* transfected cells (Fig. 6F and G). Strikingly, LiCl treatment rescued the phenotype of pGSK3β levels in DOCK5 depleted cells resulting in the same levels of pGSK3β in the protrusions of LiCl treated si*DOCK5* and mock transfected cells (Fig. 6F and G). The ability of LiCl to restore pGSK3β levels in DOCK5 depleted cells is likely because LiCl blocks GSK3β's ability to inhibit AKT mediated phosphorylation of Ser 9 independently of DOCK5.^96^

Taken together these data support a mechanism by which DOCK5 acts as a RAC GEF to promote phosphorylation of GSK3β at the leading edge by AKT and other kinases. Combined with prior observations that DOCK5 acts to recruit AKT to different subcellular milieus,^92,93^ we propose that recruited AKT is likely activated downstream of RAC to promote GSK3β Ser9 phosphorylation. We propose that in the absence of DOCK5 mediated phosphorylation, GSK3β and other polarity proteins become destabilized.

To test whether YAP/TAZ translocation to the cytoplasm could be due to decreases in the levels of phosphorylated GSK3β at the membrane, and decreased AKT signalling, we quantified YAP/TAZ translocation following inhibition of AKT or GSK3β kinase activity, in both mock and si*DOCK5* treated cells. Inhibiting AKT directly with Uprosertib resulted in the same decrease in YAP/TAZ ratio in mock transfected cells similar to depleting DOCK5 by RNAi (Fig. 6H and I). Furthermore, treating DOCK5 depleted cells with Uprosertib decreased the ratio significantly compared to mock transfected cells (Fig. 6H and I). LiCl however had no effect on YAP/TAZ. Elevating pGSK3β levels in control cells at the membrane alone does not further increase YAP/TAZ translocation. Hence, we propose that YAP/TAZ translocation is regulated by AKT in LM2 cells.

### DOCK5 is required for the maintenance of polarity during wound healing

**Fig. 7:**
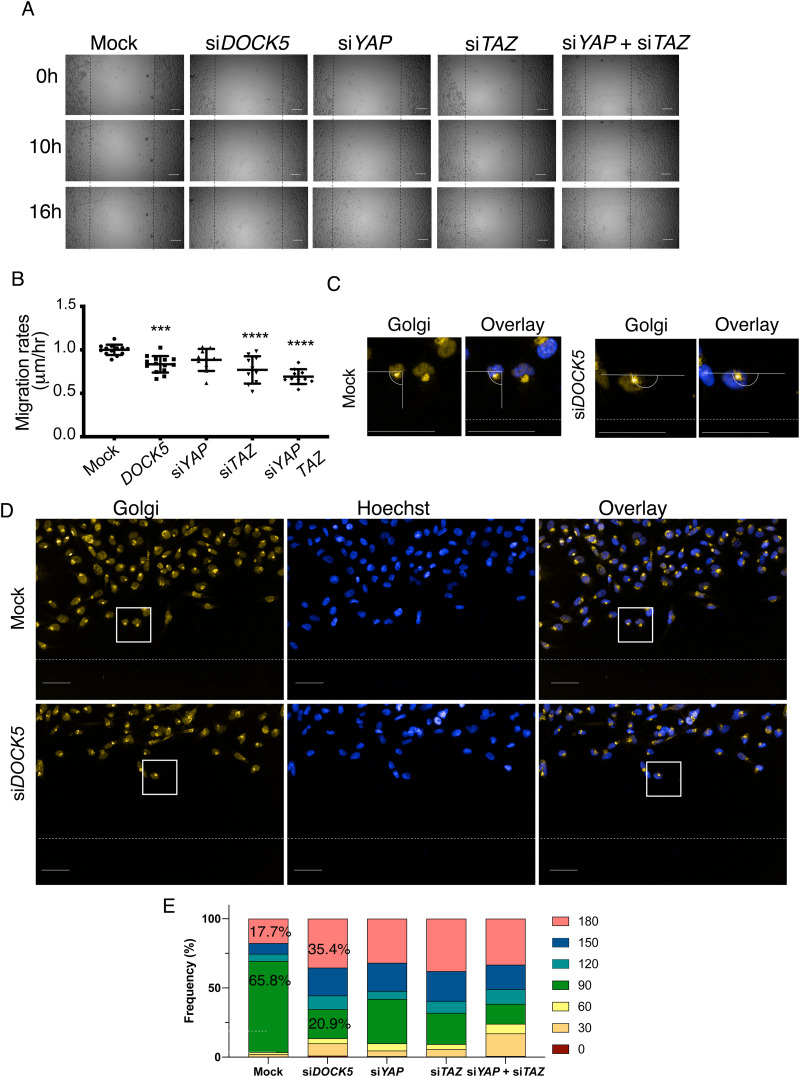
DOCK5 is required for the maintenance of polarity during wound healing. (A) Brightfield images at 0, 10 and 16 hours post creating scratch wound for mock, si*DOCK5*, si*YAP*, si*TAZ*, and si*YAP* + si*TAZ* transfected cells. Dotted black line indicates the starting line from where cells migrated over time. Scale bar, 100 μm. (B) Graph shows migration rates (μm/hour) (*n* = 5 wells per condition, with at least 2 biological repeats). Values were normalised to average of mock for each biological repeat. Mean ± sd, one-way ANOVA to mock. *** *P* < 0.001, **** *P* < 0.0001 (C) and (D) images depicting Golgi immunofluorescence stain and Hoechst, wound is shown with white dotted line. (C) Angle measuring relative to wound is depicted, example of 90 degree angle and orientation towards the wound. Scale bar is 50 μm. (E) Percentage frequency of cells with Golgis orientated 0, 30, 60, 90, 120, 150, and 180 degrees relative to the wound depicted as a bar chart. Percentages for mock and si*DOCK5* transfected cells at 90 and 180 degrees indicated.

Given the significant reduction of GSK3β following DOCK5 depletion, and the role of GSK3β in front-rear polarity,^83,89^ we hypothesised that DOCK5 contributes to the establishment of front-rear polarity in migrating cells. To test this, we performed wound healing assays, which are tests of polarity initiation and maintenance, 2D migration speed, and migration persistence (Methods). We also examined whether YAP/TAZ activation may be important for polarity establishment and migration. In these assays, we depleted DOCK5, YAP1, TAZ, and YAP1 and TAZ in combination. Depleting DOCK5, TAZ, and YAP1 + TAZ significantly reduced migration rates but depleting YAP1 did not (Fig. 7A and B). Therefore, TAZ appears to be driving migration, as well as invasion (Fig. 3C) in LM2 cells, instead of YAP1.

The establishment and maintenance of polarity during wound healing is characterised by the reorientation of the Golgi, and microtubule organising centre (MTOC), toward the wound area and direction of migration.^101^ Therefore to determine if DOCK5 contributes to the initiation and/or establishment of leading edge polarity, we quantified the orientation of the Golgi following wounding, in cells at the edge of the wound.^88,102^ Specifically, we quantified the angle of the Golgi oriented to the wound such that a 90 degree angle indicates the Golgi is facing toward the wound and front-rear polarity has been established (Fig. 7C and D). Mock transfected populations had a high frequency of cells with Golgi directly facing the wound (65.8%) (Fig. 7E). In contrast, DOCK5 depleted populations had a higher frequency of Golgi oriented parallel to the wound (180 degrees) and/or that were poorly polarised (35.4% to 17.7% respectively) (Fig. 7E). YAP1 and TAZ combined depletion resulted in the condition with the least percentage of cells displaying front-rear polarity (with Golgi oriented 90 degrees to the wound) and the highest percentage of cells with Golgi oriented 30 degrees relative to the wound, suggesting these cells are unable to integrate cues from the microenvironment for directional motility (Fig. 7E). Taken together, these data support the idea that DOCK5 promotes the formation of polarised leading-edge protrusions. In addition, because combined inhibition of YAP/TAZ is likely to have deleterious effects on various aspects of cell behaviour, such as cytoskeletal reorganisation required for polarity, the data suggest that YAP/TAZ can be both regulated by DOCK5-mediated polarisation, and also regulate polarisation itself.

### DOCK5 promotes the maintenance of polarity and alters FA dynamics.

**Fig. 8:**
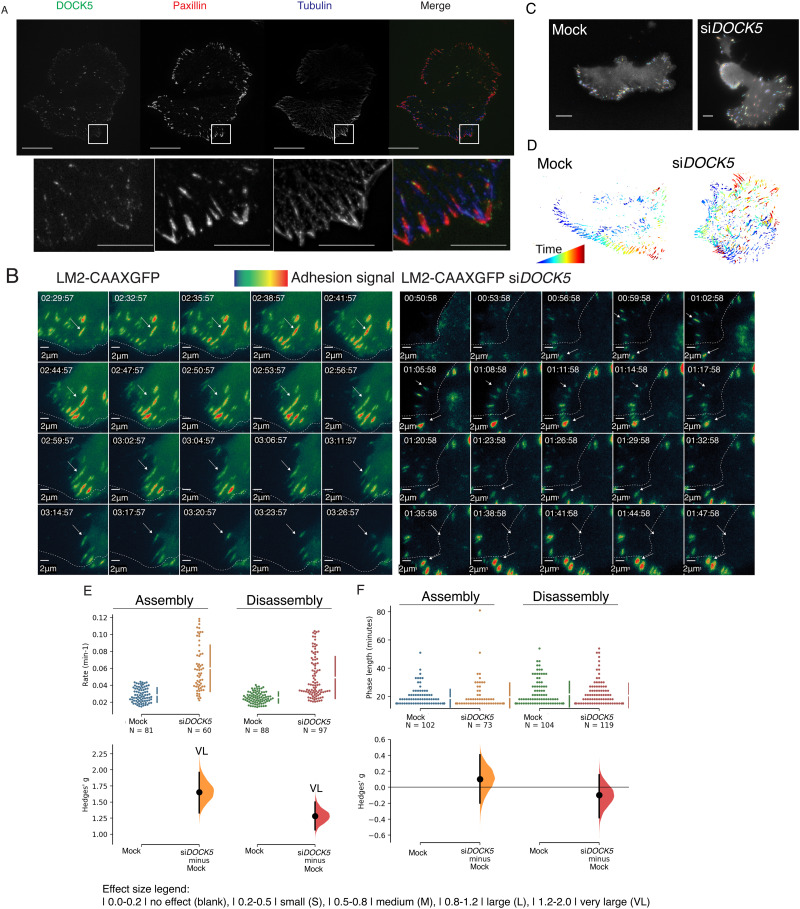
DOCK5 promotes the maintenance of polarity and alters FA dynamics. (A) Overexpressed DOCK5-tagged YFP localises to focal adhesions (paxillin). Cells stained for paxillin (red), tubulin (blue). Scale bar is 5 μm. (B) Sequences of zoomed in sections of mock, and si*DOCK5* transfected LM2-CAAXGFP cells over time. Frames are every 3 minutes. Arrows indicate an example of an adhesion to follow. Dotted line represents the edge of the cell at the beginning of each sequence of frames shown. Adhesion signal represents Talin mApple intensity: red more intense, blue less intense. Scale bar is 2 μm. (C) Representative images of how Focal Adhesion Analysis Server (FAAS)^104,105^ picks up adhesions on mock and si*DOCK5* transfected cells. Scale bar is 10 μm. (D) Focal adhesions tracked over time in same cells as in (C). Blue to red indicates adhesions which have been present for less, and longer amounts of time respectively. (E) and (F) Cumming estimation plots of Hedge's *g* for comparison between (E) assembly and disassembly rates, and (F) assembly phase length and disassembly phase length of adhesions in mock *versus* si*DOCK5* transfected cells. *N* shows number of adhesions quantified, data from 4 cells. Raw data is plotted in the top axes, while mean difference is plotted in lower axes as a bootstrap sampling distribution. Mean differences are shown as dots, 95% confidence intervals are shown by the ends of the vertical error bars. Generated using Ho *et al.*, 2019^106^ website. Effect size legend |0.0–0.2| no effect (blank), |0.2–0.5| small (S), |0.5–0.8| medium (M), |0.8–1.2| large (L), |1.2–2.0| very large (VL). (E) Unpaired Hedge's *g* between mock and si*DOCK5* for assembly and disassembly rates are 1.65 (VL) (95% CI 1.33, 1.96), and 1.28 (VL) (95% CI 1.07, 1.50) respectively. Two-sided *P* values of Mann–Whitney tests are 3.64 × 10^−15^, and 1.04 × 10^−15^ for assembly and disassembly rates respectively. (F) Unpaired Hedge's *g* between mock and si*DOCK5* for assembly phase length and disassembly phase length are 0.101 (95% CI −0.199, 0.405), and −0.00986 (95% CI −0.38, 0.155) respectively. Two-sided *P* values of Mann–Whitney tests are 0.602, and 0.437 for assembly and disassembly phase lengths respectively. *N* number is individual adhesions from 4 cells, filtered by them being present for a minimum of 5 frames (phase length 5) and mean axial ratio less than 3. Unpaired Mann–Whitney test, *P* < 0.001.

We and others have demonstrated that DOCK5 localises in close proximity to FAs peripherally to Paxillin, but co-localises with Paxillin in the absence of GIT2.^69,103^ In line with these data, we observed that overexpressed DOCK5-YFP is also localised in puncta at the very edge of the lamellipodial protrusions in TNBC cells. DOCK5 bordered the forward-facing side of Paxillin-positive FAs at the leading edge (Fig. 8A). Notably, the rear facing side of Paxillin-positive FAs at the leading edge was often bordered by polymerised alpha-tubulin (Fig. 8A).

To determine if DOCK5 has a role in FA dynamics in LM2 cells which may explain the polarity defects, we used total internal reflection fluorescence (TIRF) microscopy to visualise the recruitment of Talin-mApple to FAs in migrating mock and DOCK5 depleted cells. In migrating mock transfected LM2 cells, small Talin-positive FAs were established 0–4 μm from the leading edge in the lamellipodia of migrating cells (Fig. 8B and C, adhesions coloured by FAAS software; Methods, Movie S1, ESI†). These FAs then enlarged/matured and turned over in the same region of attachment to the ECM, and FAs ere not stable in the lamella and/or cell body as the cell moved over this region (Fig. 8D – dynamics, Movie S1, ESI†).

While migrating mock cells established, and maintained, a polarised leading edge which is the site of both FA formation and turnover (Fig. 8C and Movie S1, ESI†), the changes in FA dynamics in DOCK5 depleted cells coincided with a failure of these cells to maintain polarity (Fig. 8D and Movie S2, ESI†). Once established, the leading edge in randomly migrating control cells was maintained for approximately 120 min. In DOCK5 depleted cells there was rarely only one leading edge at any given time but rather many protrusions lasting approximately 40 minutes on average (Movie S2, ESI†). Thus DOCK5 depleted cells were capable of initial establishing polarised protrusions, but the protrusions were not stable. These data demonstrate that DOCK5 depleted cells initiated the formation of a polarised leading edge but fail to maintain it.

We quantified the assembly and disassembly dynamics of individual FAs in mock and DOCK5 depleted cells. We used a Cumming estimation plot to demonstrate the mean difference in the assembly and disassembly rates and phase lengths. Effect sizes based on Cohen's initial suggestions^107,108^ indicate that values of 0.2 to 0.5 are small, 0.5 to 0.8 are medium, 0.8 to 1.2 are large, and 1.2 to 2.0 are very large effect sizes. In DOCK5 depleted cells, FAs have significantly accelerated assembly and disassembly rates compared to control FAs (Fig. 8E, orange unpaired Hedges’ *g* of 1.65; red unpaired Hedges’ *g* of 1.28). But disassembly phase lengths were largely unaffected (Fig. 8F, Hedge's *g* of 0.101 and −0.0986 for assembly and disassembly phase lengths respectively). Despite accelerated disassembly rates, FAs in DOCK5 depleted cells often failed to completely turnover (Fig. 8B–D) and DOCK5 depleted cells shift their mass over FA structures that have not turned-over completely as they migrate such that FAs appear to be near the nucleus (Fig. 8D and Movie S2, ESI†).

The most parsimonious explanation for these observations is that in the absence of DOCK5, nascent FAs can form, but are also highly unstable and exhibit fast shrinkage rates, potentially because actin-mediated force maturation is also impaired in DOCK5 depleted cells.^109^ Meaning, FAs in DOCK5 depleted cells are poorly coupled to RHOA-dependent actomyosin activity and do not mature properly. However, the absence of actomyosin engagement at FAs in DOCK5 depleted cells also means that turnover of existing FA structures is also impaired, and thus FAs are “left behind” as the cell moves over them (Fig. 8D). This model is consistent with the observation that lamellipodia are highly unstable in DOCK5 depleted cells (Movie S2, ESI†).

### CDC42 and RHOA depletion affect FA morphogenesis in metastatic cells

**Fig. 9:**
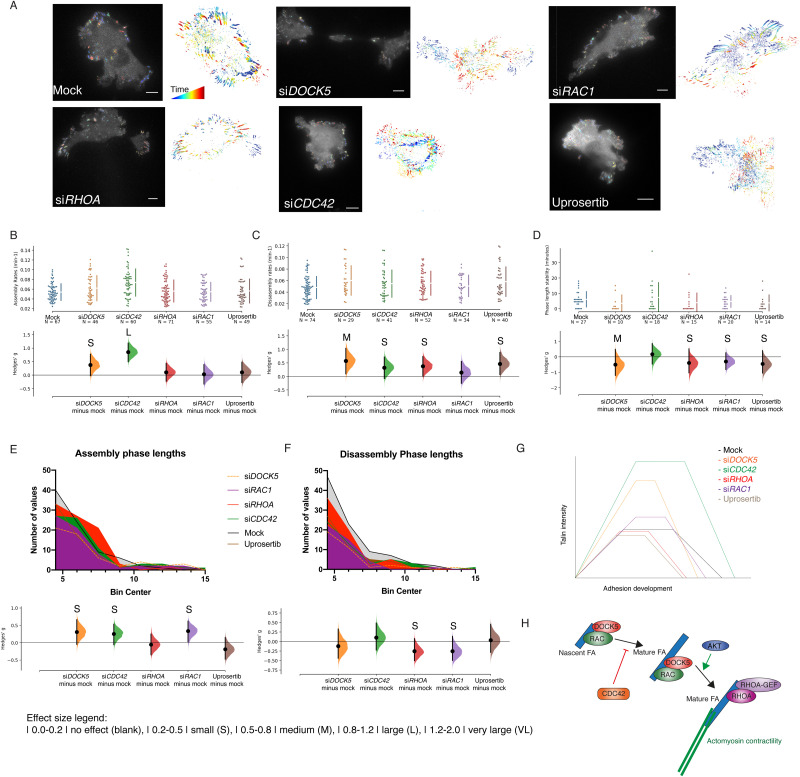
CDC42 and RHOA depletion affect FA morphogenesis in metastatic cells. (A) Representative images of LM2-CAAXGFP cells transfected with Talin mApple and si*DOCK5*, si*CDC42*, si*RHOA*, si*RAC1*, mock, and treated with the AKT inhibitor Uprosertib. Left and right panels per condition show FAAS picking up the adhesions and adhesions tracked over time for the same cell. (B)–(D) Cumming estimation plots of Hedge's *g* for comparison between (B) assembly and (C) disassembly rates, as well as (D) stability phase length of adhesions in mock *versus* si*DOCK5*, si*CDC42*, si*RHOA*, si*RAC1* transfected cells, and Uprosertib treated cells. *n* shows number of adhesions. Data is taken from 3 cells per condition. Raw data is plotted in the upper axes, while mean difference is plotted in lower axes as a bootstrap sampling distribution. Mean differences are shown as dots, 95% confidence intervals are shown by the ends of the vertical error bars. Generated using Ho *et al.*, 2019^106^ website. Effect size legend |0.0–0.2| no effect (blank), |0.2–0.5| small (S), |0.5–0.8| medium (M), |0.8–1.2| large (L), |1.2–2.0| very large (VL). (B) Unpaired Hedge's *g* for assembly rates between mock and conditions: si*DOCK5*: 0.375 (S) (95% CI −0.0214, 0.776); si*CDC42*: 0.851 (L) (95% CI −0.499, 1.19); si*RHOA*: 0.106 (95% CI −0.229, 0.436); si*RAC1*: 0.0311 (95% CI −0.332, 0.387); Uprosertib: 0.105 (95% CI −0.276, 0.479). Two-sided *P* values of Mann–Whitney tests are 0.242, 2.090 × 10^−5^, 0.922, 0.963, 0.808 respectively. (C) Unpaired Hedge's *g* for disassembly rates between mock and conditions: si*DOCK5*: 0.574 (M) (95% CI 0.0979, 1.04); si*CDC42*: 0.321 (S) (95% CI −0.0767, 0.715); si*RHOA*: 0.376 (S) (95% CI 0.0138, 0.732); si*RAC1*: 0.145 (95% CI −0.252, 0.558); Uprosertib: 0.467 (S) (95% CI 0.0641, 0.887). Two-sided *P* values of Mann–Whitney tests are 0.0363, 0.1840, 0.0580, 0.4030, 0.0652 respectively. (D) Unpaired Hedge's *g* for stability phase lengths between mock and conditions: si*DOCK5*: −0.507 (M) (95% CI −1.20, 0.494); si*CDC42*: 0.167 (95% CI −0.464, 0.856); si*RHOA*: −0.395 (S) (95% CI −1.03, 0.517); si*RAC1*: −0.299 (S) (95% CI −0.827, 0.259); Uprosertib: −0.456 (S) (95% CI −1.04, 0.393). Two-sided *P* values of Mann–Whitney tests are 0.0602, 0.6390, 0.06080, 0.4590, 0.0476 respectively. (E) Histogram of assembly phase lengths for all conditions (top) and unpaired Hedge's *g* for assembly rate phase lengths between mock and conditions (bottom): si*DOCK5*: 0.307 (S) (95% CI −0.0508, 0.662); si*CDC42*: 0.256 (S) (95% CI −0.0605, 0.536); si*RHOA*: −0.0537 (95% CI −0.35, 0.247); si*RAC1*: 0.336 (S) (95% CI 0.0297, 0.621); Uprosertib: −0.187 (95% CI −0.471, 0.148). Two-sided *P* values of Mann–Whitney tests are 0.140, 0.154, 0.471, 0.237, 0.390 respectively. Mock *n* = 83, si*DOCK5 n* = 56, si*CDC42 n* = 74, si*RHOA n* = 83, si*RAC1 n* = 67, Uprosertib *n* = 61. (F) Histograms of disassembly phase lengths (top) and unpaired Hedge's *g* for disassembly rate phase lengths between mock and conditions (bottom): si*DOCK5*: −0.12 (95% CI −0.439, 0.39); si*CDC42*: 0.106 (95% CI −0.224, 0.483); si*RHOA*: −0.255 (S) (95% CI −0.505, 0.0726); si*RAC1*: −0.251 (S) (95% CI −0.494, 0.14); Uprosertib: 0.037 (95% CI −0.285, 0.456). Two-sided *P* values of Mann–Whitney tests are 0.550, 0.633, 0.270, 0.435, 0.818 respectively. Mock *n* = 92, si*DOCK5 n* = 35, si*CDC42 n* = 50, si*RHOA n* = 64, si*RAC1 n* = 42, Uprosertib *n* = 50. (G) Summary of results and how each siRNA and treatment is affecting adhesion life cycle. Not to scale. (H) Cartoon summarising our data where CDC42 inhibits FA growth and turnover, RAC1 promotes FA growth, and RHOA and AKT promote engagement of FAs with actomyosin leading to FA maturation (stability) as well as normal turnover.

We investigated whether the defects in FA morphogenesis following DOCK5 depletion could be attributed to decreased DOCK5-mediated RAC activation, or whether DOCK5 regulates FA morphogenesis in RAC-independent ways. We first knocked down RAC1, RHOA, and CDC42 and measured Talin-mApple dynamics in migrating LM2 cells (Fig. 9A). We also measured FA dynamics following treatment with Uprosertib to determine the effect of AKT signalling on FA morphogenesis.

Depleting CDC42 significantly accelerated assembly rates (Hedges’ *g*: 0.851) in a similar manner to DOCK5 depletion, and large FAs formed rapidly (Fig. 9B) (Movie S3, ESI†). However, all other aspects of FA morphogenesis including disassembly rates (Fig. 9C), stability (Fig. 9D), the length of assembly (Fig. 9E), and disassembly phase length (Fig. 9F) were largely unaffected.

In contrast, RAC1 depletion increased the length of the assembly phase (Fig. 9E); consistent with the role of RAC1 in promoting FA assembly^110^ (Movie S4, ESI†). RHOA depletion resulted in a significant increase in FA disassembly rates (Fig. 9C), in line with the idea that in the absence of RHOA-mediated actomyosin engagement, FAs fail to mature (Movie S5, ESI†). Uprosertib treatment resulted in similar effects as RHOA inhibition, as FAs had high disassembly rates, indicative of unstable adhesions (Fig. 9C) (Movie S6, ESI†). However, the effect on FA morphogenesis based on all other metrics following depletion of RAC1 and RHOA, or following Uprosertib treatment, was relatively mild.

Taken together these data suggest that CDC42 inhibits FA growth and turnover, RAC1 promotes FA growth, while RHOA and AKT promote engagement of FAs with actomyosin leading to FA maturation (stability) as well as normal turnover (Fig. 9H). The phenotype observed following DOCK5 depletion encompasses all those observed following depletion of Rho GTPases. This suggests that DOCK5 has much broader effects on FA morphogenesis than any single Rho GTPase alone.

### DOCK5 and CDC42 stabilise polarity

**Fig. 10:**
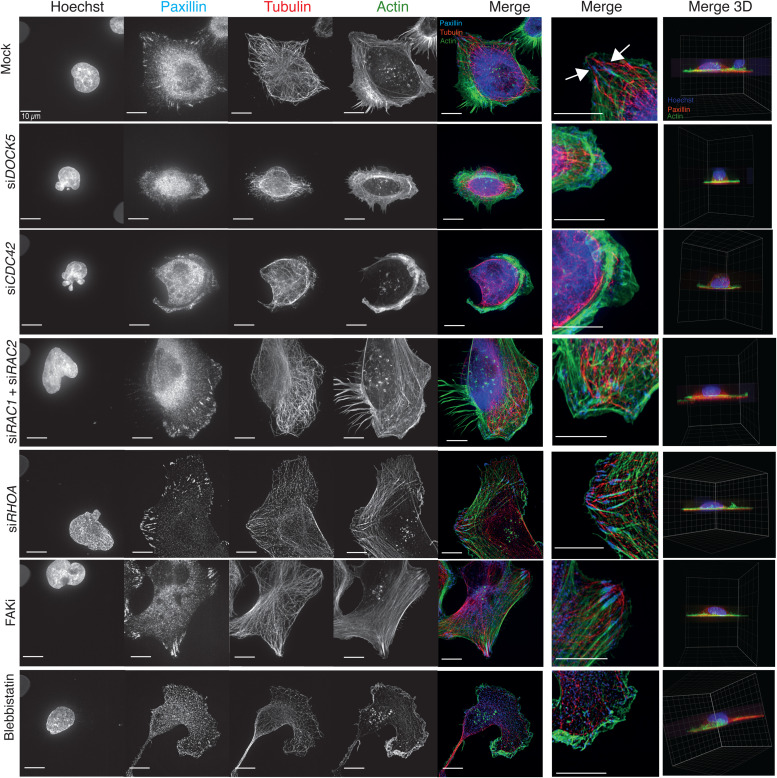
DOCK5 and CDC42 stabilise polarity. Images taken on SoRA for mock, si*DOCK5*, si*CDC42*, si*RAC1+* si*RAC2*, si*RHOA*, and treated with FAKi, and Blebbistatin. Stained for DNA, Paxillin, α-tubulin, F-actin. Merge 2D projection and zoomed section: Paxillin (blue), α-tubulin (red), F-actin (green). Merge 3D projection: Paxillin (red), DNA (blue), F-actin (green). Scale bar = 10 μm.

We next sought to explore the idea that DOCK5 acts to modulate a CDC42-GSK3β axis that stabilises MTs at the leading edge of protrusive cells to reinforce the establishment of polarity. Cells were plated on fibronectin (FN) and fixed after 3 hours. After 3 hours, spreading LM2 cells establish a single leading edge adhered to the matrix by stable FAs, and thus have an asymmetric morphology (Fig. 10). In regions with large FAs, MTs were typically extended into the protrusions (Fig. 10 zoomed insert, arrows) supporting the idea that MTs are captured at the cortex in regions with active FA morphogenesis.

After 3 hours of spreading, DOCK5 depleted cells established multiple smaller protrusions, leading to asymmetric shapes (Fig. 10). Paxillin-positive FAs in DOCK5 depleted cells were very small and appeared diffusely spread in the leading edge (Fig. 10 zoomed insert). Consistent with previous reports demonstrating a role of DOCK5 in promoting MT stability,^92,93^ there were dramatically fewer MT filaments in the periphery of DOCK5 depleted cells, and MTs appear thin and poorly organized compared to those of mock transfected cells. In DOCK5 depleted cells MTs appear organised largely as bundles around the nucleus. MT bundles are often hallmarks of stabilised, old MTs that exhibit low instability and are not captured at the cortex.^111,112^ Thus we conclude DOCK5 is essential for MT capture at the cortex in protrusions.

CDC42 depletion led to a partially similar phenotype as that of DOCK5, as cells formed expanded lamellipodia that nearly encompassed the entire circumference of the cells, and adopted a rounded, symmetric shape (Fig. 10). However, Paxillin-positive FAs in CDC42 depleted cells appeared globular and poorly formed – consistent with live cell imaging data suggesting CDC42 inhibits FA assembly (Fig. 10). In contrast, cells depleted of RHOA, or RAC1 + RAC2 all formed polarised lamellipodia with multiple large FAs (Fig. 10), hallmarks of defective FA maturation and/or turnover, but not in MT organization or establishment of polarity. Treatment of cells with either a focal adhesion kinase inhibitor (FAKi), which prevents focal adhesion turnover; or Blebbistatin, a contractility inhibitor, did not inhibit the formation of polarised lammelipodia, although no FAs formed in Blebbistatin treated cells (Fig. 10). We conclude DOCK5 promotes the stability of polarised leading edge by acting at FAs to promote CDC42-mediated MT capture at the cortex.

### DOCK5 promotes spreading following MT depolymerisation

**Fig. 11:**
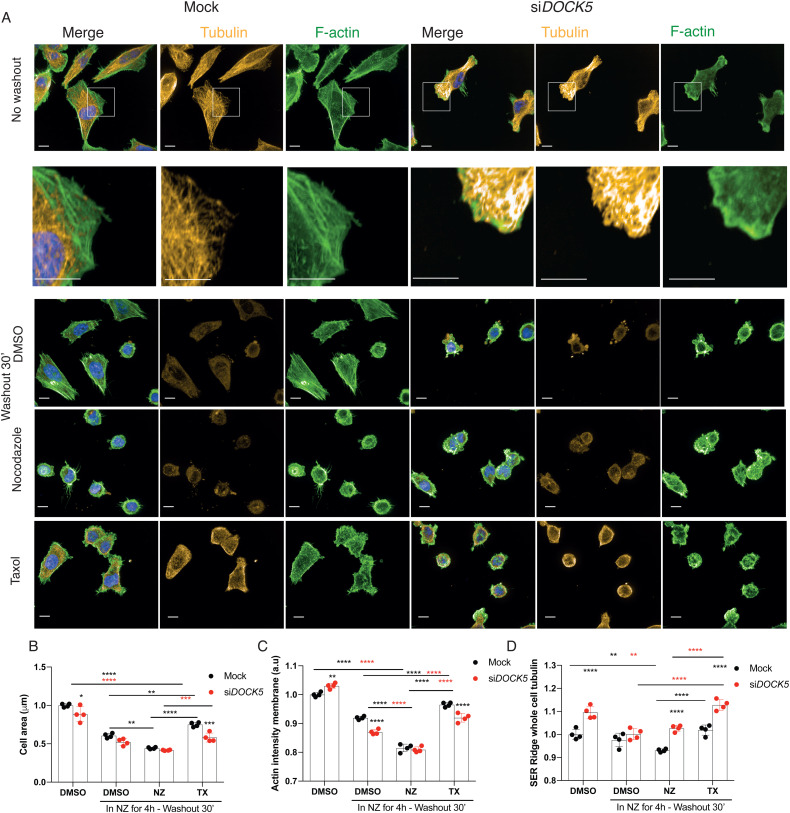
DOCK5 promotes spreading following MT depolymerisation. (A) Immunofluorescence images (maximum projections) of mock or si*DOCK5* transfected cells treated just with DMSO or treated with nocodazole (NZ) for 4 hours and then washed out in either media containing DMSO, NZ, or taxol (TX). Stained for DNA (blue), tubulin (yellow), and F-actin (green). Scale bar is 10 μm. Imaged at 60× with water objective (NA = 1.2). Images shown with same intensity settings. White box represents zoomed in sections below. (B)–(D) Quantifications of (A) showing (B) cell area, (C) actin intensity at the membrane, and (D) whole cell SER Ridge tubulin (texture). Mean per well ± sd are shown (*n* > 3 wells, 1000 cells per well, 2 ways ANOVA, Tukey's multiple comparison's test * *P* < 0.05, ** *P* < 0.01, *** *P* < 0.001, **** *P* < 0.0001). Significance between Mock and si*DOCK5* for each condition shown with no bars. Significance between mock NZ (black) and si*DOCK5* NZ (red) compared to other treatments for same transfection.

DOCK5 has been shown to promote MT stability downstream of GSK3β phosphorylation.^92,93^ To investigate the relationship between MTs and DOCK5 at the leading edge, we performed assays to analyse cell morphology following washout of the MT-depolymerization agent nocodazole (NZ) in media containing either: NZ, the MT-stabilising agent taxol (TX) or DMSO. Following 4 hours of NZ both mock and DOCK5 depleted cells showed decreased spread area, adopted a rounded morphology and actin intensity at the membrane was significantly decreased (Fig. 11A–C). However, while in control cells MT staining was expectedly diffuse, in DOCK5 depleted cells tubulin was localised into large high-intensity “bundles” which was reflected by significant increases in the SER Ridge Feature (texture feature) (Fig. 11D). These data show that MTs are differentially affected by MT depolymerising agents in control vs DOCK5 depleted cells, and we propose that depletion of DOCK5 leads to MT bundling and stability because MTs are not captured at the cell cortex.^111,112^

Thirty minutes after washout in DMSO containing media, control cells formed abundant filopodial protrusions and increased in cell area (Fig. 11A and B, *p* = 0.013) likely as a result of MT outgrowth and RAC1 promoting actin polymerisation.^113,114^ Indeed, actin intensity at the membrane significantly increased in DMSO media washout denoting actin polymerisation (Fig. 11C). DOCK5 depleted cells also spread and increased in cell area under DMSO containing media washout (Fig. 11B, *p* = 0.013 for mock, *p* = 0.0894 for si*DOCK5*) but had lowered levels of actin at the membrane (Fig. 11C) – supporting the idea that DOCK5 is required for the stability of actin-based protrusions. DOCK5 depleted cells did not display polarised fronts for any condition supporting our hypothesis that DOCK5 is required for maintenance of polarity. Notably washout of NZ in DOCK5 depleted cells affected MT organisation in an identical manner as control – effectively rescuing the effects of DOCK5 depletion on MT organisation following NZ treatment. Therefore, it is unlikely DOCK5 regulates polymerisation of MTs during spreading or migration, but rather regulates MT capture.

Washout in TX containing media resulted in a significant increase in cell area for both mock and DOCK5 depleted cells compared to washout in DMSO alone, effectively accelerating the rate of spreading, though the ability of TX to rescue spreading was blunted in DOCK5 depleted cells (Fig. 11A and B, *p* = 0.005 for mock, *p* = 0.9114 for si*DOCK5*). Accelerated spreading was likely due to increased actin polymerisation, as both mock and DOCK5 depleted cells in TX had increased levels of actin at the membrane compared to DMSO washout alone, and were at levels comparable to controls. Strikingly however, TX resulted in significant increase of MT bundles in DOCK5 treated cells, that were even higher than the DMSO treated DOCK5 depleted cells (Fig. 11D). We propose that the formation of MT bundles in TX washout is due to accelerated MT polymerisation due to TX, but a failure to capture MT plus ends at the leading edge.

## Discussion

In both yeast and mammalian cells the recruitment and activation of CDC42 at the membrane serves to seed polarised cortical structures that are enriched complexes and cytoskeletal elements that would otherwise be diffusely or asymmetrically organised in cells.^115^ During the formation of the bud in *Saccharomyces cerevisiae*, localised CDC42 activation marks the site of bud assembly at one site on the membrane.^116,117^ A key aspect of bud morphogenesis are positive feedback loops initiated by CDC42 which increase the local concentration of GTP-bound and active CDC42,^118^ resulting in one bud site outcompeting all other potential sites for the recruitment of bud assembly.^119,120^ Indeed in yeast mutants where the reinforcement, but not necessarily the establishment, of polarity is disrupted, multiple bud sites form.^120,121^

**Fig. 12:**
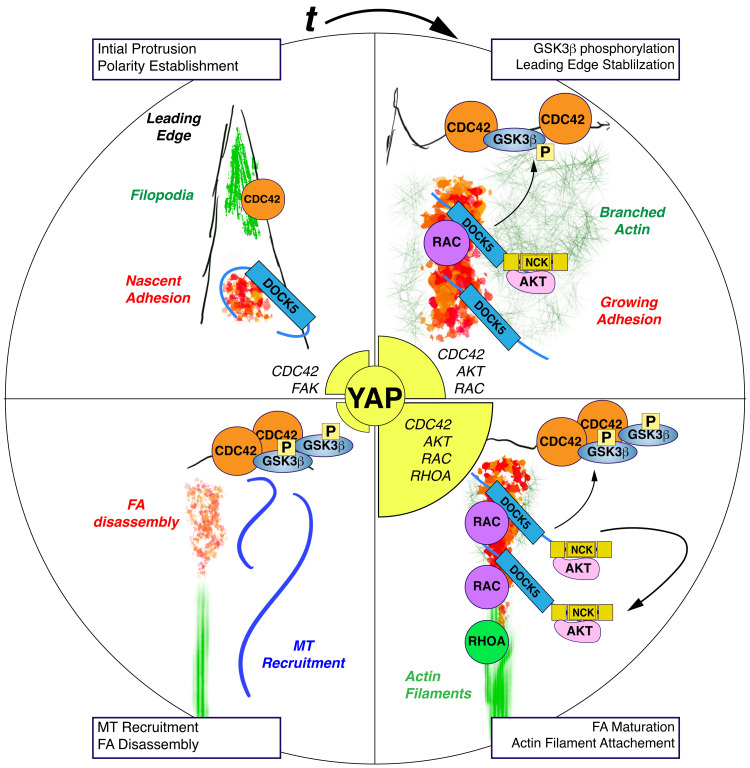
DOCK5 acts at FAs in breast cancer cells to establish positive feedback loops which maintain polarity in the presence of stable integrin-ECM attachments and cellular tension. Schematic of proposed model where in WT conditions DOCK5 gets recruited to FAs and acts as GEF for RAC-type GTPase to promote FA assembly and turnover/maturation in a positive feedback loop. At the same time DOCK5 recruits and activates AKT to FAs near the leading-edge maintaining GSK3β, which is targeted by CDC42-PKCζ for phosphorylation, leading to the maintenance of polarity. Here DOCK5 increases the levels of AKT and thus promotes further GSK3β stability and phosphorylation in another positive feedback loop. Further, actomyosin engagement by FA results in high RHOA and consequently high nuclear YAP/TAZ. Finally, DOCK5 could potentially mature FAs to recruit CDC42 GEFs which can further amplify CDC42 activation at the leading edge.

In migrating mammalian cells, the establishment of polarity occurs with the coincident reorganisation of the actomyosin cytoskeleton to generate protrusions such as lamella and lamellipodia, and the engagement of the ECM by integrins to form FAs.^122,123^ Classical studies have shown that CDC42 regulates microtubule stability and establishment of polarity *via* PAR6 and AKT/PKCζ mediated phosphorylation of GSK3β at the leading edge of migrating cells in a manner that is integrin dependent.^83,88,89^ Here we propose that DOCK5 acts at FAs in breast cancer cells to establish positive feedback loops which maintain polarity in the presence of stable integrin-ECM attachments and cellular tension. By reinforcing polarity in sub-cellular regions with stable FA attachments, DOCK5 promotes processive migration and 3D invasion (Fig. 12).

We and others have previously observed that DOCK5 is recruited to FAs,^69,103^ and here we show that DOCK5 acts to couple FA morphogenesis and polarity by coordinating multiple GTPase activities. Firstly, DOCK5 acts as a GEF for RAC-type GTPase to promote FA assembly and ultimately maturation/turnover. In the absence of DOCK5, FAs do not increase in size, and the turnover of existing FAs is disrupted (Fig. 8) likely because of a failure of DOCK5-depleted FAs to engage actin at the leading edge.^109,124,125^ Failure to mature FAs and/or engage actomyosin likely results in decreases in RHOA activation (Fig. 12). Secondly, by recruiting and activating AKT to FAs near the leading edge, DOCK5 maintains GSK3β at the leading edge, which is targeted by CDC42-PKCζ for phosphorylation, leading to the maintenance of polarity. GSK3B phosphorylation leads to stabilization of the leading edge, further CDC42 recruitment and activation, and ultimately the recruitment of MTs which will disassemble FAs. Indeed, a role for DOCK5 recruitment of MTs to the leading edge has previously been established.^92,93,126^

In cells depleted of DOCK5, CDC42 activation at the membrane and the initial establishment of polarity is initially unaffected. But either C21 treatment or lack of DOCK5 reduces the levels of GSK3β that can be phosphorylated by CDC42-PKCζ, leading to a failure of cells to maintain polarity. CDC42 activation (which is initially independent of DOCK5) at another subcellular site can lead to formation of a new unstable leading edge in other regions of the cells. Indeed, a characteristic of DOCK5 depleted cells is not the ability to form protrusions, but rather to maintain a stable polarised protrusion. As such this phenotype is highly reminiscent of *rdi1* and *bem1/2* yeast mutants which form numerous unstable buds.

DOCK5 has been previously described to recruit and activate AKT and promote GSK3β phosphorylation through both RAC-dependent and independent ways.^92,93^ In particular, DOCK5's C-terminus has been shown to recruit NCK/AKT complexes to promote GSK3β phosphorylation in ways that are independent of catalytic activation of RAC-type GTPase through its DHR2/GEF domain.^92,93^ But our observation that the DOCK5 GEF inhibitor C21 mimics the effect of both DOCK5 siRNA and AKT inhibition by Uprosertib on GSK3β localisation at the membrane, and YAP/TAZ translocation, strongly suggests that DOCK5 mediated activation of RAC-type GTPases *via* its DH2/GEF is required for GSK3β phosphorylation and localisation at the leading edge. One model which resolves these previously contradictory observations is that activated DOCK5 exists in a conformation that recruits NCK and AKT to its C-terminus independent of it GEF domain; but also functions as a GEF to promote RAC-mediated FA assembly – which recruits more DOCK5/NCK/AKT to FAs in a positive feedback loop. Thus, inhibition of RAC by C21 breaks this loop and lowers AKT activation. Our observation that AKT inhibition by Uprosertib mimics different aspects of DOCK5 depletion on FA morphogenesis in fact suggests that AKT activity itself promotes FA morphogenesis and is involved in this positive feedback loop (Fig. 12).

We and others have previously demonstrated that YAP/TAZ nuclear translocation is dependent on cell shape and geometry.^3,4,10,14,44^ our data suggest that decreases in YAP/TAZ nuclear translocation in DOCK5 depleted cells are due to the inability of DOCK5 depleted cells to maintain a polarised, adherent morphology leading to low YAP/TAZ. Mechanistically this can be explained by the inability of cells to assemble stable a polarized leading edge and mature FAs. This leads to both defects in assembly of complexes at FA which promote YAP/TAZ activation – *i.e. via* signalling pathways such as PAK2^10^ or FAK-PI3K^127^ and/or the failure of FAs in DOCK5 depleted cells to engage actin and upregulate actomyosin contractility^122,123^ which promotes YAP/TAZ activation.^128^ Indeed, that both CDC42 and RHOA depletion phenocopy DOCK5 depletion in terms of decreased nuclear YAP/TAZ, they act downstream of FAs to promote YAP/TAZ translocation (Fig. 1A, 9B and 10).

Finally, we cannot dismiss the potential changes in Hippo and/or Wnt signalling pathways that may occur following DOCK5 depletion which could alter YAP/TAZ translocation.^14,129^ Indeed, our proteomic data reveals downregulation of the Hippo component SAV, and of the Wnt signalling inhibitor DKK1 (Fig. 6C) in DOCK5 depleted cells. Moreover, the ligand Wnt7b was increased following DOCK5 depletion. As both DKK1 and SAV are YAP/TAZ targets^87^ and DKK1 has been shown to increase as a result of YAP overexpression,^130^ this suggests that their downregulation is likely a result of decreased nuclear YAP/TAZ.

It is tempting to speculate that sustained YAP/TAZ nuclear translocation, and resulting capabilities such as drug resistance, may be a consequence of cancer cells adopting stable polarised migratory forms. This model would contrast the classical idea that drug resistance and migration are coincident events, and/or that YAP/TAZ itself drives migration. Indeed, studies in melanoma cells have shown that YAP/TAZ upregulation which promotes drug resistance is caused by shape changes.^22,24,40,41^ Moreover, a number of recent studies have shown that EMT in breast cancer, which is characterized by morphological changes and increased invasive capabilities, also leads to drug resistant cells.^131^ Thus, shape changes may be causal to many aspects of TNBC, and suggests that therapeutic intervention could occur by preventing cells from altering their shape.

## Materials and methods

### Cell culture

LM2s (subpopulation 4172 from MDA-MB-231) were obtained from Joan Massagué (Sloan Kettering Institute, New York),^43^ and stably express a triple-fusion protein reporter construct encoding herpes simplex virus thymidine kinase 1, green fluorescent protein (GFP) and firefly luciferase (Minn *et al.*, 2005). MDA-MB-231 cells were obtained from Janine Erler (University of Copenhagen, Denmark), while SUM159 and hs578t were a gift from Rachel Natrajan (Institute of Cancer Research, London). Cells were grown in Dulbecco's modified Eagle medium (DMEM) supplemented with 10% heat-inactivated fetal bovine serum (FBS) and 1% penicillin/streptomycin in T75 falcons, at 37 °C and supplemented with 5% CO_2_ in humidified incubators. MCF10A were obtained from ATCC and were grown in DMEM/F12 supplemented with 5% horse serum, 10 μg ml^−1^ insulin, 20 ng ml^−1^ epidermal growth factor, 100 ng ml^−1^ cholera toxin, 500 ng ml^−1^ hydrocortisone, and 100 mg ml^−1^ penicillin/streptomyocin in T75 falcons.

T47D were obtained from from Rachel Natrajan (ICR), and were grown in Roswell Park Memorial Institute (RPM1)-1640 culture medium (Gibco) supplemented with 10% heat-inactivated fetal bovine serum (FBS) and 1% penicillin/streptomycin in T75 falcons.

### siRNA transfections

All siRNAs were made up to a stock of 20 μM. Briefly, for 384 well plates, siRNA reverse transfections were performed by adding 40 nl of siRNA to each well followed by 5 μl of Opti-MEM® Reduced Serum Media. 5 minutes later 5 μl of mix containing Opti-MEM and RNAimax reagent in a 125 : 1 ratio were added per well. Plates were spun at 1000 rpm for 1 minute, and incubated at room temperature for 20 minutes to allow siRNA-RNAimax complexes to form. Cells were plated on top in 30 μl of DMEM (10% FBS, 1% penicillin/streptomyocin), resulting in a total volume of 40 μl per well: 40 nl siRNA (final concentration of 20 nM), 5 μl Opti-MEM, 5 μl of Opti-MEM and RNAimax mix (125 : 1) plus 30 μl of cells. For transfections performed on 96 well plates and 6 well plates, 4 and 64 times the amounts used for 384 well plates were used, respectively. When siRNAs were transfected in combination, the same amount of each siRNA was used. siRNAs and sequences used can be found in Table 1.

**Table 1:** siRNA target sequences

siRNA	Sequences	Catalogue number
*DOCK5* OTP pool	GAGGAGAGAUUGUUAAGUU	# L-018931-00-0005
	GAACAUUAAACACAACCUA	
	GCAAAUUCAUUCAAAGCAU	
	GGGUUGCGAUCUAUAACUA	
*DOCK5* OTP pool deconvoluted^5–8^	GAGGAGAGAUUGUUAAGUU	# LU-018931-00-0002
	GAACAUUAAACACAACCUA	
	GCAAAUUCAUUCAAAGCAU	
	GGGUUGCGAUCUAUAACUA	
*DOCK5* siGENOME pool deconvoluted^1–4^	AGAACUAUCUAAUUCGUUG	# MU-018931-01-0002
	GUAACGGGAUGCCCAAGGA	
	GAGUGGCAGUGAUGGAUAU	
	UAUCAUACAUGGGAAGGUG	
*YAP* OTP pool	GCACCUAUCACUCUCGAGA	# L-012200-00-0005
	UGAGAACAAUGACGACCAA	
	GGUCAGAGAUACUUCUUAA	
	CCACCAAGCUAGAUAAAGA	
*WWTR1* (TAZ) OTP pool	CCGCAGGGCUCAUGAGUAU	# L-016083-00-0005
	GGACAAACACCCAUGAACA	
	AGGAACAAACGUUGACUUA	
	CCAAAUCUCGUGAUGAAUC	
*LATS1* OTP pool	GGUGAAGUCUGUCUAGCAA	#L-004632-00-0005
	UAGCAUGGAUUUCAGUAAU	
	GGUAGUUCGUCUAUAUUAU	
	GAAUGGUACUGGACAAACU	
*LATS2* OTP pool	GCACGCAUUUUACGAAUUC	#L-003865-00-0005
	ACACUCACCUCGCCCAAUA	
	AAUCAGAUAUUCCUUGUUG	
	GAAGUGAACCGGCAAAUGC	
*RHOA OTP pool*	CGACAGCCCUGAUAGUUUA	# L-003860-00
	GACCAAAGAUGGAGUGAGA	
	GCAGAGAUAUGGCAAACAG	
	GGAAUGAUGAGCACACAAG	
*RAC1 OTP pool*	GUGAUUUCAUAGCGAGUUU	# L-003560-00
	GUAGUUCUCAGAUGCGUAA	
	AUGAAAGUGUCACGGGUAA	
	GAACUGCUAUUUCCUCUAA	
*RAC2 OTP pool*	UGACAACUAUUCAGCCAAU	# L-007741-00
	CCAAGGAGAUUGACUCGGU	
	CCAAGUGGUUCCCAGAAGU	
	UGAAAACCGUGUUCGACGA	
*RAC3 OTP pool*	AAACUGACGUCUUUCUGAU	# L-008836-00
	ACAAGAAGCUGGCACCCAU	
	GAAGACAUGCUUGCUGAUC	
	CGUGAUGGUGGACGGGAAA	
*CDC42 OTP pool*	CGGAAUAUGUACCGACUGU	# L-005057-00
	GCAGUCACAGUUAUGAUUG	
	GAUGACCCCUCUACUAUUG	
	CUGCAGGGCAAGAGGAUUA	

### Plasmid transfections

For transfections with Talin mApple and DOCK5-GFP, Effectene Transfection Reagent (Qiagen) was used according to manufacturer's protocol. Cells were plated at a density of 1.0 × 10^6^ cells per ml in 1 ml in a 6 well plate. Then, 2.56 ng of plasmid were combined with 150 μl of EC buffer and vortexed followed by addition of 8 μl of Enhancer and vortexing. After 10 minutes 5 μl of Effectene were added, mix was vortexed, 10 minute later 1 ml of DMEM was added and mixture was dispensed dropwise per well. If siRNA was used as well, cells were reverse transfected as usual. Experiments were performed 48 hours later.

### Generation of CAAX-GFP tagged cell line

CAAX-GFP tag was a kind gift from Nicola Ferrari (previously Institute of Cancer Research, currently Astex Pharmaceuticals, UK), who generated it in HEK-293 cells using a second-generation lentiviral vector (plasmid EGFP-CAAX pCSII-IRES2-hygro). Cells were plated at a density of 6.4 × 10^4^ cells per ml in a 6 well plate. Cells were transfected 24 hours later using a 1 : 1 dilution of lentivirus containing the CAAX-GFP tag solution and DMEM per well according to the manufacturers protocol. Transfected cells were selected with Hygromycin B (Invitrogen, cat#10687-010) at a final concentration of 500 μg ml^−1^ (elucidated from carrying out a concentration curve) 72 hours post transfection. CAAX tag did not affect YAP/TAZ ratio or cell morphology.

### Drug treatments

Drugs were usually added on top of media already present. Appropriate amounts of DMSO or H_2_O depending on what the drug was dissolved in were added to mock transfected cells. A list of drugs used can be found in Table 2. Drugs were added for a period of 4 hours, unless other stated, so as to eliminate any cell cycle effects.

**Table 2:** Drugs added

Drug	Inhibitor type/target	Company and catalogue number	Final concentration
PF-573228	FAK inhibitor	Tocris Biosciences, #3239; CAS 869288-64-2	2 μM^10^
C21	DOCK5 inhibitor	Alfa Aesar, #H60031.MD; CAS 54129-15-6	100 μM
Blebbistatin	Myosin II light chain	Sigma, #B0560; CAS 856925-71-8	10 μM
Binimetinib	MEK inhibitor	Selleckchem, 10 μM #S707	10 μM^132^
Uprosertib (GSK2141795)	AKT inhibitor		2 μM
Taxol (Paclitaxel)	Microtubule stabiliser	Sigma, #T7191	10 μM
Nocodazole	Microtubule depolymeriser	Sigma, #M1404	10 μM
LiCl	GSK3 inhibitor	Wako Chemicals	5 mM^92^

For the nocodazole and taxol experiment, cells were mock and si*DOCK5* reverse transfected as described in RNAi screening methodology for 48 hours. Then, cells were trypsinised and plated at a density of 1500 cells per well and allowed to settle for 20 hours. Microtubule depolymerisation was induced by nocodazole at a final concentration of 10 μM for 4 hours. Washout was performed for 30 minutes in either DMSO, 10 μM nocodazole, or 10 μM taxol – containing DMEM as indicated in Fig. 11.

### RNAi screening methodology

40 nl (0.08 pmol) of siRNA from an ONTARGET*Plus* human siRNA RhoGEF/GAP and Rho GTPase library (stock concentration of 20 μM, Dharmacon) were arrayed using the acoustic liquid handler Echo 550 prior to transfection and kept at −80 °C. All siRNAs were arrayed in duplicate per plate and each plate was arrayed in duplicate resulting in a total of 4 plates per library. Pre-stamped siRNA plates were thawed at room temperature for 30 minutes prior to use. 5 μl of Opti-MEM® Reduced Serum Media were then added per well followed by 5 μl of RNAiMax reagent (Invitrogen) and Opti-MEM® mix in a 1 : 125 ratio. Plates were spun down at 1000 rpm for 1 min and incubated at room temperature for 20 min. Cells (LM2 and MDA-MB-231) were then seeded at a concentration of 33 000 cells per ml (1000 cells per well ∼1.5 million cells per screen, 4 wells per siRNA, 4000 cells per siRNA per screen) in 30 μl DMEM supplemented with 12.8% FBS, 1.28% penicillin/streptomyocin (10% FBS and 1% penicillin/streptomyocin final) to account for the 10 μl of mix already present in the well, resulting in a total volume of 40 μl per well. Automated handling of plates on the Cell::Explorer robot station was carried out using the PlateWorks software (PerkinElmer). Plates were fixed 48 hours later with 8% warm formaldehyde in PBS (final concentration 4%) for 15 minutes and permeabilised with 0.1% Triton-X-100 in PBS for 10 minutes. Cells were then blocked in 2% BSA in PBS for 2 hours. All antibody incubations were performed in solution containing 0.5% BSA, 0.01% Triton-X-100 dissolved in PBS in a 1 : 1000 ratio, with the first primary antibody being added overnight at 4 °C and the rest being incubated at room temperature for 1.5 hours. Plates were washed 3× with PBS between every step using a Microplate Washer (BioTek). Antibodies were added sequentially to prevent cross reactivity. Antibodies used were: mouse YAP/TAZ (Santa Cruz Biotechnologies, cat#sc-101199), rat α-tubulin (Bio-Rad, cat#MCA77G), AlexaFluor 647 goat anti-mouse (Life Technologies, cat#A21235), AlexaFluor 647 goat anti-rat (Life Technologies, cat#A11077), phalloidin (Invitrogen, cat#A12379). Finally Hoechst (Sigma Aldrich, cat#33258) was added for 15 minutes, and after washing, cells were left in PBS and plates were sealed. Cells were imaged using an automated Opera Quadrupule Enhanced High Sensitivity (QEHS) spinning-disk confocal microscope (PerkinElmer) with 20× air lens. 28 fields of view were imaged per well. Cells were plated at increasing densities on columns 1, 2, 23 and 24 to be able to account for density dependent effects. SUM159 and hs578t were plated at a density of 67 000 cells per ml (2000 cells per well) and 50 000 cells per ml (1500 cells per well) respectively (∼2.5 million cells per screen, 4 wells per siRNA, 1500 to 2000 cells per well, 6000–8000 cells per siRNA per screen).

Images were processed and analysed using Columbus 2.6.0. Software Platform (PerkinElmer). Briefly, single cells were identified using the nuclear Hoechst signal. α-Tubulin intensity was using to segment the cytoplasm. Cells touching the image border were filtered out. Following this, cell morphology and texture features were extracted from all stains as described extensively in Pascual-Vargas *et al.*, *Scientific Data* 2017. In the same script, a cell shape linear classifier was manually trained on visually distinctive cells that represented each category (spindly, large round, triangular, fan, and small and round) and included all the features extracted from the stains except those based on YAP/TAZ. YAP/TAZ ratio was calculated as log ratio of nuclear to ring region (perinuclear region) and was not included in the classifier. The percentage of cells classified into each shape or YAP/TAZ ratio was calculated per well and normalized per plate. *Z*-Scores were then calculated per screen by taking away the average of all mock transfected cells from the RNAi containing well, and dividing by the standard deviation of all mock transfected cells. Each well was assigned a QMS consisting of the *Z*-score for each shape.

Because we have previously observed that following efficient gene depletion, many cells in a population can appear wild-type,^81^ which can potentially obscure the presence of phenotypes resulting from gene depletion, we identified and filtered normal cells from our data set in the following way. Briefly, we performed principal component analysis (PCA) on single cell dataset comprised of texture and morphological features of populations which were either mock transfected or transfected with siRNAs which scored the highest for enriching for a particular shape. The data were projected into 2D PC space and each data point (cell) colour coded by the shape of each cell (Fig. S5A, ESI†). We defined the region of shape space where different classified cells overlapped in PC space as normal cells, defined as those whose PC coordinates lay within 1 SD of the mean of PC1 and PC2 for the siRNA transfected population (black box, Fig. S5B, ESI†). We trained the new linear classifier on the cells that fell in this space in the mock transfected population (black box, Fig. S5A, ESI†) and defined this population as ‘normal’. Running the classifiers again on the same data, performing the same PCA analysis on this new dataset and removing cells classified as ‘normal’ resulted in cells classified into the different shapes separating better in PC shape space (Fig. S5C, ESI†). Further analyses are described in the results section.

### Quantitative real-time PCR (qRTPCR)

RNA was extracted using phenol:chloroform (TRIzol®, ThermoFisher cat#15596026) and RNAeasy kit (Qiagen cat#74104) according to the manufacturer's protocol. RNA was converted to cDNA using a cDNA conversion kit (Applied Biosystems cat#4387406) according to the manufacturers protocol. qRTPCR was performed on a QuantStudio® Flex Real-Time PCR system, using PCR mastermix SyBR green (Applied Biosystems cat#4309155) and primers in Table 3.

**Table 3:** PCR primers used for qRT-PCR

Gene	Forward (5′-3′)	Reverse (5′-3′)
DOCK5	GCTTTGAACTTCAGCTCTGG	CCACATGTCCCGGATTCTAA
WWTR1/TAZ	TATGGGACAGTCCGGGAGC	CGAGGCTTGGCTGACAAATC
YAP1	GACTTCCTGAACAGTGTGGA	CAGCCAAAACAGACTCCATG
GAPDH	AGATCCCTCCAAAATCAAG	GGCAGAGATGATGACCCTT

### 3D collagen invasion assay

We chose rat tail Col-I as our matrix because it is the most abundant type of collagen in mammalian tissues,^133–137^ and has been shown to form networks with physiological mechanical properties *in vitro*.^138–140^

Cells were reverse transfected in 6 well plates as previously described. 48 hours later cells were trypsinised and resuspended in a solution containing rat-tail collagen I (Corning cat#354249), ultrafiltered H_2_O (prepared in house according to standard protocols), DMEM 5× and HEPES (1 M pH 7.5, prepared in house according to standard protocols). Collagen solutions were prepared on ice and 96 well plates were prechilled prior to use. Collagen solutions were made up as in Table 4 starting with HEPES, followed by DMEM 5× with rat-tail collagen being added last. Neutralisation of collagen was tested with pH strips prior to use to ensure a pH of 7.4. DMEM 5× was made up using 12.5 g DMEM powder, 25 ml of 1 M HEPES ph 7.5, 5 g NaHCO_3_, H_2_O up to 250 ml and was filtered under sterile conditions before aliquoting into 10 ml batches and freezing them at −20 °C. Cells were spun down in 1.5 ml eppendorfs at 1000 rpm for 4 minutes, and supernatant was removed. Cell pellets were resuspended carefully so as to not introduce bubbles in 500 μl of collagen solution, and 100 μl of cell-collagen mix was dispensed per well of a 96 well plate. Once all conditions were dispensed, plates were spun down for 1 minute at 4 °C to ensure all cells were at the bottom of the plate. Collagen was allowed to polymerise at 37 °C for one hour. Then 50 μl of DMEM were added on top of the collagen gels. 150 μl of PBS were added to all outside wells to prevent gel dehydration and edge effects due to dehydration. Cells were fixed 48 hours later with 50 μl per well of 16% PFA containing Hoechst (1 : 500) for 2 hours. Gels were then washed with PBS and left in PBS prior to sealing. The Opera QEHS spinning-disk confocal microscope (PerkinElmer) with 20× air lens was used to image these plates. A minimum of three Z stacks were taken per well, spaced 30 μm apart with 0 μm referring to the bottom of the plate and subsequent stacks referring to inside the gel. 20 fields were imaged per well per stack, and were all in the centre of the well to prevent edge effects. Columbus software was used to quantify cell number at every stack, using a filtering threshold method to ensure only cells with a certain nuclear and GFP intensity were counted in each stack (Fig. S7, ESI†).

**Table 4:** Reagents used for collagen invasion assays

	Stock	Final concentration
Rat-tail collagen I	10.31 mg ml^−1^	2.0 mg ml^−1^
UF H_2_O (cold)	—	remaining
DMEM 5×	5×	1×
HEPES 1 M pH 7.5	100%	2%

### Spheroid invasion assay

Cells were reverse transfected in 96 well ultra-low attachment (ULA) plates as described in the siRNA transfections section. Quantities used per well: 20 μl OPTIMEM + 0.16 μl RNAimax, 20 μl OPTIMEM + 0.16 μl siRNA. Cells were plated at a density of 4.0 × 10^4^ cells per ml in 120 μl, resulting in a final volume of 160 μl per well. Due to their mesenchymal nature, LM2 cells required 2% Matrigel in ultra-low attachment plates to form spheroids, therefore 5 hours later a solution of 2.5% Matrigel in 40 μl of DMEM was added per well to promote spheroid formation resulting in a total volume of 200 μl per well. 48 hours later 180 μl of medium was carefully removed from each well so as not to disturb the spheroid, and replaced with 180 μl of 100% Matrigel. Spheroids were imaged prior to adding 100% Matrigel and immediately after using the Celigo Imaging Cytometer (Nexcelom Bioscience). Spheroids were imaged every day for 8 days, following the protocol outlined in ref. 141. Images were processed using ImageJ. Invasion area was determined by drawing a circle to the edge of where cells invaded and subtracting the initial area of the spheroid at day 0. Proliferation was quantified by drawing a circle around the edge of the area of the spheroid where the spheroid is grey, rather than the edge, so as to exclude any invading cells.

### Western blotting

Cells were reverse transfected in 6 well plates at a density of 3.3 × 10^4^ cells per ml in a 6 well plate (same density as screens and all other experiments unless otherwise stated) for 48 hours. Cells were harvested by trypsinising, spinning down in 1.5 ml eppendorfs and removing supernatant. Cells were then washed in PBS 3 times. Pellets were then resuspended in 500 μl of solution containing a ratio of 3 : 1 of water to LDS Sample Buffer (4×) (Invitrogen) with a final concentration of 2 mM DTT. Samples were then boiled at 100C for 10 minutes and spun down for 2 minutes at 13 000 rpm. Samples were frozen at −20 °C. Samples were boiled and spun down again prior to use. 16 μl of sample were loaded into 1.0 mm 15 well pre cast Novex 4–20% Tris–glycine gels (Invitrogen #XP04205BOX) and 30 μl into 10 well pre cast gels. Ladder used was Color Protein Standard, Broad Range (11–245 kDa) (New England Biolabs, #P7712S). Lysates were run in 1× Tris–glycine (Thermo) running buffer at 130 V until the bands reached the bottom of the gel. Gels were transferred in transfer buffer at 4 °C at 0.2 A onto a methanol activated polyvinylidene fluoride (PVDF) membrane (Immobilon-FL). Membranes were then stained with Ponceau (Sigma) dissolved in acetic acid to ascertain where the proteins were before cutting the membrane at required places. Membranes were then blocked in either 5% milk (Marvel) or 5% BSA if staining for phospho-proteins in Tris-buffered saline (TBS, prepared in house according to standard protocols) 0.1% Tween20 (TBST) solution for 1 hour at room temperature (RT)on a rolling shaker. Membranes were then washed once in TBST. Primary antibodies were diluted in either 5% milk TBST or 5% BSA TBST solution and incubated at 4 °C overnight on a rolling shaker. 10% glycerol was added to primary antibody solutions to be able to reuse them post storing at −20 °C. Membranes were washed 3 × 15 minutes in TBST. Membranes were then incubated for one hour at RT in either appropriate (mouse or rabbit) Horse radish peroxidase (HRP) – linked secondary antibodies (cell signaling) in TBST for chemiluminescent detection. Membranes were washed 3 × 5 minutes with TBST. Membranes were treated with ECL substrate (Thermo, #32106) and exposed using Azure Biosystems c600 western blot imaging system. Descriptions of antibodies used and dilutions can be found in Table 5. Western Blots were quantified using Image J and normalised to GAPDH unless otherwise stated.

**Table 5:** Primary antibodies used for immunofluorescence (I.F) and western blot (W.B)

Target	Antibody and species	Company and cat number	W.B dilution	I.F Dilution
YAP/TAZ	Anti-YAP/TAZ (mouse)	Santa Cruz Biotechnologies, cat#A21235	1 : 1000	1 : 1000
α-Tubulin	Anti-α-tubulin (rat)	Bio-Rad, cat#MCA77G		1 : 1000
GSK3β	Anti-GSK3β (rabbit)	CST, cat#12456T		1 : 300
pGSK3β	Anti-phospho GSK3β (Ser9) (D85E12) (rabbit)	CST, cat#5558T		1 : 400
Golgi	Anti-GM180 (mouse)	Abcam, cat #52649		1 : 500
Beta-catenin	Anti-beta-catenin (mouse)	BD Transduction Laboratories, cat#610153		1 : 1000
FOXO3A	Anti-FOXO3A (75D8) (rabbit)	CST, cat#2497S		1 : 200
DOCK5	Anti-DOCK5 (rabbit)	Frank *et al.*, 2017.	1 : 500	
PHH3	Anti-P-Histone H3 (S10) (mouse)	CST, cat#9706S	1 : 1000	
KIF5B	Anti-KIF5B (rabbit)	Protein Tech, cat #231632-1-AP	1 : 1000	
PCNA	Anti-PCNA (mouse)	Santa Cruz Biotechnology, cat #sc-56	1 : 1000	
GAPDH	Anti-GAPDH (1D4) (mouse)	Novus, cat#NB300-221	1 : 2000	
Paxillin	Anti-Paxillin (mouse)	BD transduction labs#610052		1 : 300

### Subcellular fractionation

Thermo's Subcellular Protein Fractionation Kit for Cultured Cells (#78840) was used according to the manufacturer's protocol to extract cytoplasmic, membrane, nuclear soluble, chromatin-bound and cytoskeletal proteins. Briefly, cells were harvested by trypsining them and centrifuging for 5 minutes. Cell pellets were washed with ice-cold PBS. Cells were counted so as to be able to use the corresponding ratios of cytoplasmic extraction buffer (CEB), membrane extraction buffer (MEB), nuclear extraction buffer (NEB) and pellet extraction buffer (PEB) : CEB : MEB : NEB : PEB 200 : 200 : 100 : 100 μl respectively. Fractions were stored at −80 °C.

Protein concentrations of each subcellular fraction were measured using the Pierce BCA Protein Assay kit (Thermo). Samples were diluted in water (Sigma) to the lowest sample concentration to ensure the same amount of protein for each fraction was loaded into the gels. 4 μl of NuPage LDS Sample Buffer (4×) were added to 16 μl of each sample, which were then boiled for 10 minutes and spun down before freezing. Samples were then run as described in western blotting subsection.

### Mass spectrometry

#### Preparation and TMT labelling

LM2, MDA-MB-231, and hs578t cells were reverse transfected with siRNA targeting *DOCK5*. LM2 and MDA-MB-231 were both plated at a density of 3.3 × 10^4^ cells per ml (low density, siRNA screening density) and 1.0 × 10^5^ cells per ml (high density) in T75 falcons due to the requirement of 3 million cells per sample. hs578t cells were plated at 5.0 × 10^4^ cells per ml. 48 hours later cells were collected by trypsinization and washed 3× with cold PBS in clean mass spectrometry tubes. Samples were then flash frozen with 70% ethanol and dry ice. Cell pellets were dissolved in 150 μl lysis buffer containing 1% sodium deoxycholate (SDC), 100 mM triethylammonium bicarbonate (TEAB), 10% isopropanol, 50 mM NaCl and Halt protease and phosphatase inhibitor cocktail (100×) (Thermo, #78442) on ice, assisted with pulsed probe sonication for 15 s. Samples were subsequently boiled at 90 °C for 5 min on a thermomixer and sonicated for a 5 s additionally. Protein concentration was measured with the Coomassie Plus Bradford Protein Assay (Pierce) according to manufacturer's instructions. Aliquots containing 100 μg of total protein were prepared for trypsin digestion. Samples were reduced with 5 mM tris-2-carboxyethyl phosphine (TCEP) for 1 h at 60 °C and alkylated with 10 mM iodoacetamide (IAA) for 30 min in dark. Proteins were then digested by adding trypsin (Pierce) at final concentration 75 ng μl^−1^ to each sample and incubating overnight. The resultant peptides were labelled with the TMT-10plex multiplex reagents (Thermo) according to manufacturer's instructions and were combined in equal amounts to a single tube. The combined sample was then dried with a centrifugal vacuum concentrator.

#### High-pH reversed-phase peptide fractionation and LC–MS/MS analysis

Offline high pH reversed-phase (RP) peptide fractionation was performed with the XBridge C18 column (2.1 × 150 mm, 3.5 μm, Waters) on a Dionex Ultimate 3000 high performance liquid chromatography (HPLC) system. Mobile phase A was 0.1% ammonium hydroxide and mobile phase B was 100% acetonitrile, 0.1% ammonium hydroxide. The TMT labelled peptide mixture was reconstituted in 100 μl mobile phase A and was fractionated using a gradient elution method at 0.2 ml min^−1^ with the following steps: for 5 minutes isocratic at 5% B, for 35 min gradient to 35% B, gradient to 80% B in 5 min, isocratic for 5 minutes and re-equilibration to 5% B. Fractions were collected every 42 s and vacuum dried.

LC–MS/MS analysis was performed on the Dionex Ultimate 3000 system coupled with the Q Exactive HF Orbitrap Mass Spectrometer (Thermo Scientific). Each peptide fraction was reconstituted in 40 μl 0.1% formic acid and 7 μl were loaded to the Acclaim PepMap 100, 100 μm × 2 cm C18, 5 μm, 100 Å trapping column at 10 μl min^−1^ flow rate. The sample was then subjected to a gradient elution on the EASY-Spray C18 capillary column (75 μm × 50 cm, 2 μm) at 45 °C. Mobile phase A was 0.1% formic acid and mobile phase B was 80% acetonitrile, 0.1% formic acid. The gradient separation method at flow rate 250 nl min^−1^ was as follows: for 90 min gradient from 5%-38% B, for 10 min up to 95% B, for 5 min isocratic at 95% B, re-equilibration to 5% B in 5 min, for 10 min isocratic at 5% B. The top 15 precursor ions between 350–1850 *m/z* were selected with mass resolution of 120k, AGC 3 × 10^6^ and max IT 50 ms for HCD fragmentation with isolation width 1.0 Th. Collision energy was set at 35% with AGC 1 × 10^5^ and max IT 100 ms at 60k resolution. Targeted precursors were dynamically excluded for 30 seconds.

#### Database search and protein quantification

The SequestHT search engine was used to analyse the acquired mass spectra in Proteome Discoverer 2.2 (Thermo Scientific) for protein identification and quantification. The precursor mass tolerance was set at 20 ppm and the fragment ion mass tolerance was set at 0.02 Da. Spectra were searched for fully tryptic peptides with a maximum of 2 miss-cleavages. TMT6plex at N-terminus/K and Carbamidomethyl at C were defined as static modifications. Dynamic modifications included oxidation of M and Deamidation of N/Q. Peptide confidence was estimated with the Percolator node. peptide false discovery rate (FDR) was set at 0.01 and validation was based on *q*-value and decoy database search. All spectra were searched against reviewed UniProt human protein entries. The reporter ion quantifier node included a TMT 10plex quantification method with an integration window tolerance of 15 ppm and integration method based on the most confident centroid peak at the MS2 level. Only unique peptides were used for quantification, considering protein groups for peptide uniqueness. Peptides with average reporter signal-to-noise >3 were used for protein quantification.

Abundance values were scaled within each cell line and the log 2 ratio of abundance in si*DOCK5* containing samples to mock transfected samples was calculated. Log2 ratios above 0.5 and below −0.5 and permutation FDR < 0.05 (*t*-test) were considered to be proteins which were significantly up (∼40% minimum increase) and down regulated (∼40% minimum decrease) respectively. Statistical analysis of proteomics data was done in the Perseus software.^142^

### Microscopy

#### Immunofluorescence and confocal microscopy

All performed as described for siRNA screening. Antibodies and their dilutions can be found in Tables 5 and 6. Unless otherwise stated cells were imaged using an automated Opera Quadruple Enhanced High Sensitivity (QEHS) spinning-disk confocal microscope (PerkinElmer) with 20× air lens. Lasers and their corresponding filters used were: 405(450/50), 561 (600/40), 488 (540/751), 640 (690/50).

**Table 6:** Secondary antibodies used for Immunofluorescence (I.F) and Western Blot (W.B)

Secondary antibody/dyes	Company	Dilution
Phalloidin	Invitrogen, cat#A12379	1 : 1000
Hoechst	Sigma Aldrich, cat#33258	1 : 1000
AlexaFluor 647 goat anti-mouse	Life Technologies, cat#A21235	1 : 1000
AlexaFluor 568 goat anti-rat	Life Technologies, cat #A11077	1 : 1000
AlexaFluor 647 goat anti-rabbit	Life Technologies, cat #A21245	1 : 1000
AlexaFluor 568 goat anti-rabbit	Life Technologies, cat #A11036	1 : 1000
Anti-mouse IgG, HRP-linked antibody	CST, cat #7076S	1 : 2000
Anti-rabbit IgG, HRP-linked antibody	CST, cat #7074S	1 : 2000

#### Focal adhesion dynamics and total internal reflection fluorescence imaging (TIRF)

LM2 CAAX-GFP cells were transfected with either just Talin mApple plasmid or both Talin mApple and si*DOCK5*, si*RAC*, si*RHOA*, or si*CDC42* as stated in the transfections section. 35 mm glass Mat-tek dishes were coated with 10 μg ml^−1^ Fibronectin (human plasma, Sigma #F0895) for 1 hour. Fibronectin was then aspirated and Mat-tek dishes were washed 3× PBS and left to dry. 48 hours after transfection, LM2 CAAX-GFP cells were plated at 1.5 × 10^5^ cells per ml in 1 ml of Liebnevitz medium (10% FBS, 1% penicillin/streptomyocin). Cells were live imaged using the TIRF (3i) approximately 1 hour after seeding. Lasers used were 647 And 488. Images were taken either every 3 minutes or every 1.5 minutes. Images were acquired using Slidebook software (3i). Movies were processed to be the same length and analysed using the ‘Focal Adhesion Analysis Server (FAAS)’ (Berginski and Gomez, 2013). Movies containing the same number of time frames were compared.

We displayed the data using estimation statistics using a website generated by Ho *et al.*, 2019,^106^ as we think that by showing the effect size we can visualise and understand this type of data better.

#### Spreading assay and super-resolution *via* optical-reassignment (SoRa) microscopy

Cells pertaining to experiments imaged using the SoRa were fixed and stained with a slight variation to those from RNAi screens. Briefly, cells were reverse transfected as described in the transfection section in 6 well plates, and plated on 35 mm glass Mat-tek dishes coated with 10 μg ml^−1^ Fibronectin as described for TIRF, at a density of 1.5 × 10^5^ cells per ml 48 hours after transfection. They were fixed 3 hours after plating with paraformaldehyde in PBS (4% final concentration), permeabilised with 0.1% Triton-X-100 in PBS for 10 minutes, and blocked with 0.5% BSA dissolved in PBS with 0.3 ml of glycine for 1 hour. Antibody incubations were performed in 0.5% BSA. Primary antibodies were added at RT for 1.5 hours. Secondary antibodies were added for 1.5 hours at RT. Hoechst was used as a DNA stain and left for 15 minutes at the end at RT. Cells were washed 3× with PBS between each step and were left in 200 μl of PBS. Where antibodies used were close in species such as mouse and rat, they were added sequentially to avoid any possible bleedthrough. Antibodies and their dilutions can be found in Tables 5 and 6. When drugs were used, they were added in 500 μl on top of the 2 ml solution of cells immediately after plating, with final concentrations as shown in Table 2. Cells were imaged using the SoRa (3i) microscope.

### Wound heal assay

Cells were reverse transfected as previously described and were plated in a 96 well PerkinElmer Cell Carrier Ultra plate at a density of 2.7 × 10^6^ cells per ml. 48 hours later a 10 μM pipette tip was used to scratch the monolayers from top to bottom of the well, resulting in a vertical cell-free gap or wound. Medium was removed and wells were carefully washed with warm PBS so as to remove debris. 100 μl of fresh medium was them added to all wells. Imaging began as soon as the wound was created, using a Basic Confocal Spinning Disk Microscope (3i). Images were taken every 10 minutes for 16 hours. Images were processed using ImageJ. We quantified Golgi orientation in cells that had their Golgi within 180 degrees of the wound and quantified the angle of orientation by drawing a line from the position of the Golgi towards the wound and another parallel to the wound (Fig. 7C and D) as in Zhang *et al.*, 2008.^102^ A 90 degree angle is indicative of Golgi facing toward the wound and indicative of front-rear polarity.

### Growth curve assay

Cells were reverse transfected in 6-well plates and plated at a density of 3.3 × 10^4^ cells per ml per well, with three replicates per condition. When drugs were used one plate containing mock transfected cells and DOCK5 siRNA would be treated with DMSO, and another with the drug. Binimetinib (MEKi) and FAKi (PF-573288) were added to a final concentration of 10 μM and 2 μM respectively. Drugs and DMSO were added in 500 μl DMEM 48 hours after transfection. Plates were incubated in the IncuCyte S3 Live-Cell Analysis System (Sartorius) at 37 °C and 5% CO_2_ and imaged at 6 hour time points for a minimum of 156 hours (6.5 days). A minimum of 9 fields of view were imaged per well. Analysis of confluence over time was carried out using IncuCyte software (Sartorius). To compare confluence between conditions at a point where mock transfected cells reached 50% or 100% confluency, each well was normalized to the mock transfected wells for each plate, prior to statistical analysis. Representative growth curves are shown per plate with the average of three wells per condition.

### Statistical analyses

Statistical analyses performed for each assay are indicated in text and figure legends. Most analyses were carried out using Prism 8 (GraphPad). PCA analysis was performed in Jupyter Notebooks.^143^ Hierarchical clustering and heat maps were made with Morpheus Broad Institute Software (https://software.broadinstitute.org/morpheus).^144^

## Author contributions

P. P.-V. contributed to experimental design, carried out the experiments, performed image and data analysis, wrote and prepared the manuscript. M. A.-G. optimized RNAi screening methodology, performed experiments, data and image analysis. T. I. R, J. S. C performed mass spectrometry and proteomic analysis. C. B contributed to experimental design, imaging, and manuscript writing and preparation.

## Conflicts of interest

The authors declare no competing interests.

## Supplementary Material

MO-021-D4MO00154K-s001

MO-021-D4MO00154K-s002

MO-021-D4MO00154K-s003

MO-021-D4MO00154K-s004

MO-021-D4MO00154K-s005

MO-021-D4MO00154K-s006

MO-021-D4MO00154K-s007

MO-021-D4MO00154K-s008

MO-021-D4MO00154K-s009

## Data Availability

Proteomic data available on PRIDE database: PXD020102. Morphological and YAP/TAZ staining data in LM2 and MDA-MB-231 cells following RhoGEF/GAP knockdown is available from P. Pascual-Vargas, *et al.*, RNAi screens for Rho GTPase regulators of cell shape and YAP/TAZ localisation in triple negative breast cancer., *Sci. Data*, 2017, **4**, 170018.
