# Supplementary figures and images for: Integration of focal adhesion morphogenesis and polarity by DOCK5 promotes YAP/TAZ-driven drug resistance in TNBC
Source: Mol Omics. 2025 May 12;21(5):390–421. doi: 10.1039/d4mo00154k (PMC12068046; doi:10.1039/d4mo00154k)

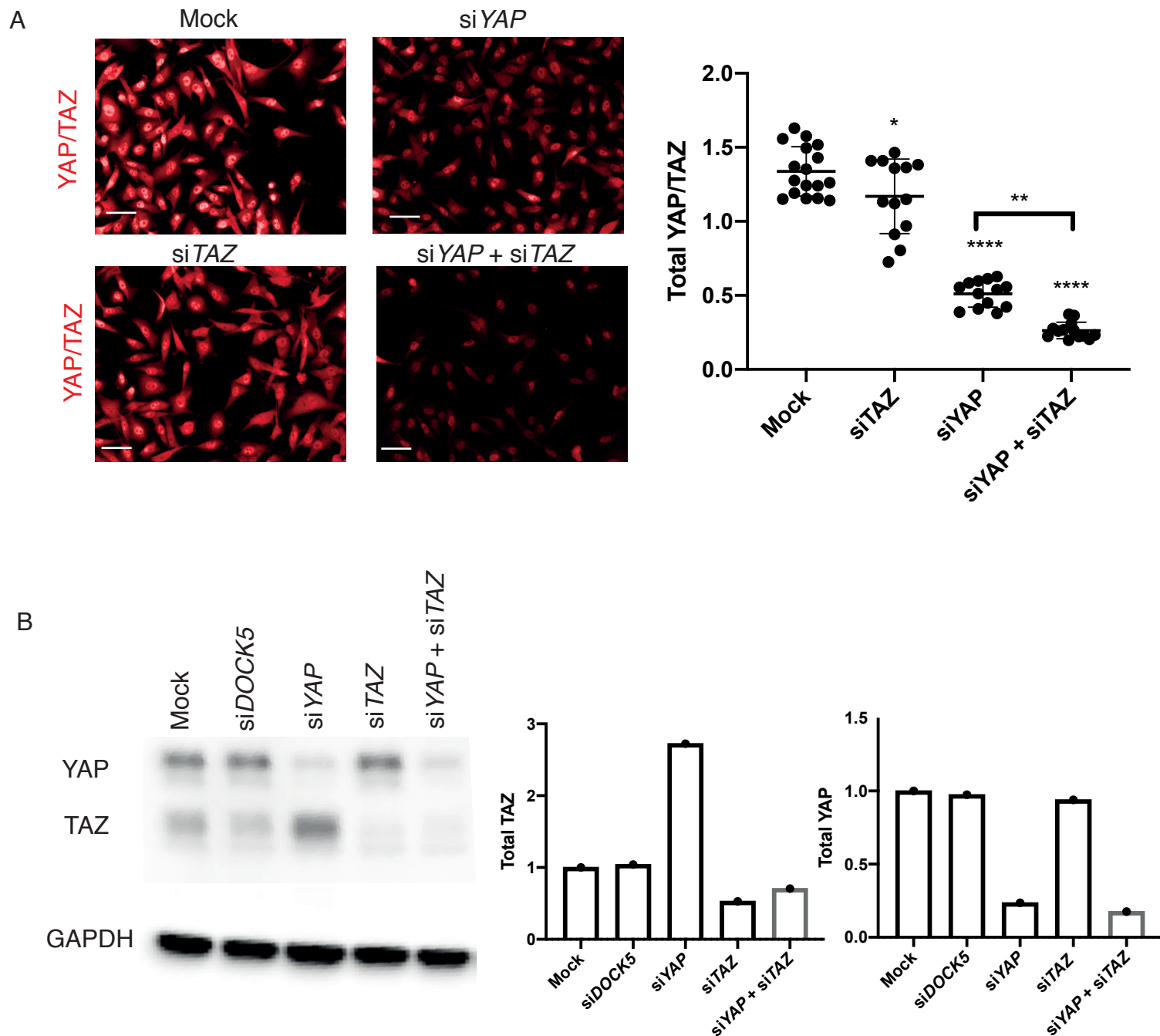

Supplement: MO-021-D4MO00154K-s001 [file MO-021-D4MO00154K-s001.pdf]

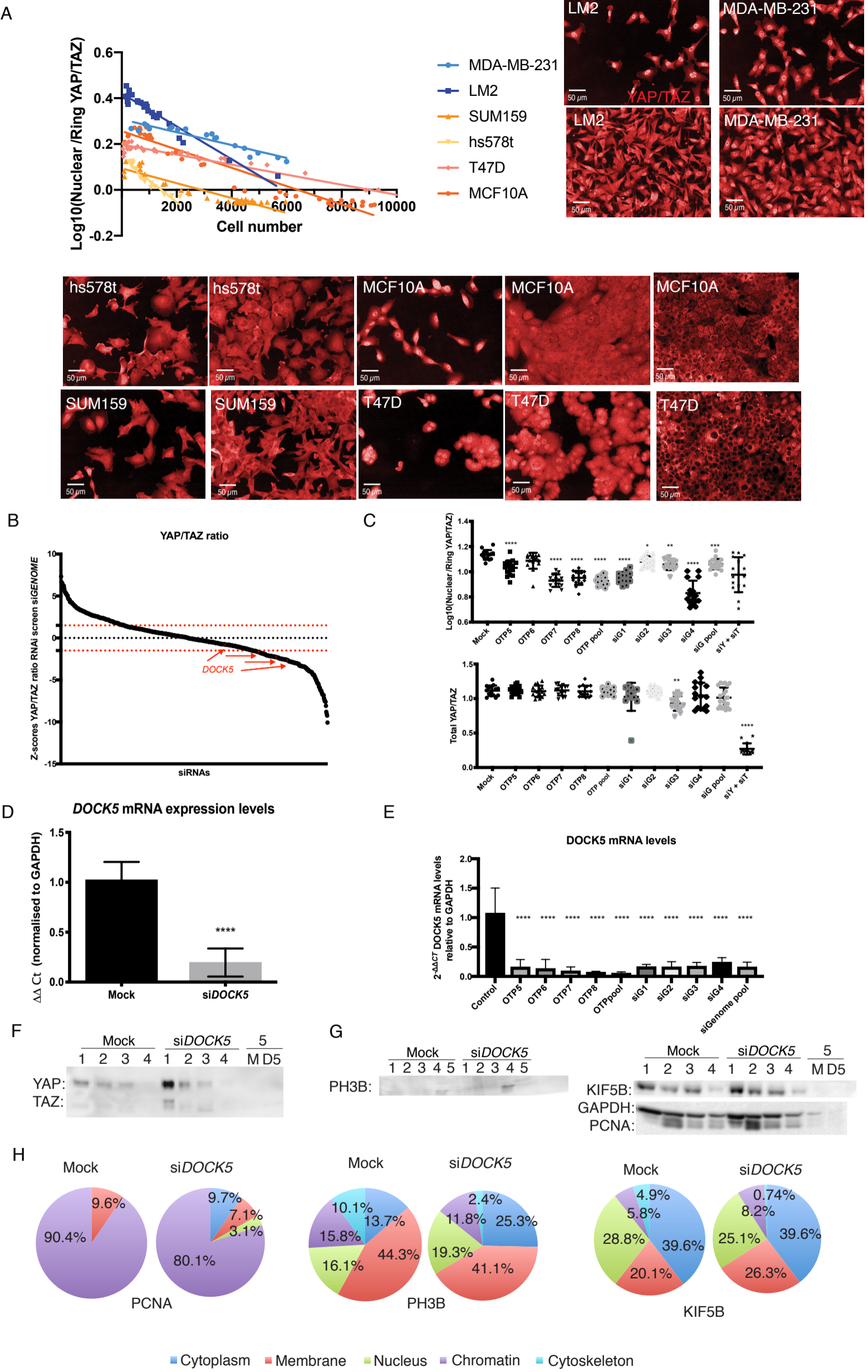

Supplement: MO-021-D4MO00154K-s002 [file MO-021-D4MO00154K-s002.pdf]

**A** MDA-MB-231 ONTargetPlus

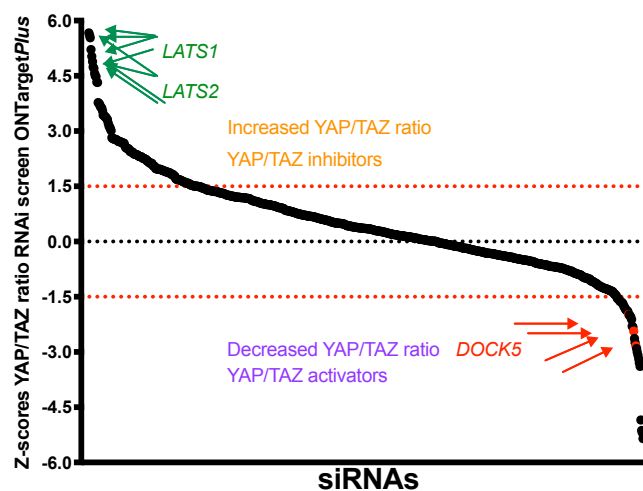

**B** MDA-MB-231 siGENOME

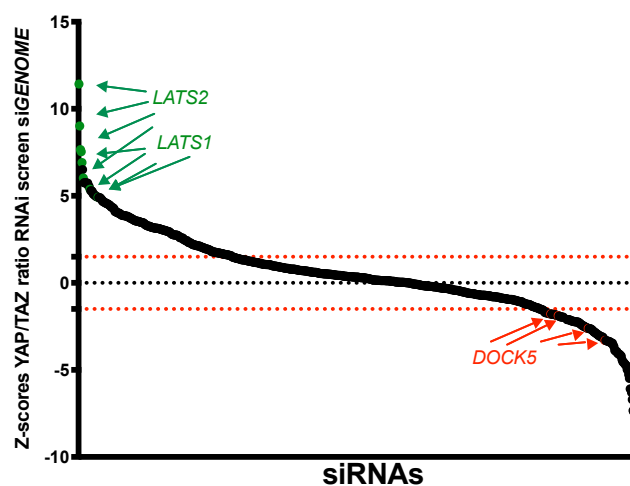

Supplement: MO-021-D4MO00154K-s003 [file MO-021-D4MO00154K-s003.pdf]

Supplementary Figure 4 RHOA, RAC1, YAP, TAZ, depletion do not affect growth rates

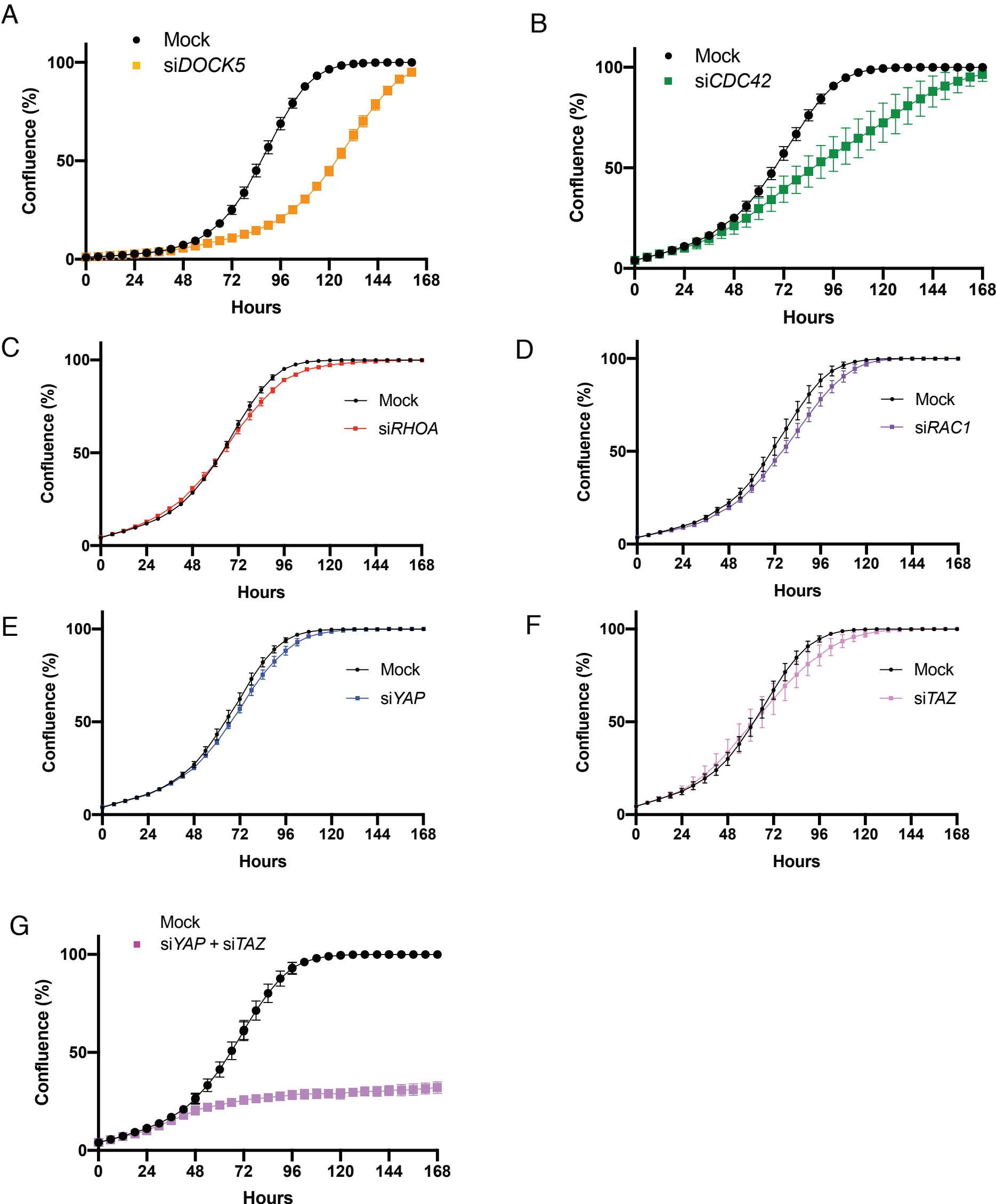

Supplement: MO-021-D4MO00154K-s004 [file MO-021-D4MO00154K-s004.pdf]

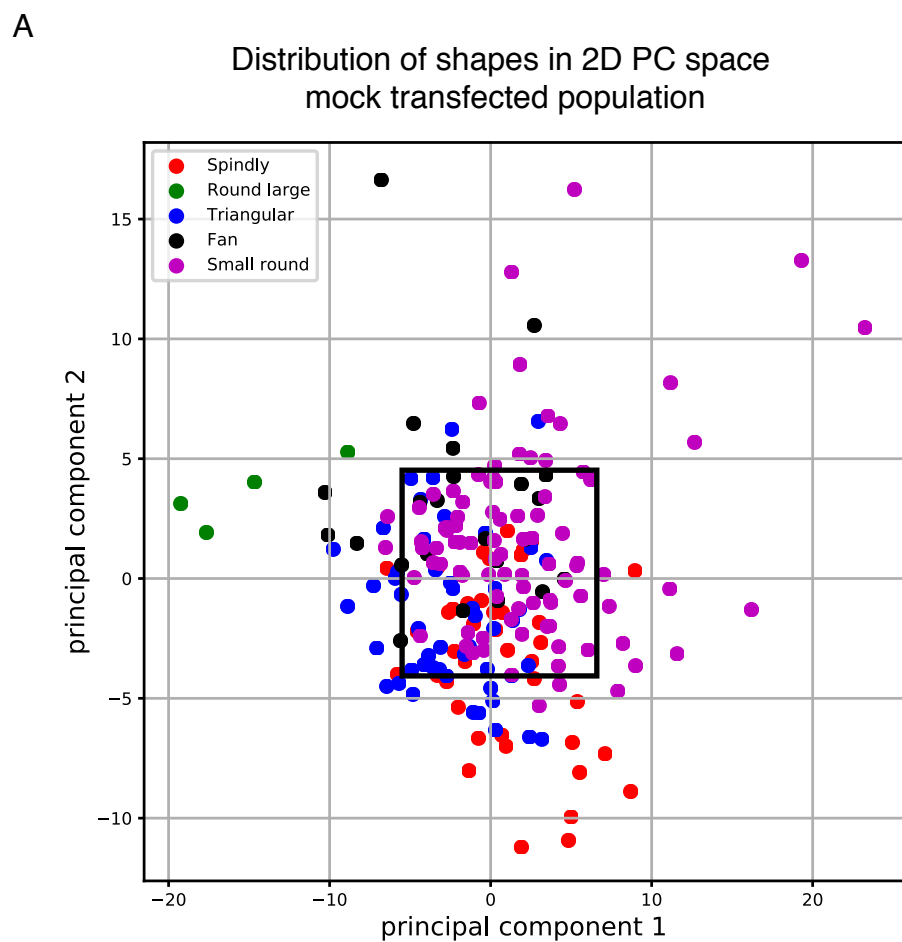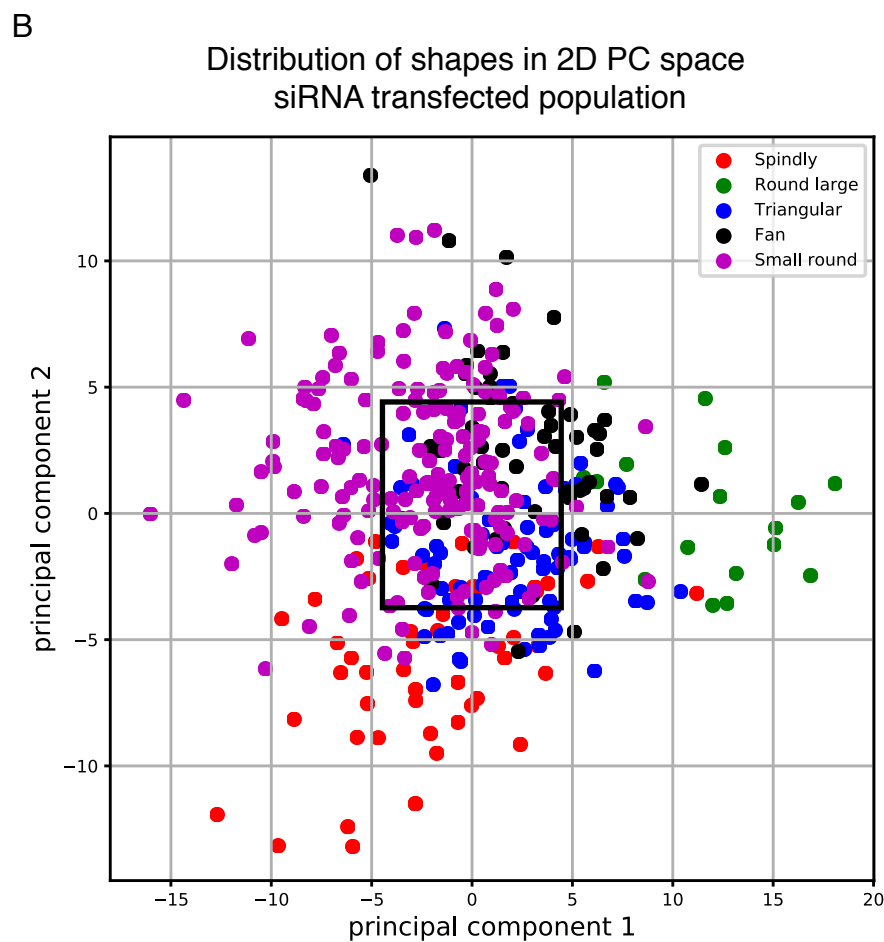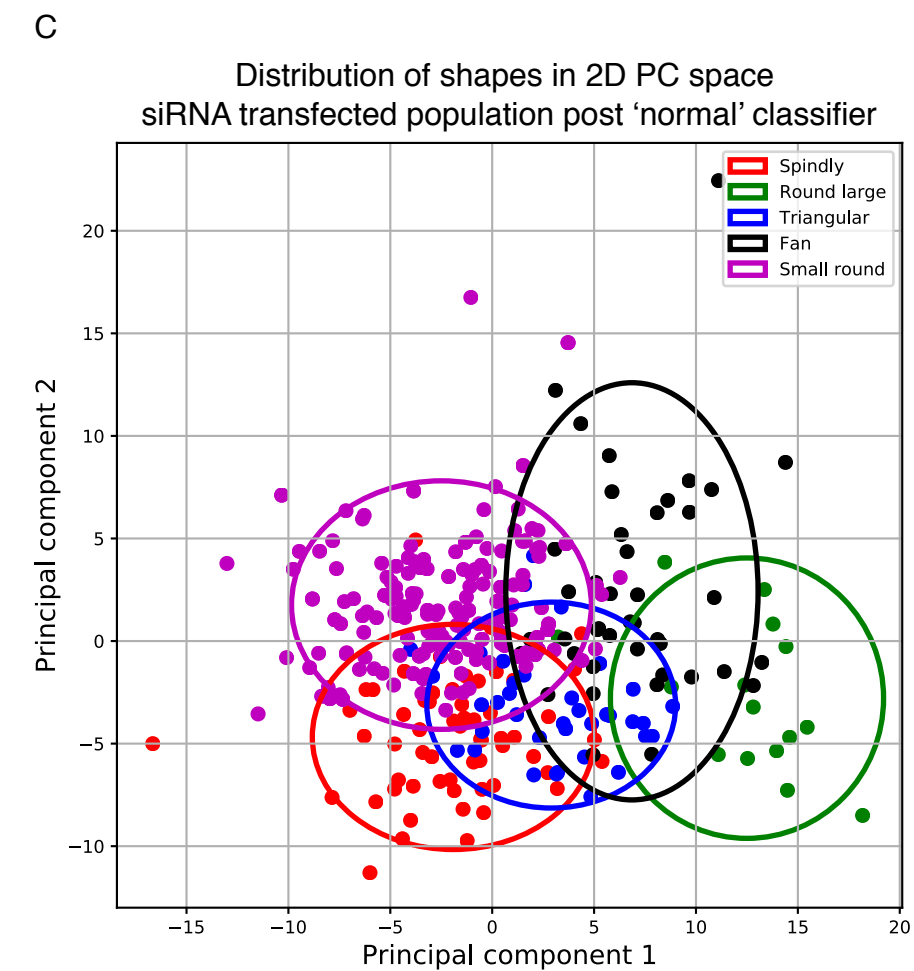

Supplement: MO-021-D4MO00154K-s005 [file MO-021-D4MO00154K-s005.pdf]

# Supplementary Figure 7. Automated segmentation for Collagen Invasion Quantification

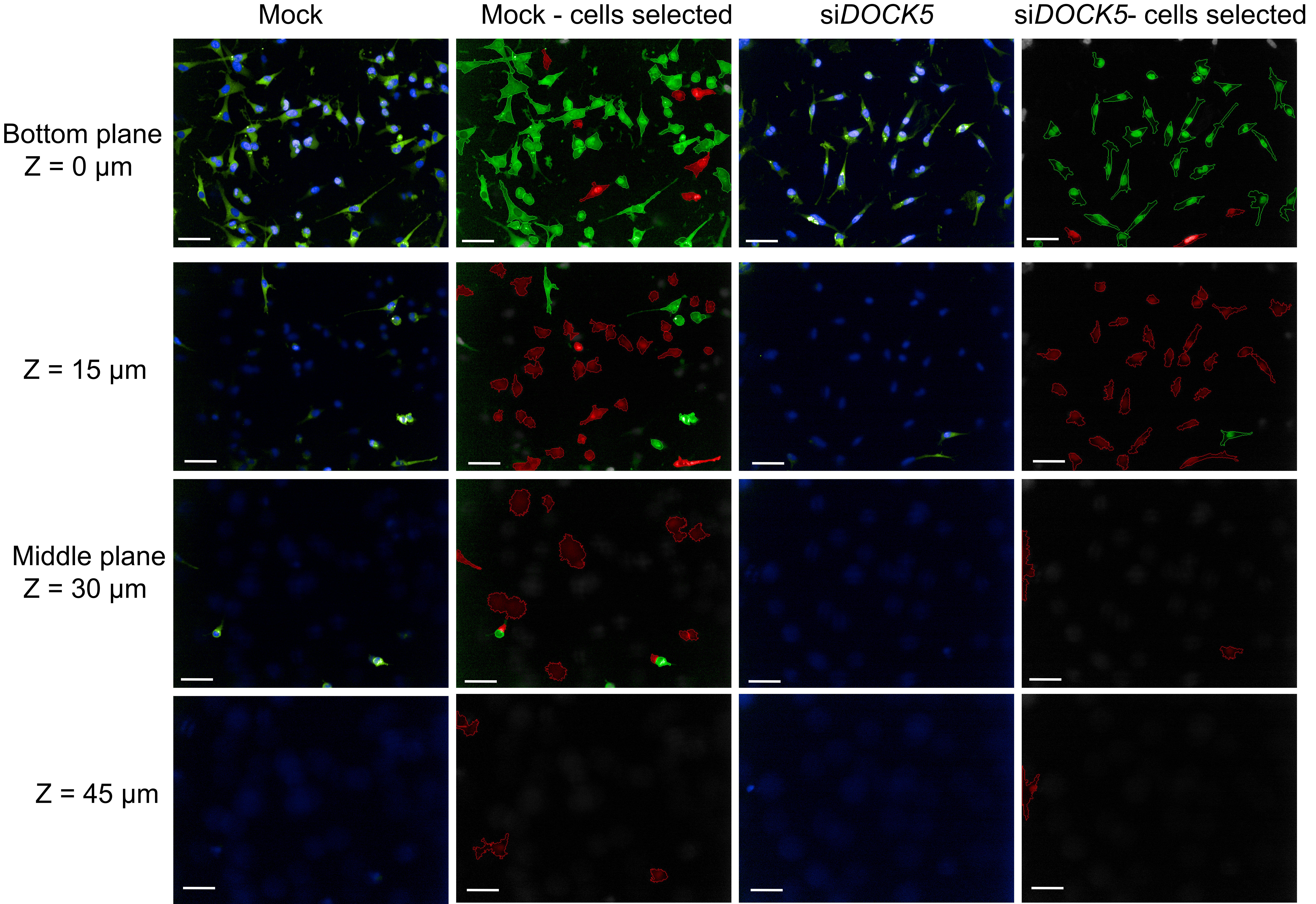

Supplement: MO-021-D4MO00154K-s007 [file MO-021-D4MO00154K-s007.pdf]

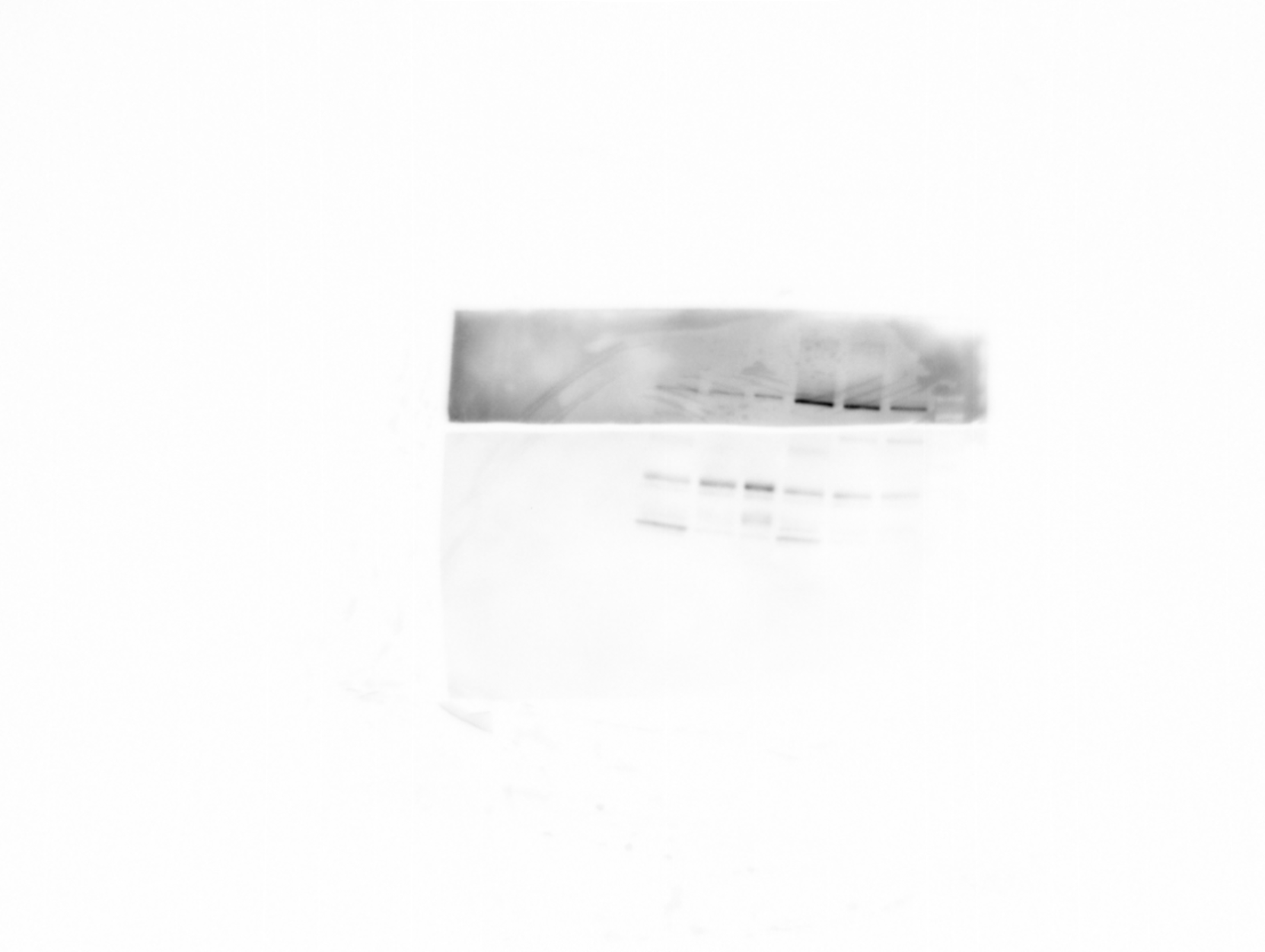

Supplement: MO-021-D4MO00154K-s009 [file MO-021-D4MO00154K-s009.zip › raw image figure 1d _1_.tif]

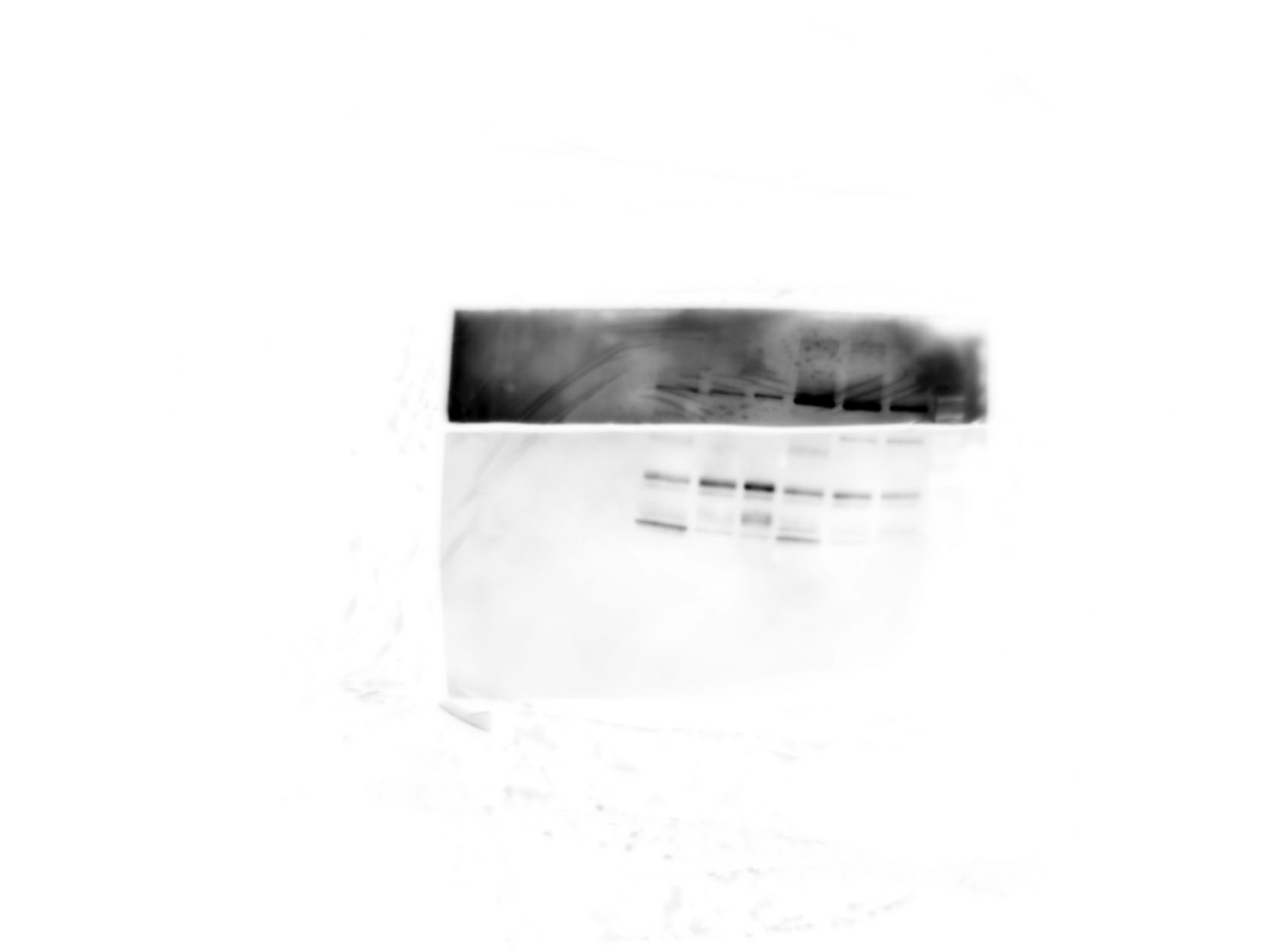

Supplement: MO-021-D4MO00154K-s009 [file MO-021-D4MO00154K-s009.zip › raw image figure 1d _2_.tif]

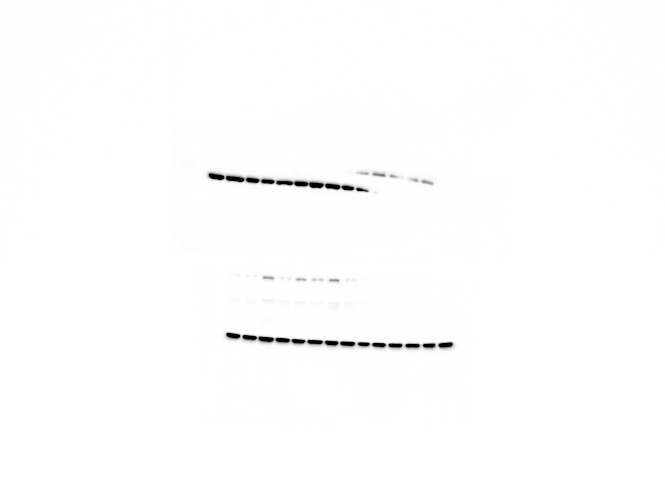

Supplement: MO-021-D4MO00154K-s009 [file MO-021-D4MO00154K-s009.zip › raw image figure s1b gapdh.tif]

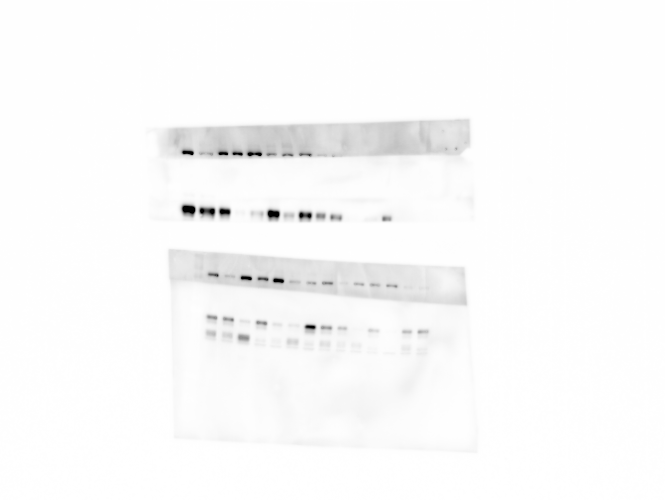

Supplement: MO-021-D4MO00154K-s009 [file MO-021-D4MO00154K-s009.zip › raw image figure s1b yaptaz and dock5.tif]

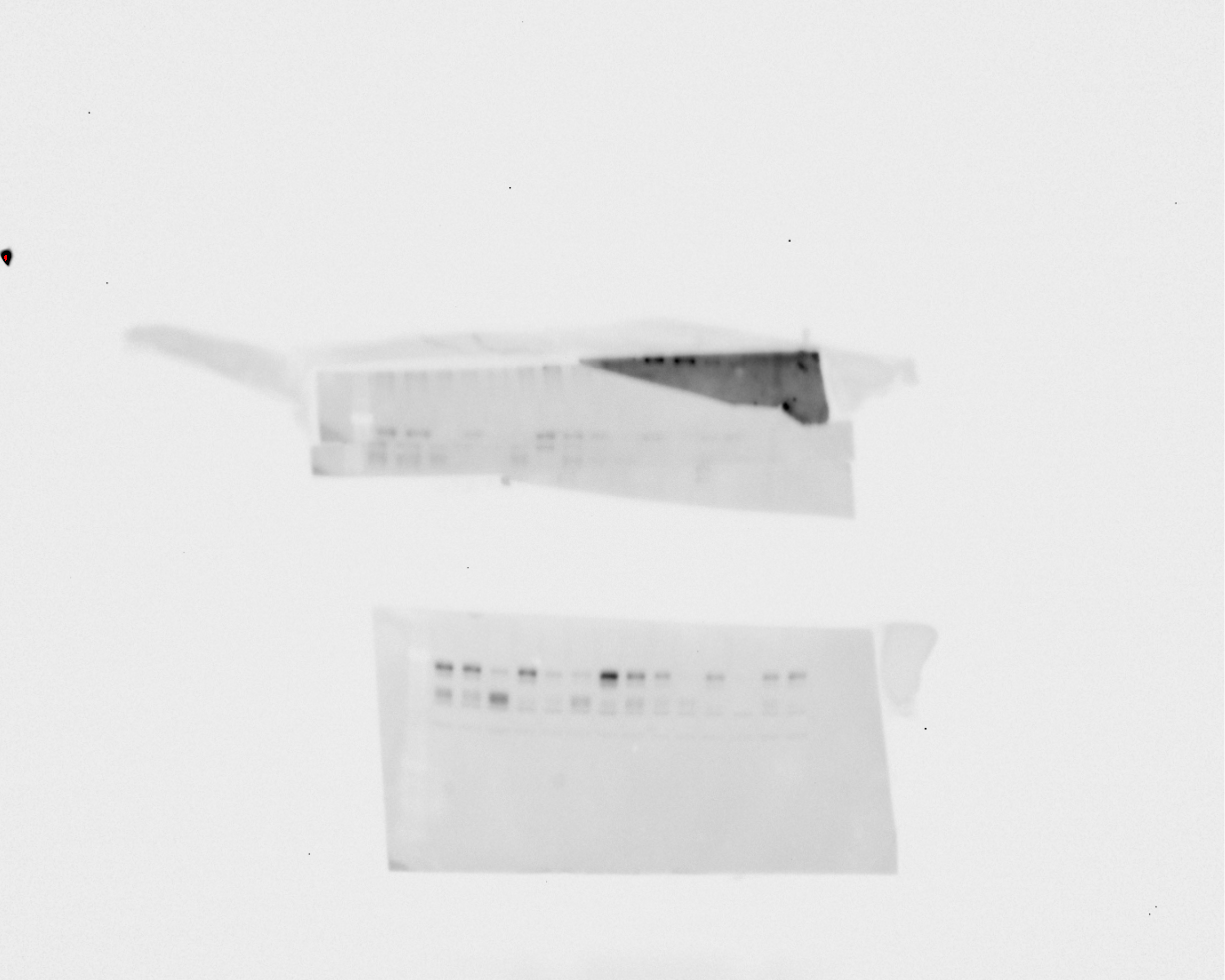

Supplement: MO-021-D4MO00154K-s009 [file MO-021-D4MO00154K-s009.zip › raw image figure s1b yaptaz.tif]

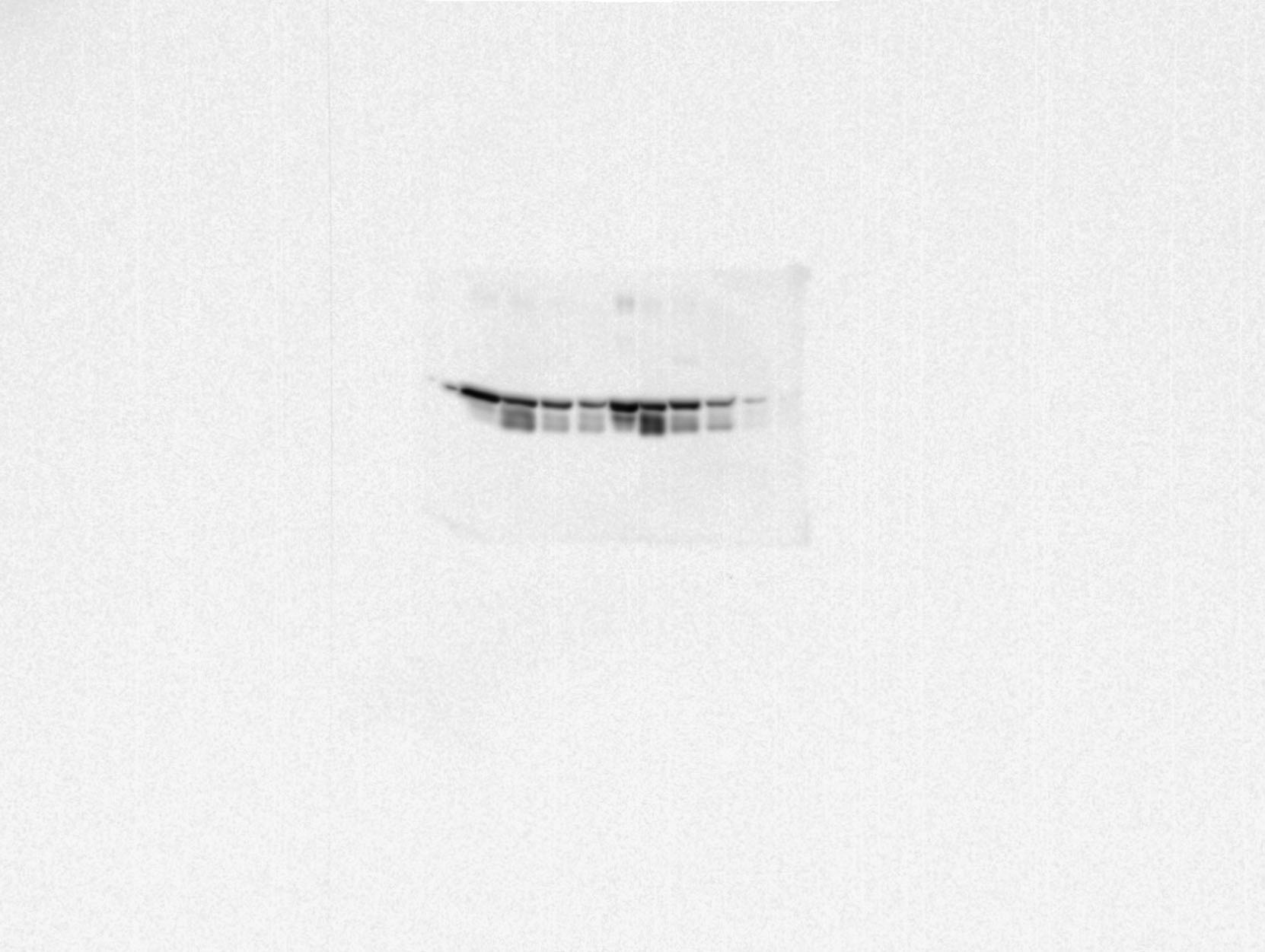

Supplement: MO-021-D4MO00154K-s009 [file MO-021-D4MO00154K-s009.zip › raw image figure s2f and g gapdh and pcna.jpg]

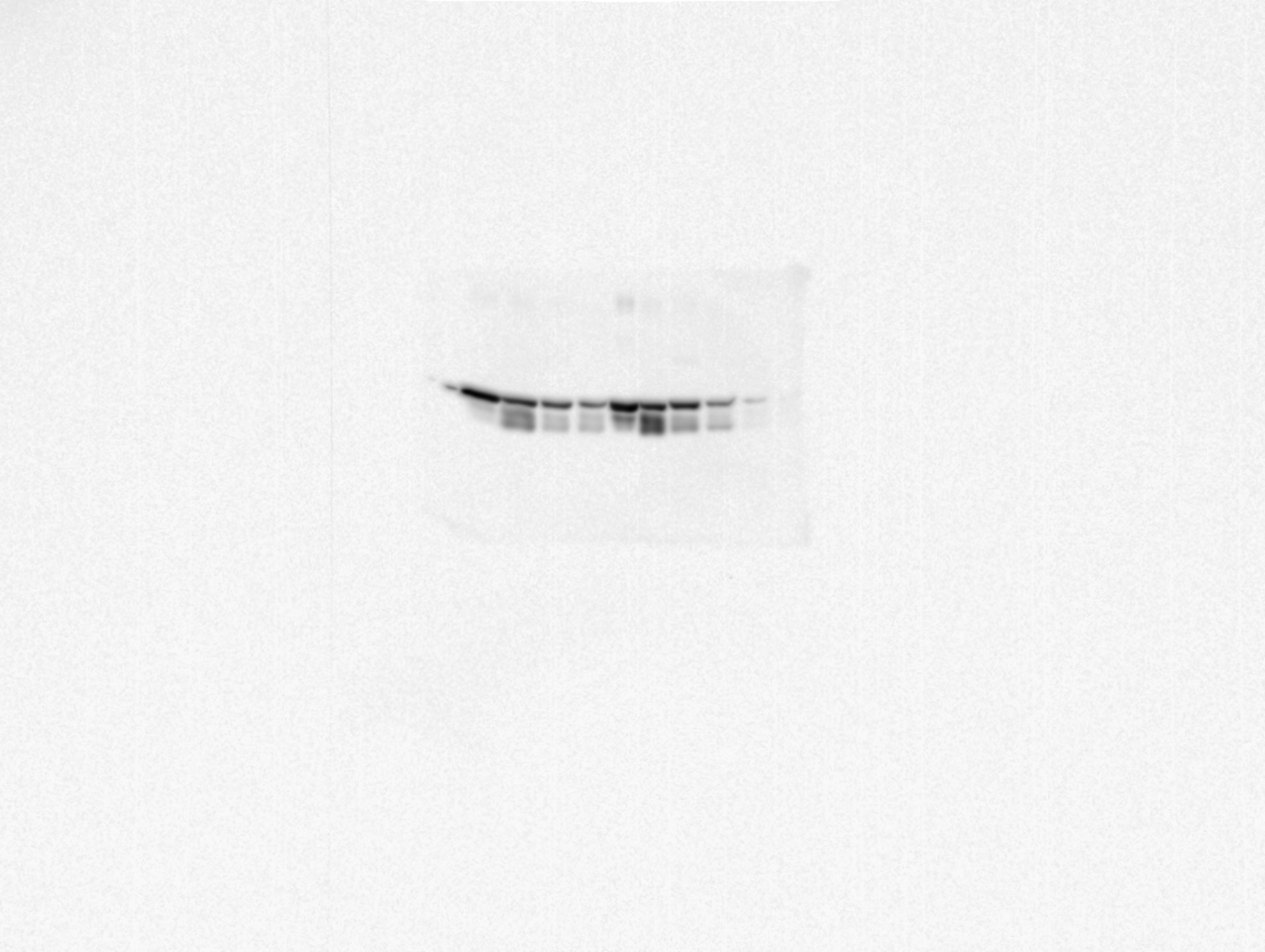

Supplement: MO-021-D4MO00154K-s009 [file MO-021-D4MO00154K-s009.zip › raw image figure s2f and g gapdh and pcna.tif]

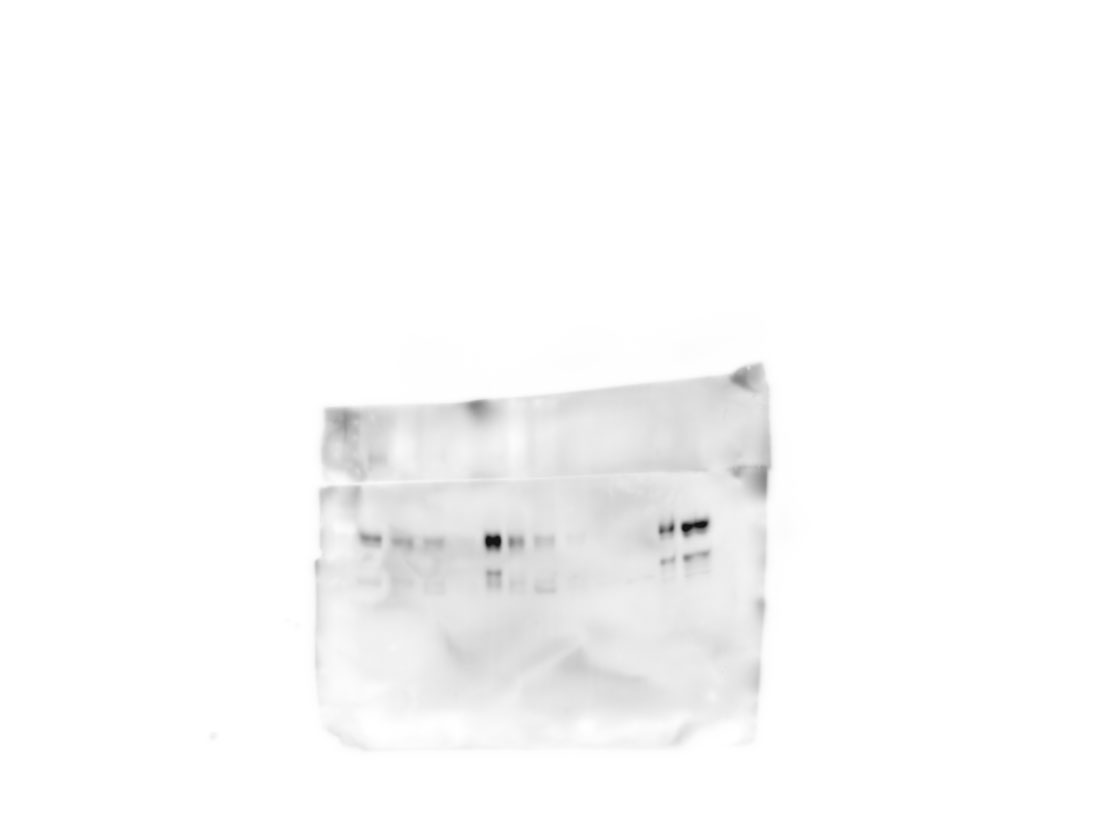

Supplement: MO-021-D4MO00154K-s009 [file MO-021-D4MO00154K-s009.zip › raw image figure s2f yaptaz.jpg]

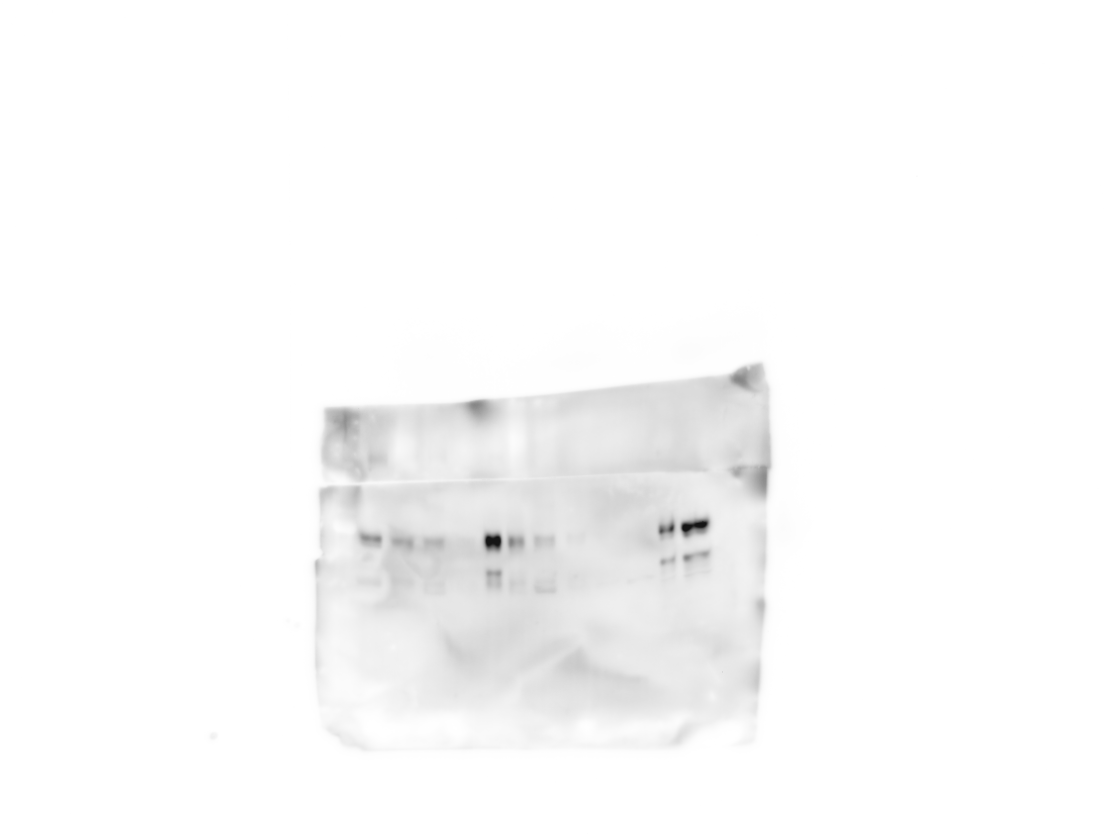

Supplement: MO-021-D4MO00154K-s009 [file MO-021-D4MO00154K-s009.zip › raw image figure s2f yaptaz.tif]

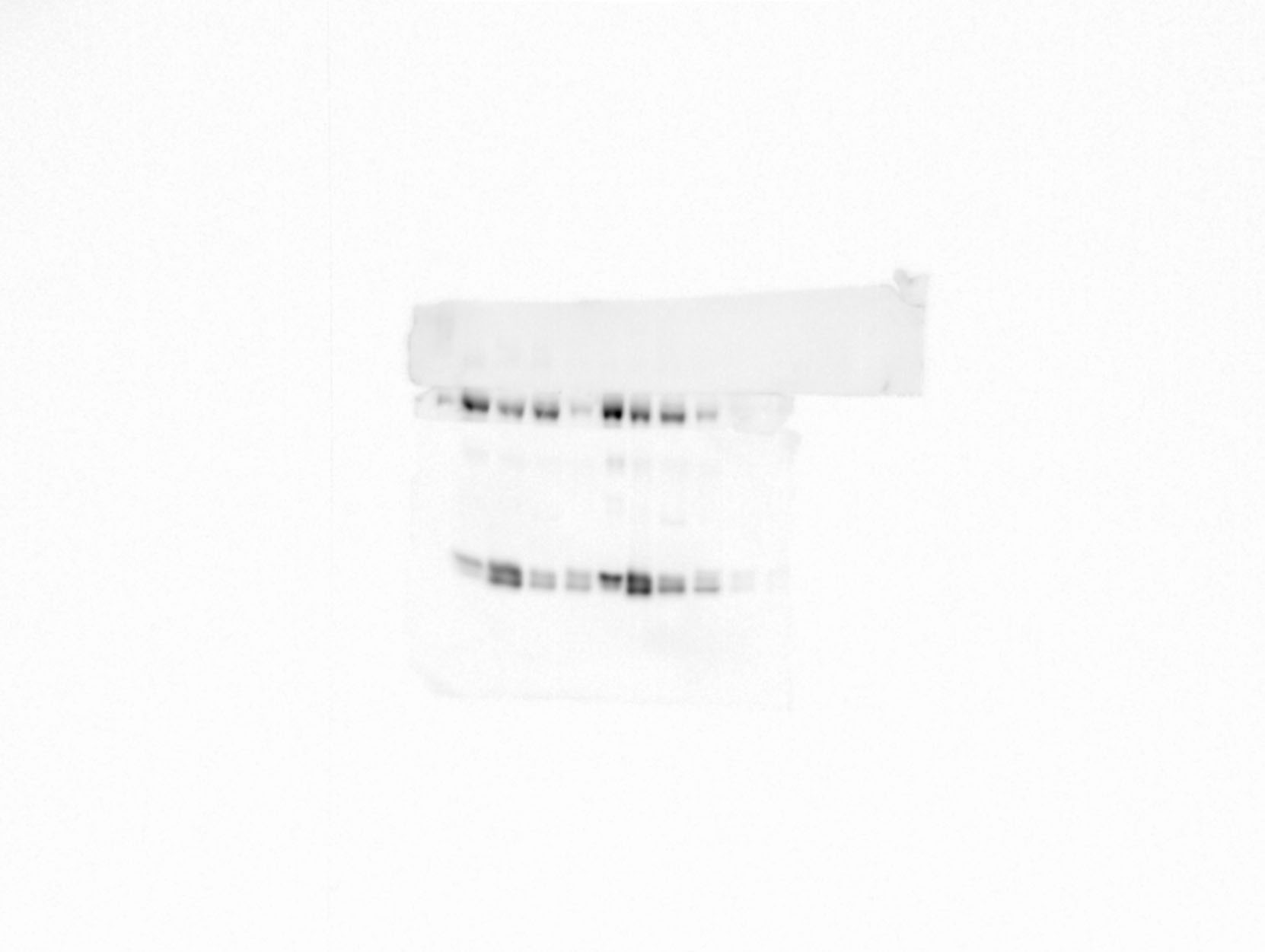

Supplement: MO-021-D4MO00154K-s009 [file MO-021-D4MO00154K-s009.zip › raw image figure s2g pcna_ kif5b.jpg]

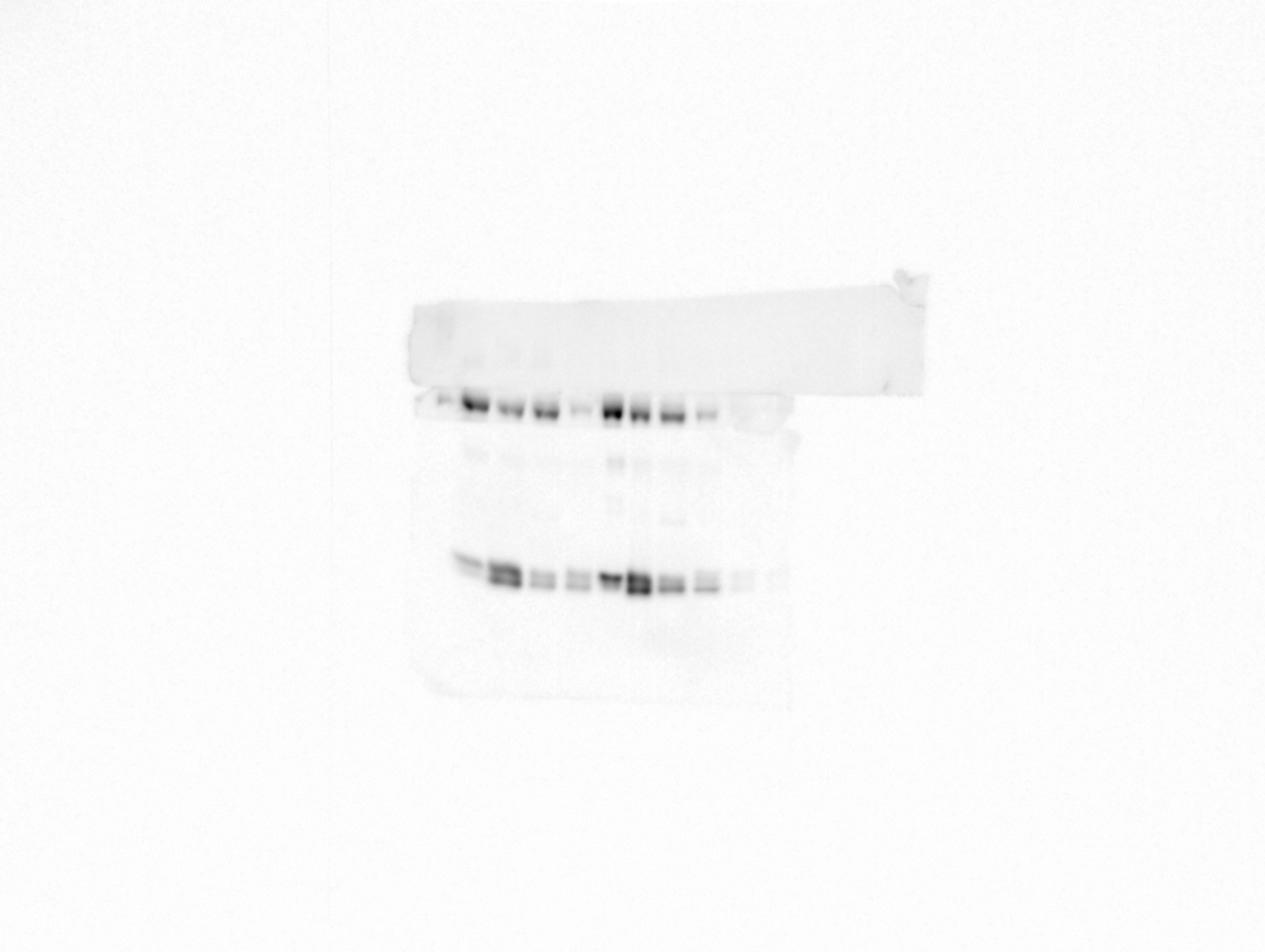

Supplement: MO-021-D4MO00154K-s009 [file MO-021-D4MO00154K-s009.zip › raw image figure s2g pcna_ kif5b.tif]

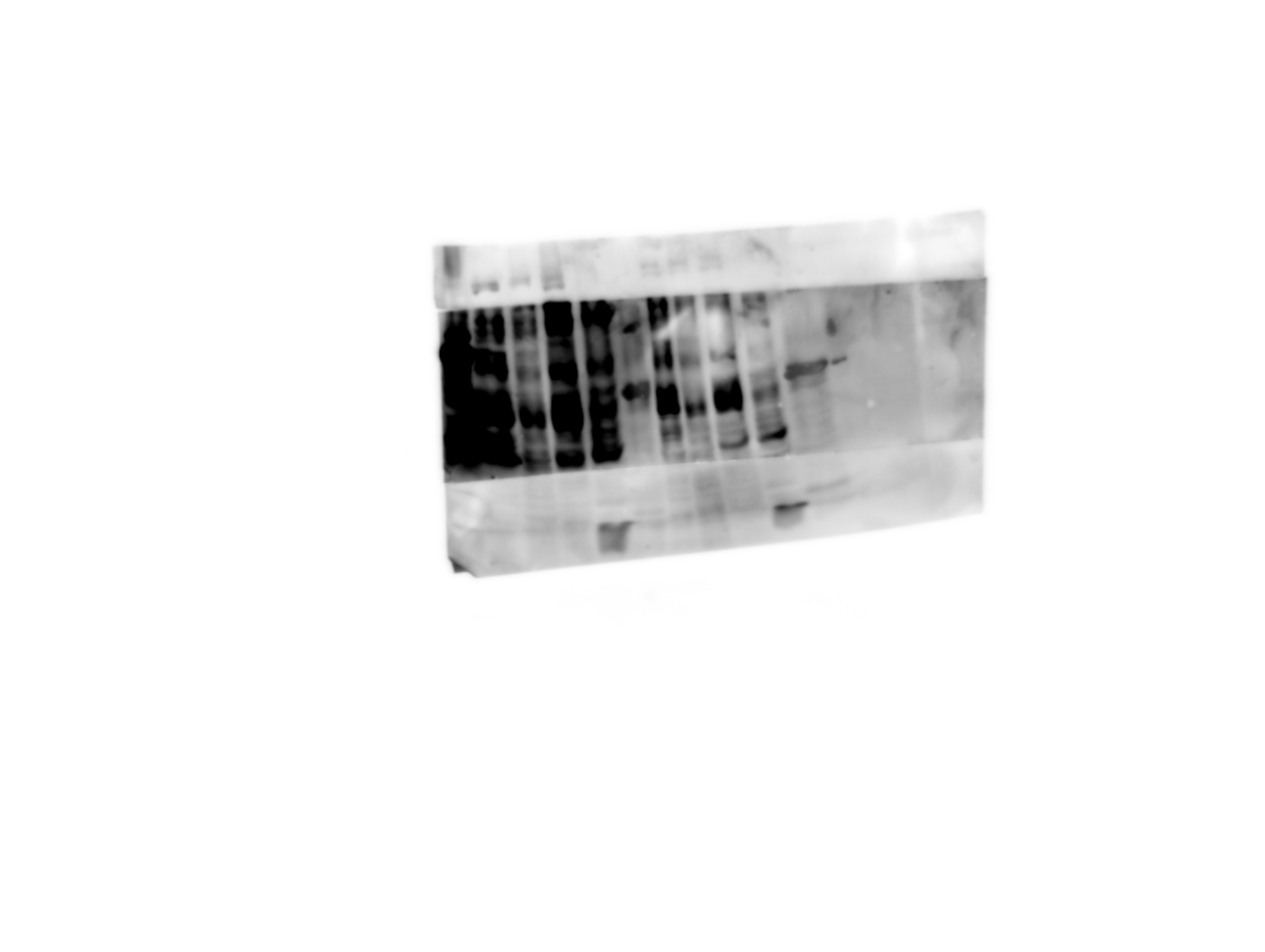

Supplement: MO-021-D4MO00154K-s009 [file MO-021-D4MO00154K-s009.zip › raw image figure s2g ph3b bottom.tif]
