# Supplementary material for: Integration of focal adhesion morphogenesis and polarity by DOCK5 promotes YAP/TAZ-driven drug resistance in TNBC
Source: Mol Omics. 2025 May 12;21(5):390–421. doi: 10.1039/d4mo00154k (PMC12068046; doi:10.1039/d4mo00154k)

Supplementary Information (SI) for Molecular Omics.
This journal is © The Royal Society of Chemistry 2025

Supplementary Figure 6 Beta catenin upregulation in response to LiCl treatment and increased nuclear FOXO3A upon Uprosertib treatment

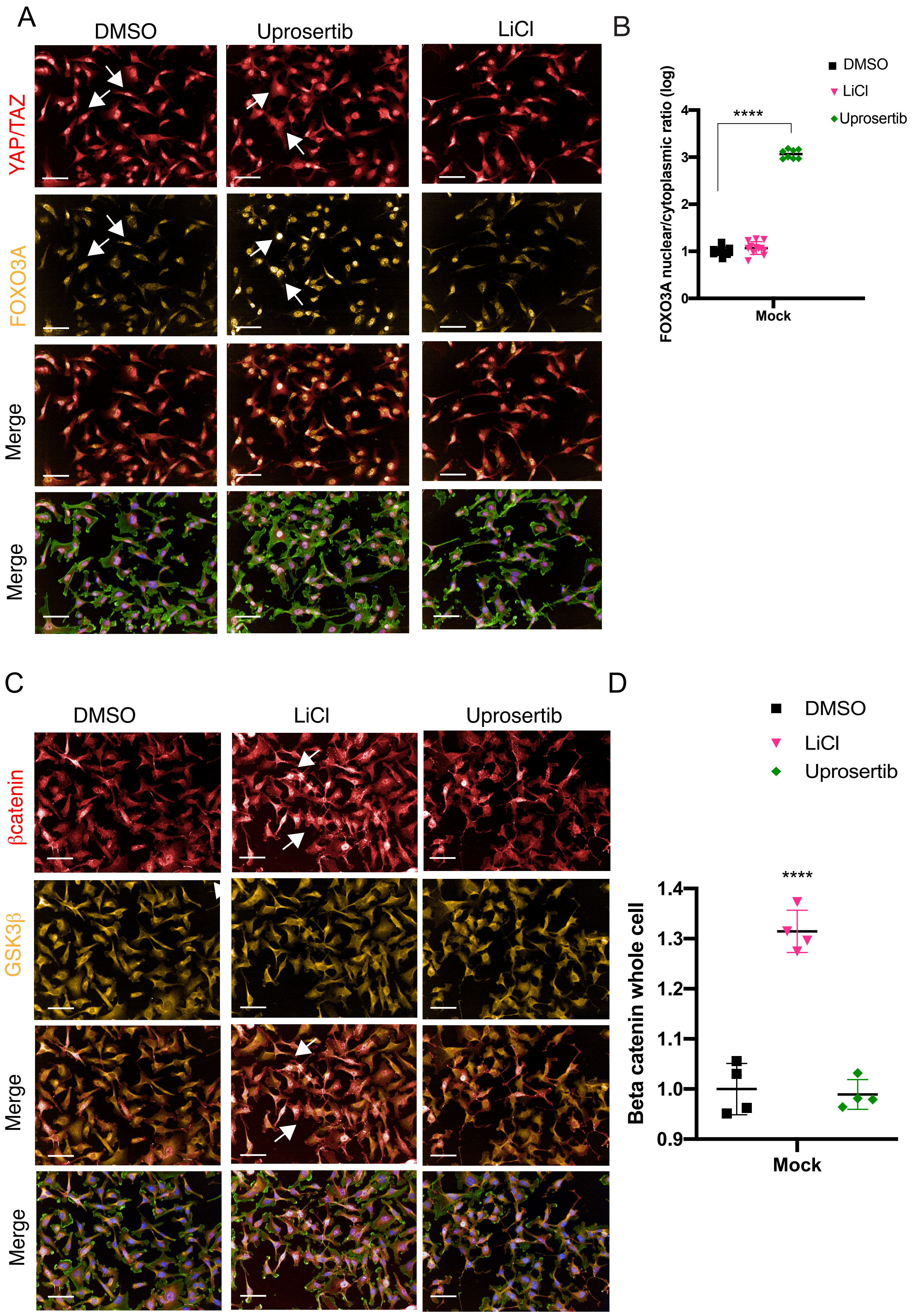

Supplement: MO-021-D4MO00154K-s006 [file MO-021-D4MO00154K-s006.pdf]
