# Supplementary material for: Integration of focal adhesion morphogenesis and polarity by DOCK5 promotes YAP/TAZ-driven drug resistance in TNBC
Source: Mol Omics. 2025 May 12;21(5):390–421. doi: 10.1039/d4mo00154k (PMC12068046; doi:10.1039/d4mo00154k)

Figure S1. YAP/TAZ antibody recognises both YAP and TAZ in immunfluorescence and western blot

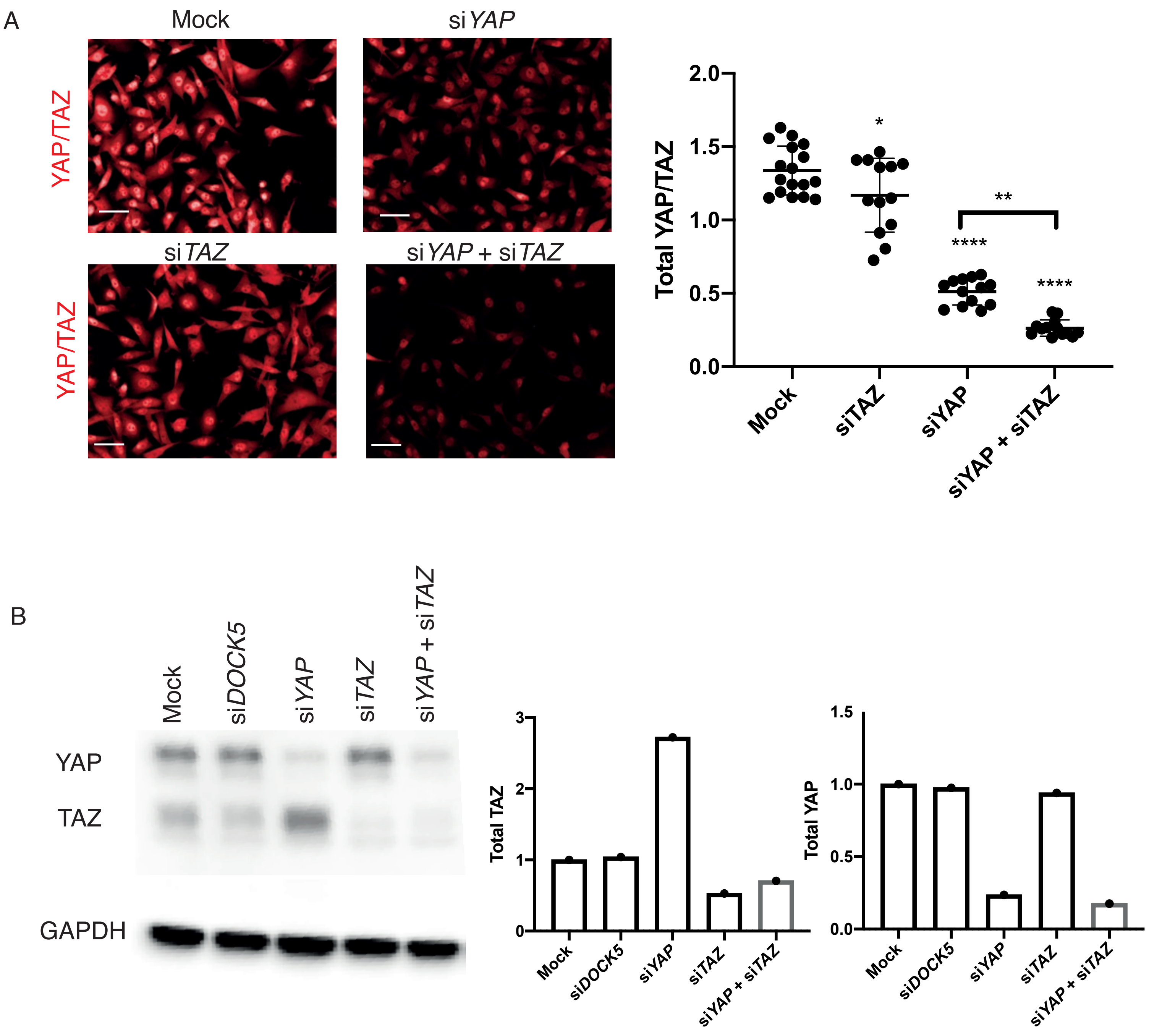

Figure S2. Validation of DOCK5 siRNA knockdown.

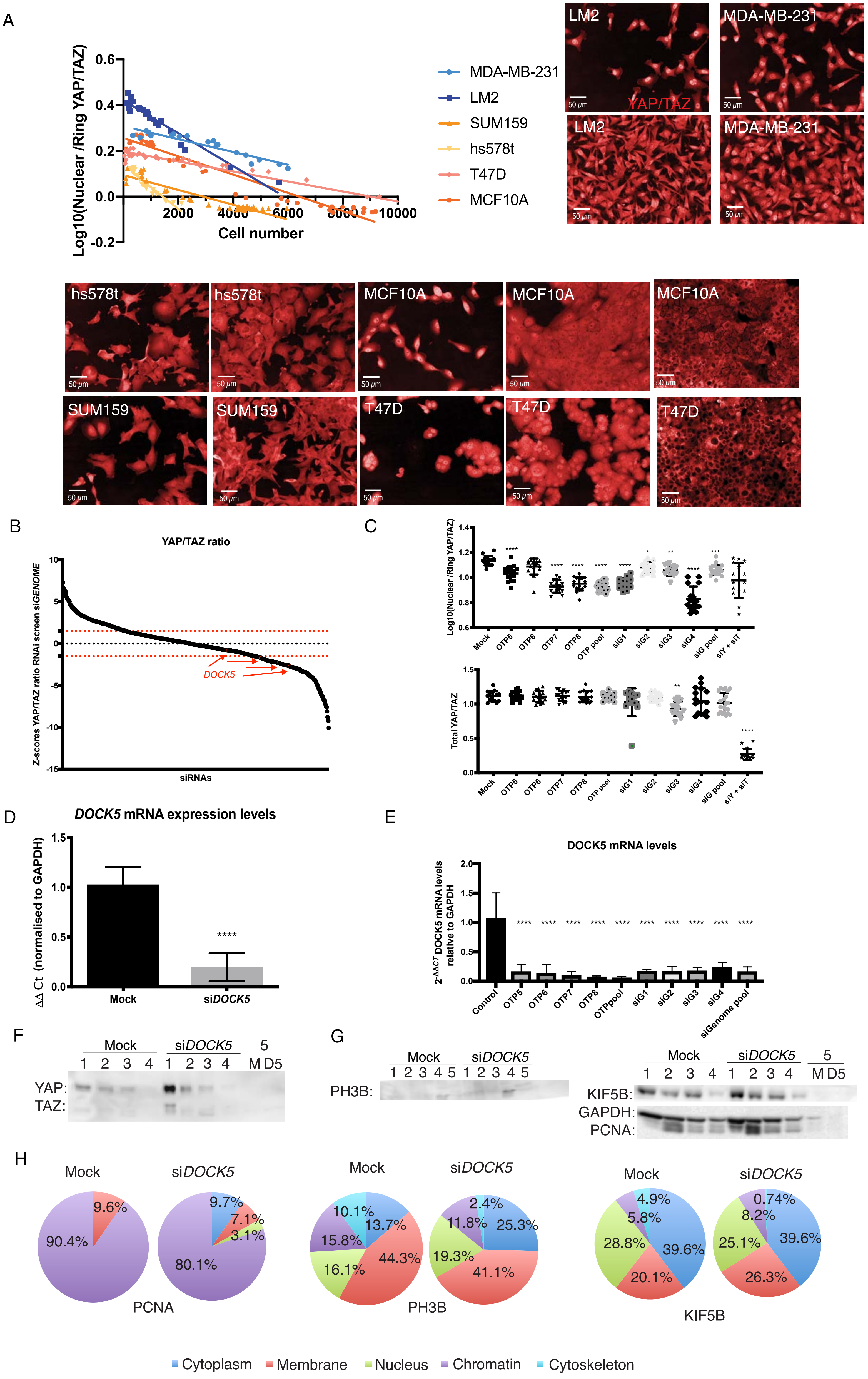

Figure S3. Z-scores for YAP/TAZ ratio in siGENOME and OTP screens in MDA-MB-231.

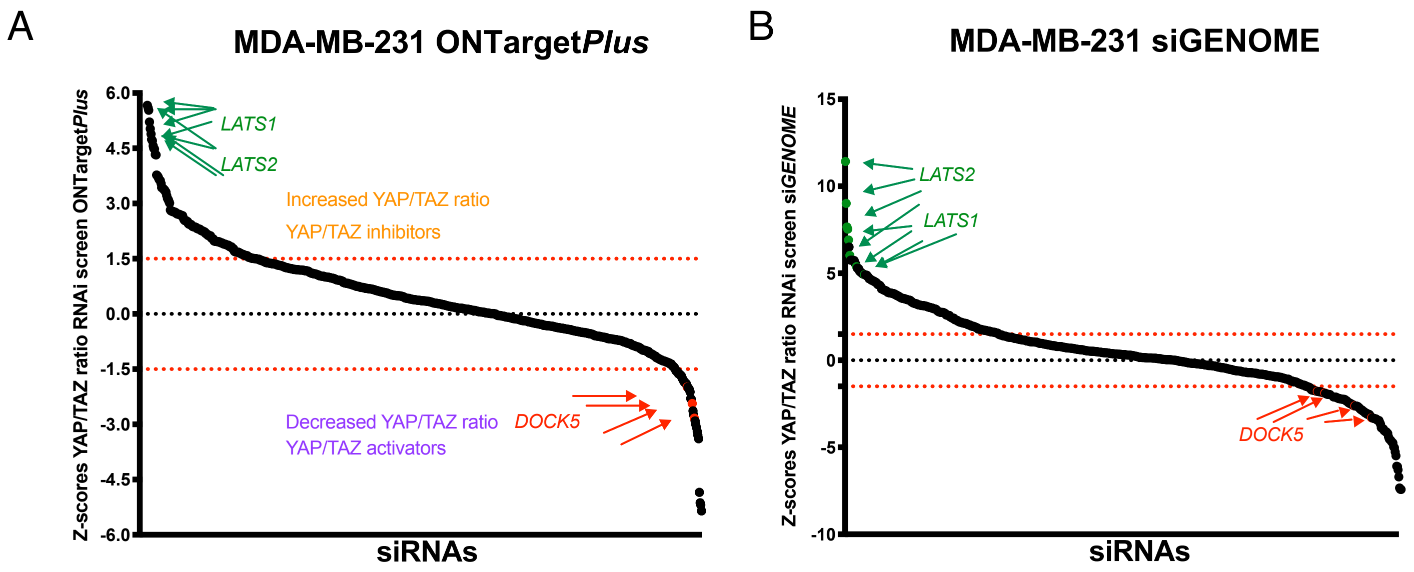

Figure S4. RHOA, RAC1, YAP, TAZ, depletion do not affect growth rates

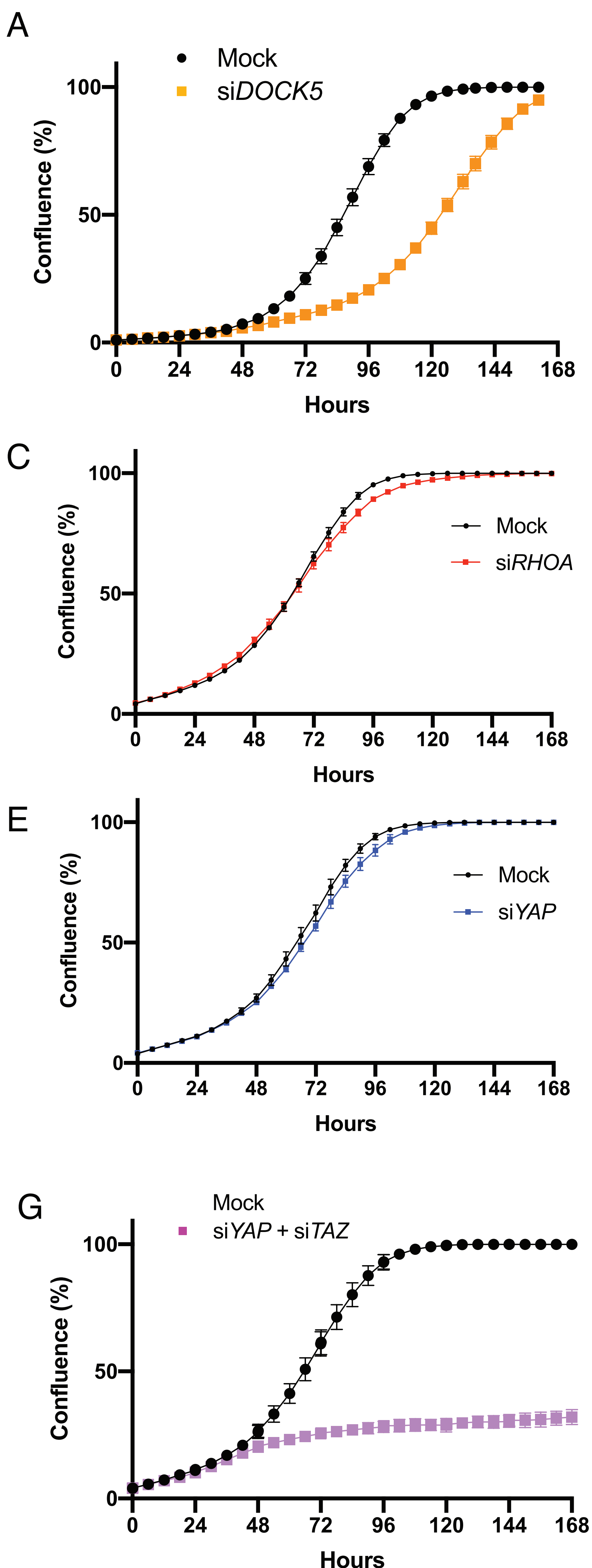

Figure S5. Shape filtering in LM2

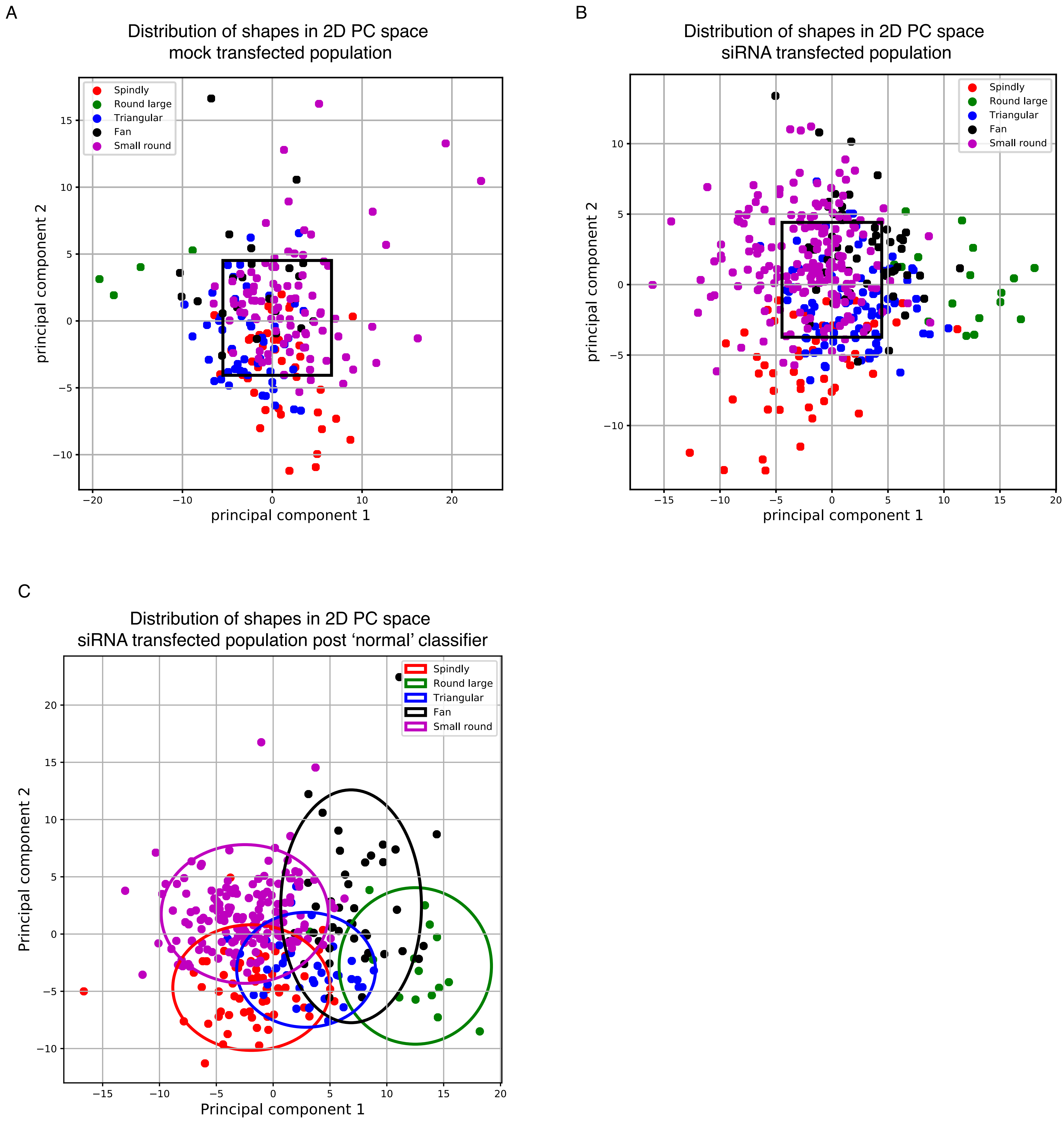

Figure S6. Beta catenin upregulation in response to LiCl treatment and increased nuclear FOXO3A upon Uprosertib treatment

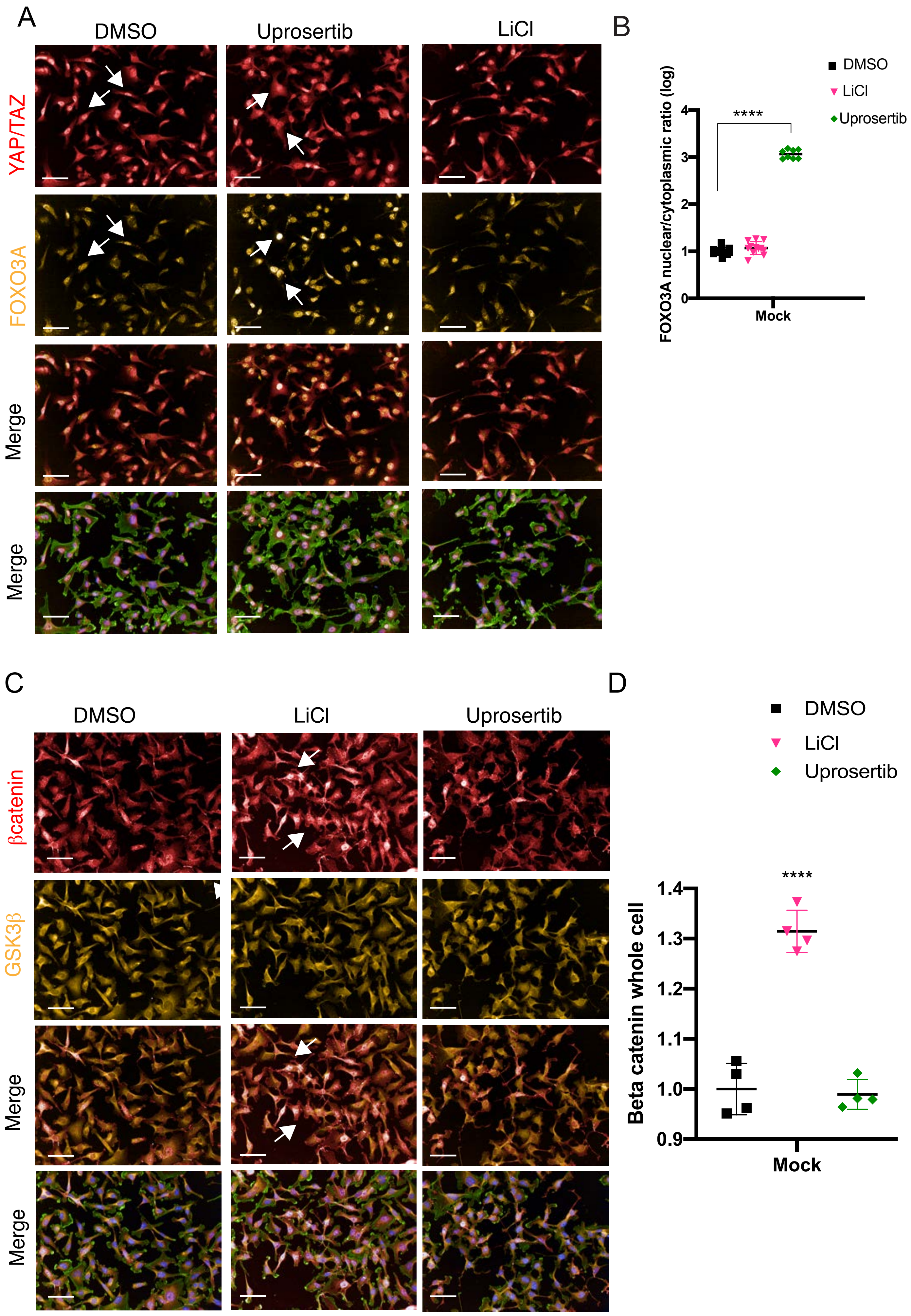

Figure S7. Automated segmentation for Collagen Invasion Quantification

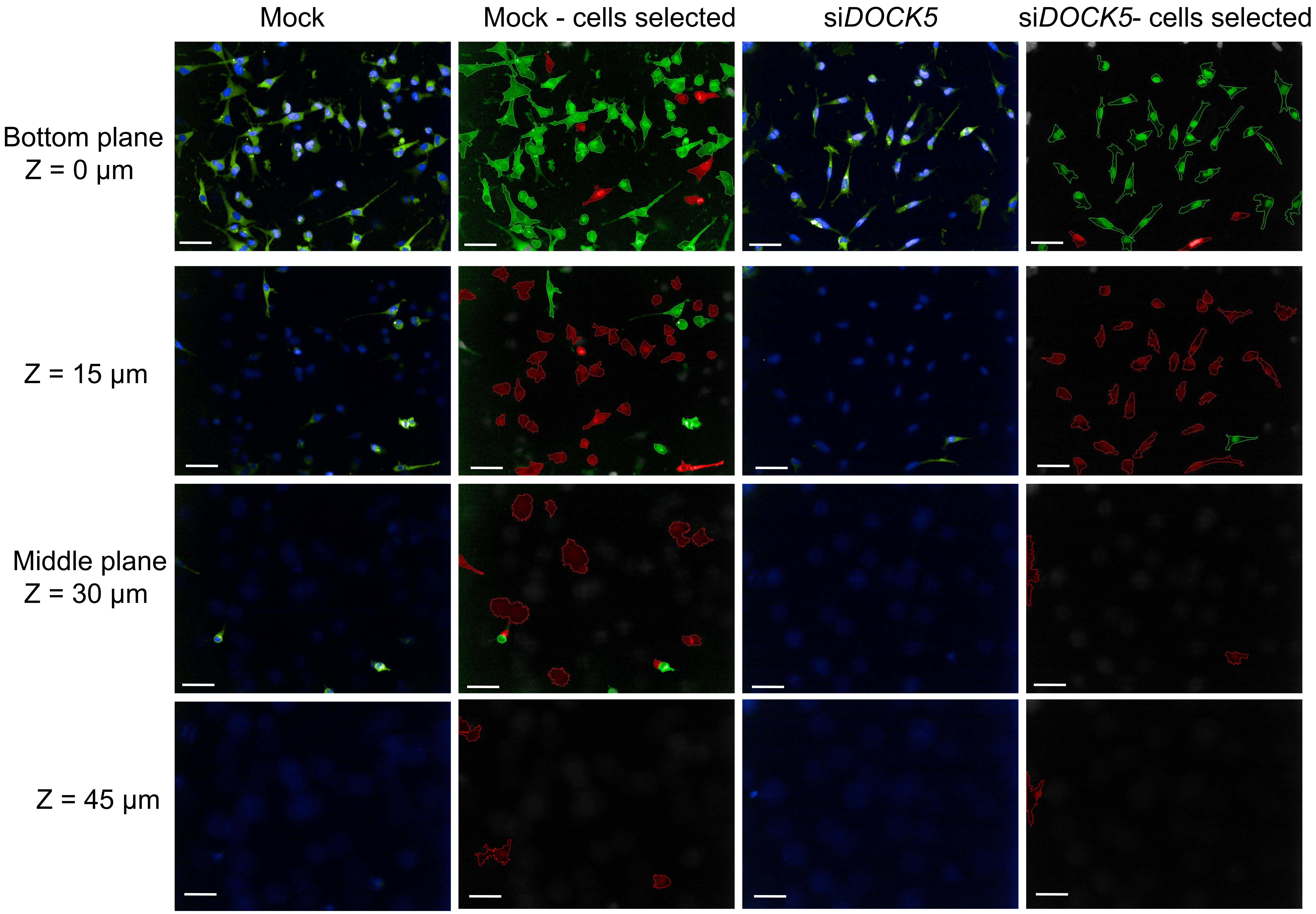

Supplement: MO-021-D4MO00154K-s008 [file MO-021-D4MO00154K-s008.zip › Supplementary Figures, Tables, and Movies/Supplementary Figures DOCK5 Pascual-Vargas et al Molecular Omics Aug 2024.pdf]
