# Supplementary material for: Integration of focal adhesion morphogenesis and polarity by DOCK5 promotes YAP/TAZ-driven drug resistance in TNBC
Source: Mol Omics. 2025 May 12;21(5):390–421. doi: 10.1039/d4mo00154k (PMC12068046; doi:10.1039/d4mo00154k)
Supplement: MO-021-D4MO00154K-s009 [file MO-021-D4MO00154K-s009.zip › pascual vargas et al_annotated uncut blots.pptx]

## Slide 1
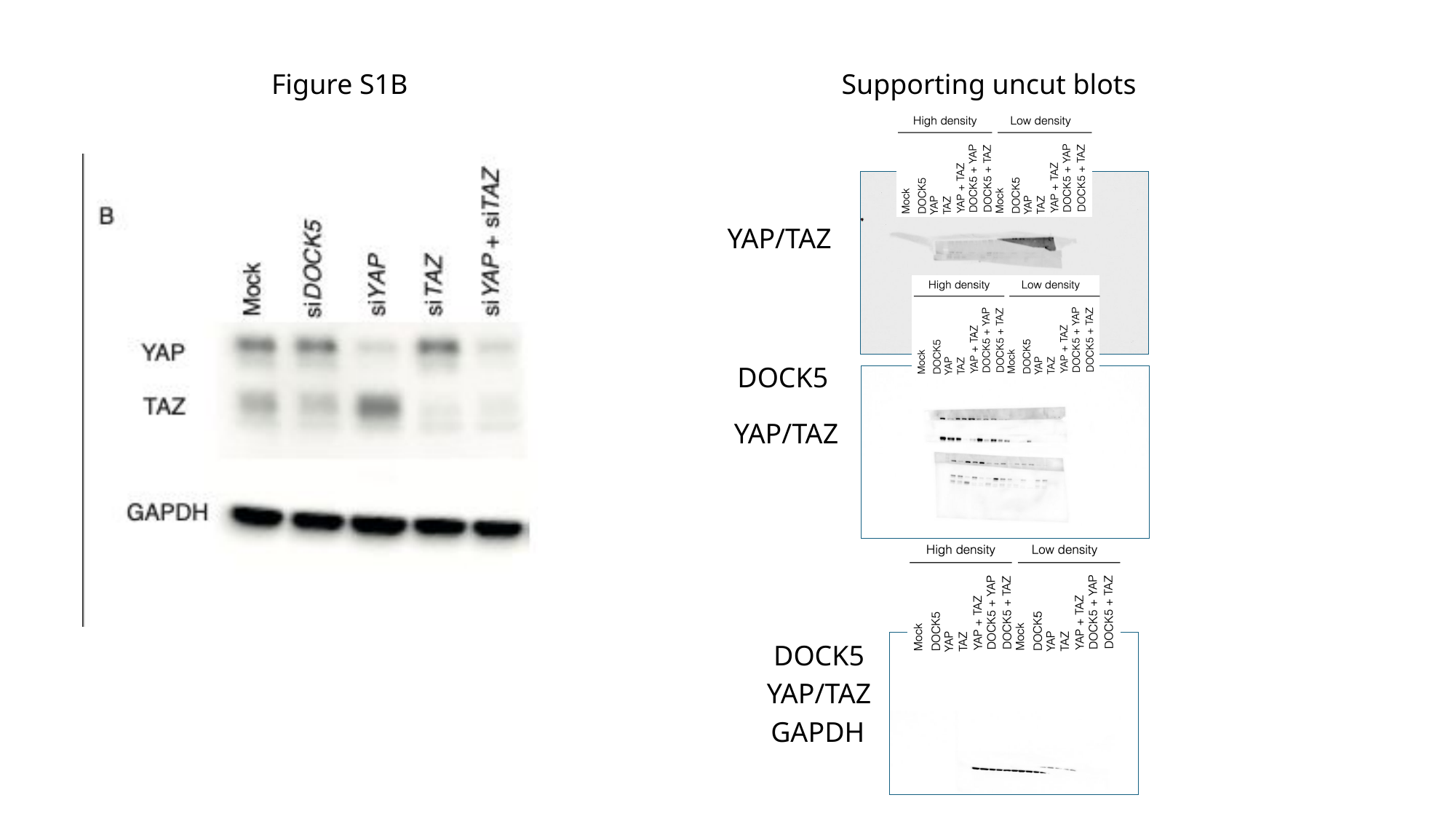

Supporting uncut blots
Figure S1B
YAP/TAZ
DOCK5
YAP/TAZ
DOCK5
YAP/TAZ
GAPDH

## Slide 2
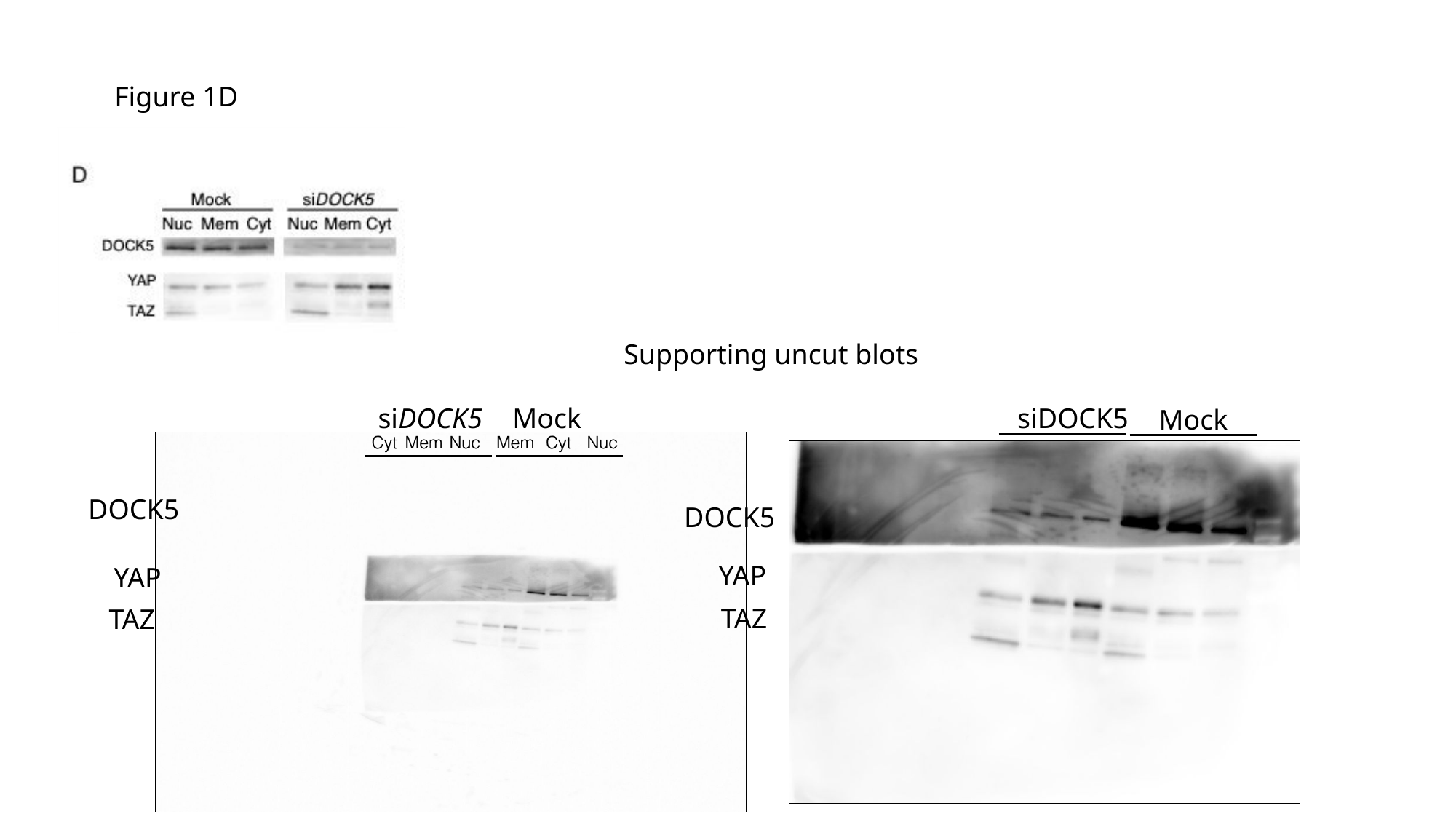

Figure 1D
Supporting uncut blots
Mock
siDOCK5
siDOCK5
Mock
DOCK5
DOCK5
YAP
YAP
TAZ
TAZ

## Slide 3
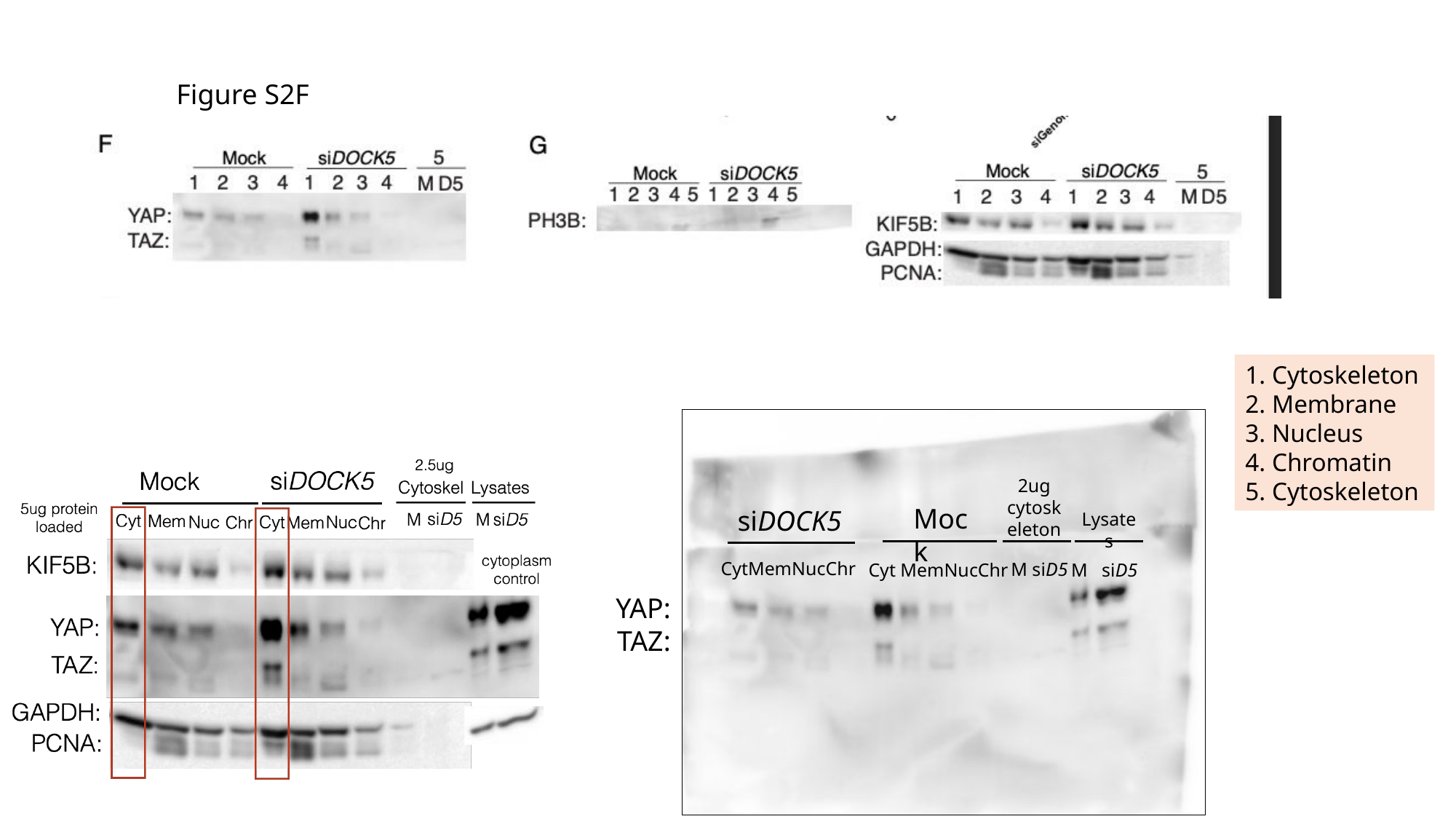

Figure S2F
1. Cytoskeleton
2. Membrane
3. Nucleus
4. Chromatin
5. Cytoskeleton
2ug cytoskeleton
Mock
siDOCK5
Lysates
CytMemNucChr
 M siD5
Cyt MemNucChr
M siD5
YAP:
TAZ:

## Slide 4
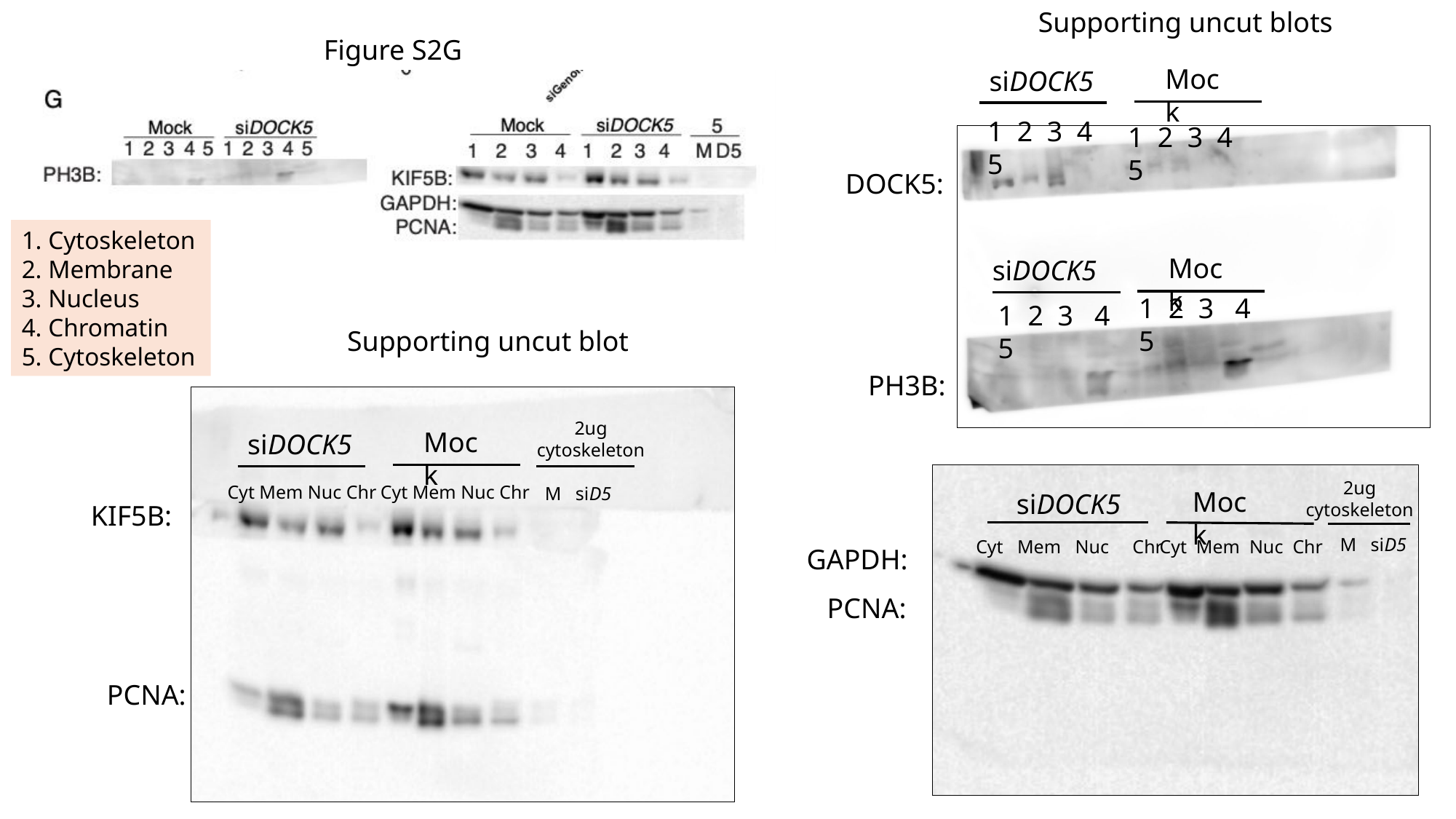

Supporting uncut blots
Figure S2G
Mock
siDOCK5
1 2 3 4 5
1 2 3 4 5
DOCK5:
1. Cytoskeleton
2. Membrane
3. Nucleus
4. Chromatin
5. Cytoskeleton
Mock
siDOCK5
1 2 3 4 5
1 2 3 4 5
Supporting uncut blot
PH3B:
2ug cytoskeleton
Mock
siDOCK5
2ug cytoskeleton
Cyt Mem Nuc Chr
Cyt Mem Nuc Chr
M siD5
Mock
siDOCK5
KIF5B:
M siD5
Cyt Mem Nuc Chr
Cyt Mem Nuc Chr
GAPDH:
PCNA:
PCNA:
